# Thermally accelerated Heck reaction under direct mechanocatalysis using palladium milling balls†

**DOI:** 10.1039/d5mr00032g

**Published:** 2025-05-13

**Authors:** Johanna Templ, Suhmi Hwang, Tino Schwemin, Hakan Baltaci, Lars Borchardt

**Affiliations:** a Ruhr Universität Bochum, Inorganic Chemistry I Universitätsstraße 150 44801 Bochum Germany johanna.templ@tuwien.ac.at lars.borchardt@rub.de

## Abstract

We present a direct mechanocatalytic approach for the Mizoroki–Heck coupling of aryl iodides with olefins using palladium milling balls as catalyst under elevated temperatures in a ball milling reactor. The high chemoselectivity for C(sp^2^)–I bonds ensures a single product when employing multi-halogenated arenes. Additionally, a one-pot Wittig olefination/Heck cross-coupling enables direct access to stilbene derivatives from aldehydes and aryl iodides without intermediate isolation.

## Abstract 2

We report a thermally accelerated, air–tolerant Mizoroki–Heck reaction catalyzed by a palladium milling ball. This direct mechanocatalytic approach features facile catalyst recycling, high C(sp^2^)–I bond selectivity, and operational simplicity.

## Graphical abstract

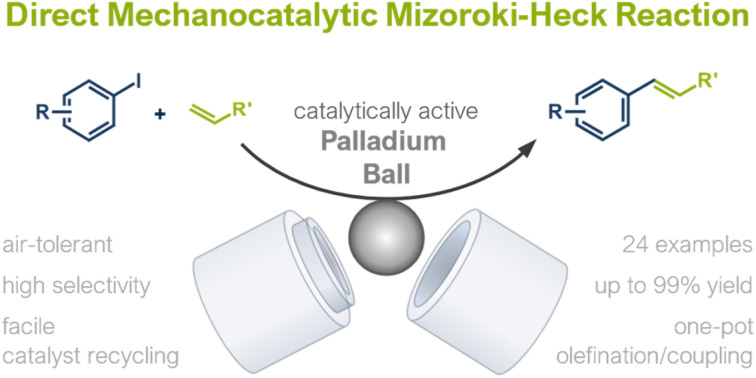

## Introduction

Since their discovery, palladium-catalyzed cross-coupling reactions have become indispensable strategies for the formation of carbon–carbon and carbon–heteroatom bonds in modern synthetic chemistry. Among them, the Mizoroki–Heck reaction stands out as one of the most powerful methods for C(sp^2^)–C(sp^2^) bond formation, enabling the synthesis of aryl-substituted alkenes with broad applications in pharmaceuticals, agrochemicals, and materials science.^1–6^ The disubstituted double bond formed in this reaction can serve as a versatile handle for further functionalization, including hydrogenation, epoxidation, or cyclization, expanding its synthetic utility even further.

**Fig. 1:**
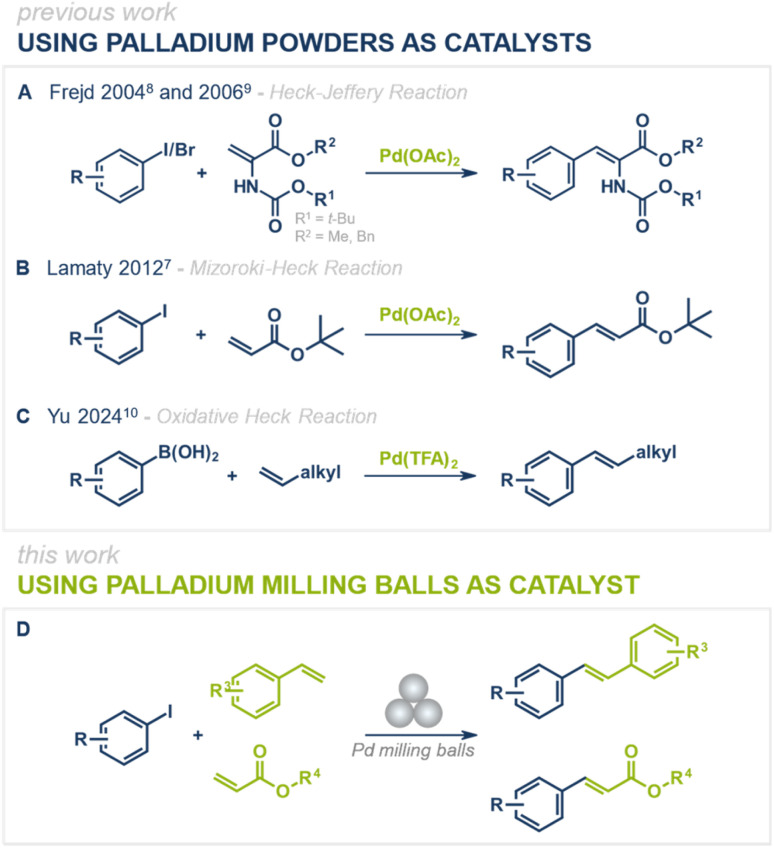
State of the art of mechanochemical Heck reactions using palladium powders as catalysts (A–C) and the herein presented Heck reaction under direct mechanocatalysis using palladium milling balls as catalyst (D).

While conventional Mizoroki–Heck reactions are typically performed under solution-based conditions, recent advances have focused on making these transformations more environmentally benign by eliminating the need for organic solvents and instead employing mechanochemical techniques (Fig. 1A–C).^7–10^ Over the past few years, various mechanochemical protocols for palladium-catalyzed cross-coupling reactions have been reported, including the Negishi,^11^ Buchwald–Hartwig,^12–16^ Suzuki-Miyaura,^17–29^ Sonogashira,^30–33^ Tsuji–Trost,^34^ and Mizoroki–Heck^7–10^ reactions.^35^ While these protocols efficiently run under solvent-free or liquid-assisted grinding (LAG) conditions, they still add the catalyst in its powdered form, which after reaction is finely dispersed in the reaction mixture. Upon product isolation (*e.g.*, filtration or liquid–liquid extractions), the catalyst often becomes either inactive or efficient catalyst isolation is not feasible, limiting its recyclability and reuse. A promising solution to this challenge is direct mechanocatalysis, where the metallic milling media itself (*e.g.*, milling balls or jars) serves as the active catalyst.^36–41^ This strategy eliminates the need for external catalyst additives, simplifies the purification process, and enhances catalyst recyclability. Unlike conventional approaches, reagents and products can be easily separated from the catalyst simply by removing the milling balls from the reaction mixture, allowing for straightforward catalyst reuse without loss of activity. Previous studies have successfully demonstrated the applicability of palladium milling balls for Suzuki–Miyaura^42–45^ and Sonogashira^46^ couplings, showing the potential of direct mechanocatalysis in palladium-catalyzed C–C bond-forming reactions. However, a feasible protocol for a direct mechanocatalytic Mizoroki–Heck reaction has remained elusive. With the protocol presented herein, we aim to close this gap and further expand the toolbox of palladium-catalyzed cross-coupling reactions under mechanochemical conditions (Fig. 1D).

## Results and discussion

**Fig. 2:**
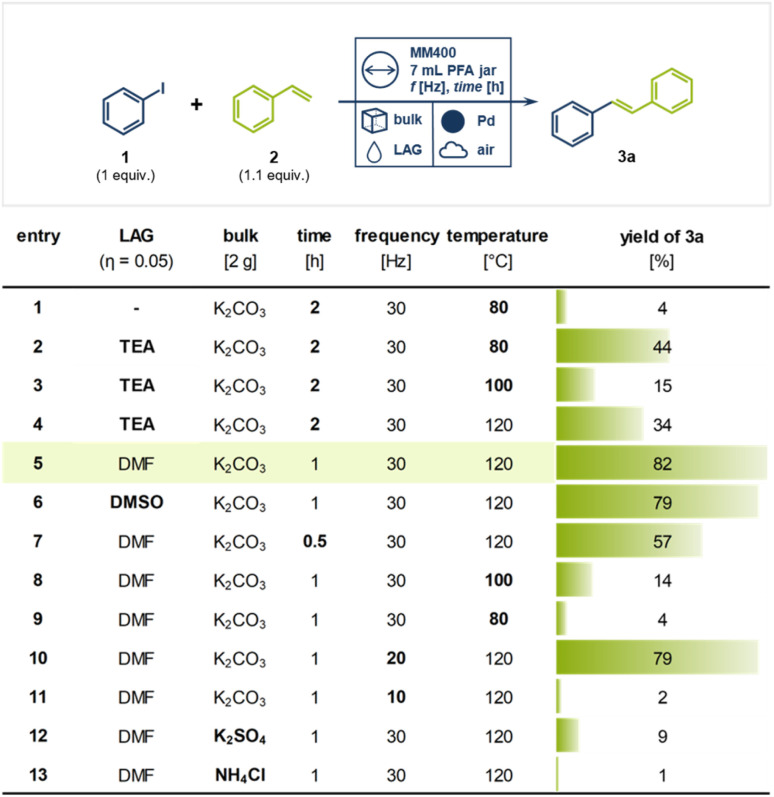
Parameter screening for the direct mechanocatalytical Mizoroki–Heck reaction of iodobenzene (1) and styrene (2) giving (*E*)-stilbene (3a). Deviation from standard conditions (entry 5) are given in bold. Standard conditions: Pd milling ball (10 mm), PFA vessel (7 mL), 2 g bulking agent, 1 (1 mmol, 1 equiv.), 2 (1.1 mmol, 1.1 equiv.), DMF (100 μL); MM400 Retsch, 30 Hz, 2 h, 120 °C external temperature. Yield determined *via* quant. ^1^H-NMR using dibromomethane as internal standard.

Building upon our prior research on palladium milling media-catalyzed cross-coupling reactions,^40,44,46^ we initiated our studies using a single milling ball of pure palladium to catalyze the coupling of a well-established substrate system in Mizoroki–Heck reactions: iodobenzene (1) and styrene (2) (Fig. 2). To prevent palladium abrasion and ensure that catalytic conversion occurs on the surface of the metallic milling media, we employed perfluoroalkoxy alkanes (PFA) as a chemically and thermally stable and rather “soft” polymeric milling jar material.^47^ Additionally, the use of potassium carbonate as a bulking agent in approximately 10-fold excess effectively eliminated palladium abrasion.^38,46^ Catalyst leaching experiments further confirmed that the catalytic conversion was not mainly mediated by abraded palladium particles but rather by the active milling media surface (see ESI† for details).^48^ Thermal control of the reaction was facilitated by electrically heated metal jackets surrounding the PFA milling vessels (for details see ESI†). Initially, iodobenzene (1, 1 equiv.), styrene (2, 1.1 equiv.) and 2 g of K_2_CO_3_ were ball milled for 2 h at 80 °C under air (Fig. 2, entry 1), yielding only 4% of the desired (*E*)-stilbene (3a). The addition of an equimolar amount triethylamine (TEA, 1 equiv.) as LAG agent (*η* = 0.05) significantly improved the yield of 3a to 44% (entry 2). However, increasing the temperature to 100 °C and 120 °C resulted in lower yields (*cf.* 15%, entry 3 and 34%, entry 4), likely due to TEA's boiling point (89 °C) causing its rapid evaporation at higher reaction temperatures. Switching to higher boiling solvents such as dimethyl formamide (DMF) or dimethyl sulfoxide (DMSO) enabled smooth reactions at 120 °C, affording the product in 82% (entry 5) and 79% (entry 6) yield, respectively, within 60 minutes. Reducing the reaction time further to 30 minutes lowered the yield of 3a to 57% due to incomplete conversion (entry 7).

Interestingly, when the reaction temperature for the DMF-containing system was reduced to 100 or 80 °C, the yields dropped significantly, falling to 14% (entry 8) and 4% (entry 9), respectively. This decline can likely be attributed to the increased solubility of K_2_CO_3_ in hot DMF.^49^ As the base becomes more soluble, it may be more readily available to facilitate the reaction at 120 °C. However, at temperatures below 100 °C, the reaction mixture may approach a more neutral pH, which could reduce the effectiveness of the base. In contrast, when TEA was used as the LAG agent, the reaction still proceeded with moderate efficiency, providing the product in 44% yield even at 80 °C (*cf.* entry 2). This suggests that TEA's inherent basic properties accelerate the reaction more effectively at lower temperatures.

Interestingly, the milling frequency played a critical role: while 25 and 20 Hz provided comparable yields (entry 10), reducing the frequency to 10 Hz nearly shut down the reaction (2% yield, entry 11). These findings suggest that the reaction is not solely thermally driven but requires a minimum mechanical energy threshold. No product was observed without milling at 120 °C or at 30 Hz at room temperature, indicating that the reaction is both thermally and mechanically driven. The choice of bulking agent also proved crucial—mildly basic K_2_CO_3_ was optimal, whereas KCl and KOAc led to “snowballing” effects that covered the catalytic surface, and K_2_SO_4_ and NH_4_Cl created unfavorable non-basic environments (entries 12 and 13).

**Fig. 3:**
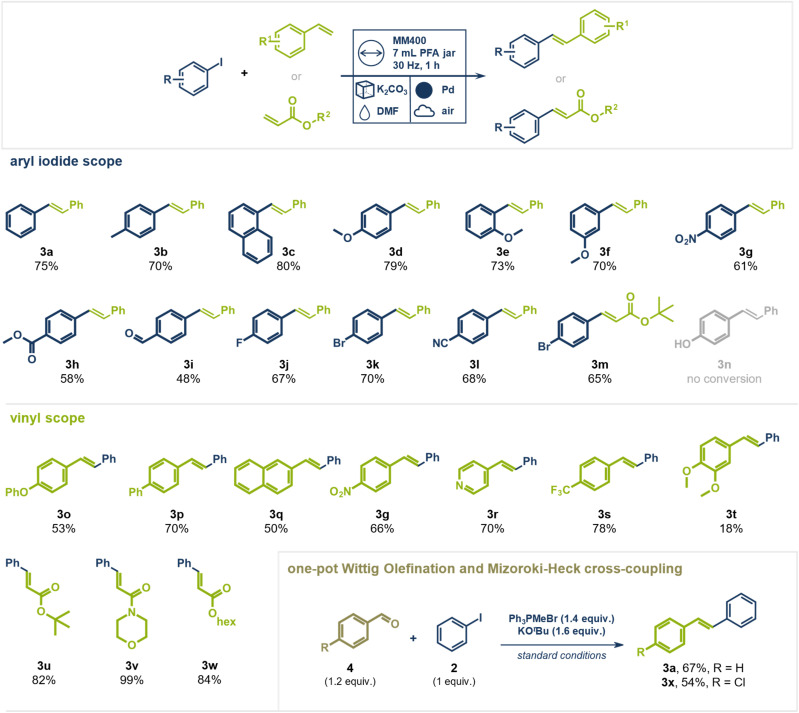
Scope for the Mizoroki–Heck reaction under direct mechanocatalysis and the one-pot Wittig olefination/Mizoroki–Heck cross-coupling. Isolated Yields are given. Conditions for Mizoroki–Heck reaction: Pd milling ball (10 mm), PFA vessel (7 mL), 2 g bulking agent, aryl iodide (1 mmol, 1 equiv.), vinyl derivative (1.1 mmol, 1.1 equiv.), DMF (100 μL); MM400 Retsch, 30 Hz, 2 h, 120 °C external temperature. For the one-pot Wittig olefination/Mizoroki–Heck reaction, the vinyl compound was exchanged for an aldehyde (1.2 equiv.) and PPh_3_MeBr (1.4 equiv.) and KO^*t*^Bu (1.6 equiv.) were added.

Having identified the best performing reaction conditions, we investigated the generality of coupling partners in this direct mechanocatalytic conversion (Fig. 3). A range of substituted aryl iodides coupled readily with styrene yielding the respective (*E*)-stilbene analogues in up to 80%. Electron donating methoxy groups were well tolerated at different positions on the aromatic system giving the desired products in 70–79% yield (products 3d–3f). Aryl iodides bearing electron withdrawing nitro (product 3g, 61%), halide (product 3j, 67%, 3k, 70%, and 3m, 65%), aldehyde (product 3i, 48%), ester (product 3h, 58%), and nitrile groups (product 3l, 68%) could be coupled with styrene in moderate to good yields. Phenol-containing substrates were unsuccessful, likely due to strong interactions of oxygen with palladium, which blocked the catalytically active sites (product 3n not observed, starting material fully recovered). This hypothesis is supported by a control experiment in which substoichiometric amounts (0.5 equiv.) of 4-hydroxybiphenyl were added to the standard reaction (aryl iodide with styrene), resulting in only 12% yield of *trans*-stilbene (3a) alongside unreacted starting material (1), with no additional by-products detected (details see ESI†). Notably, aryl chlorides and bromides did not undergo coupling, highlighting the high chemoselectivity of C(sp^2^)–I activation. This contrasts with solution-based Mizoroki–Heck reactions, where frequently both aryl iodides and bromides react. The selective nature of this transformation was further demonstrated using mixed halogenated substrates. For example, 1-bromo-4-iodo benzene coupled with styrene (product 3k, 70%) or *tert*-butyl acrylate (product 3m, 65%) exclusively *via* the C(sp^2^)–I bond, leaving the C(sp^2^)–Br bond intact. This distinct selectivity is a key advantage over traditional solution-based methods, which often yield complex product mixtures when multiple halogens are present. This feature could enable a synthetic sequence where a Heck coupling *via* C(sp^2^)–I is followed by a different cross-coupling *via* the C(sp^2^)–Br bond present in the substrate without requiring intermediate functional group addition or conversion. Building on our previous work demonstrating successful Suzuki couplings of aryl bromides under direct mechanocatalysis, we propose that oxidative addition is generally feasible for both aryl bromides and iodides.^44^ In contrast to the Suzuki reaction, the Mizoroki–Heck reaction lacks a transmetalation step, leading us to hypothesize that Pd(ii)–Br species may impede a later stage of the catalytic cycle—likely migratory insertion—whereas Pd(ii)–I do not. However, further mechanistic studies are needed to identify the rate-determining step and clarify the influence of the halogen substituent in direct mechanocatalytic Mizoroki–Heck reactions.

Expanding the scope beyond styrene, other vinyl derivatives were examined. Both electron-withdrawing (products 3p, 3g, and 3s) and electron-donating (product 3o) groups were well tolerated. Even heterocyclic vinyl pyridine gave the desired product 3r in 70% yield. However, a di-methoxy substituted styrene derivative reacted sluggishly, giving product 3t only a poor yield of 18% with significant amounts of unreacted starting material remaining. In contrast, acrylates reacted readily with aryl iodides giving the products in high yields and purity. *Tert*-butyl (product 3u, 82%) and hexyl acrylate (product 3w, 84%), as well as acryloyl morpholine (product 3v, 99%) gave the desired products without requiring additional purification steps. Again, 1-bromo-4-iodo-benzene coupled exclusively *via* its C(sp^2^)–I bond with *tert*-butyl acrylate, leaving the C(sp^2^)–Br unaltered (product 3m, 65%).

To further enhance the synthetic utility of this method, we explored a sequential olefination/cross-coupling protocol for the direct synthesis of stilbene derivatives from aldehydes, without the need to isolate the olefin intermediate under mechanochemical conditions. We envisioned this one-pot conversion by pairing a mechanochemical Wittig olefination—recently reported by Templ and Schnürch—with the direct mechanocatalytic Heck reaction.^50^ Traditional Wittig olefinations use alkyl triphenylphosphonium ylides to form olefins, producing stoichiometric amounts of triphenylphosphine oxide as a by-product. These organo-phosphorus species could interfere with the palladium catalyst, reducing its activity and complicating the one-pot approach.^51–53^ However, we hypothesized that in our direct mechanocatalytic process, the palladium milling media might be less prone to interacting with “ligand-like” organo-phosphorous species compared to homogeneous palladium catalysts in solution, thus allowing a prior Wittig olefination to be tolerated. To test this, we combined benzaldehyde, Ph_3_PMeBr, KO^*t*^Bu, and iodobenzene in a PFA milling jar, subjecting the mixture to the optimized reaction conditions for Heck coupling. (*E*)-Stilbene (3a) was successfully obtained from iodobenzene and benzaldehyde in 67% yield over two steps in an operationally simple setup, without the need for inert atmosphere in either step. Additionally, 4-chloro-stilbene (3x) could be isolated in 54% yield from iodobenzene and 4-chloro-benzaldehyde. This one-pot Wittig olefination/Heck cross-coupling procedure could prove especially useful when vinyl arenes are too reactive for storage and transport and must be synthesized *in situ* from precursors.

## Conclusions

We have developed a practical protocol for direct mechanocatalytic Mizoroki–Heck coupling, utilizing a single palladium milling ball as the catalytically active media under elevated temperatures. This approach offers the advantage of operationally simple catalyst removal and recycling. The method exhibits excellent chemoselectivity for C(sp^2^)–I coupling, leaving other C(sp^2^)–X bonds (X = F, Cl, Br) intact. It enables the efficient synthesis of various aryl alkenes within just one hour of reaction time, with a broad tolerance for functional groups. Furthermore, we demonstrated the compatibility of the direct mechanocatalytic Heck protocol with a preceding olefination step. This one-pot procedure enables the *in situ* formation of vinyl coupling partners from aryl aldehydes *via* a Wittig reaction, providing a straightforward route to aryl-coupled derivatives without the need for intermediate olefin isolation.

## Author contributions

J. T., S. H., L. B. wrote the manuscript jointly. J. T., S. H., T. S., and H. B. conducted synthetic experiments and screenings. J. T. and L. B. performed the administration, conceptualization and supervision of the project. All the authors have read and agreed to the published version of the manuscript.

## Conflicts of interest

There are no conflicts to declare.

## Supplementary Material

MR-002-D5MR00032G-s001

## Data Availability

The data supporting this article have been included as part of the ESI.†
