# Supplementary material for: Thermally accelerated Heck reaction under direct mechanocatalysis using palladium milling balls
Source: RSC Mechanochem. 2025 May 13;2(4):598–602. doi: 10.1039/d5mr00032g (PMC12100728; doi:10.1039/d5mr00032g)
Supplement: MR-002-D5MR00032G-s001 [file MR-002-D5MR00032G-s001.pdf]

Supplementary Information (SI) for RSC Mechanochemistry.
This journal is © The Royal Society of Chemistry 2025

Supporting Information

Thermally Accelerated Heck Reaction under Direct Mechanocatalysis using Palladium Milling Balls

Johanna Templ,*[a]† Shumi Hwang,[a]† Tino Schwemin,[a] Hakan Baltaci[a] and Lars Borchardt*[a]

<u>Contents</u>

1. **General Remarks:** S3
2. **General Procedures** S4
   General Procedure A – Optimization Screening for the Mizoroki-Heck coupling S4
   General Procedure B – Mizoroki-Heck coupling *via* direct mechanocatalysis S4
   General Procedure C – One-pot Wittig olefination/Heck cross-coupling S4
3. **Complete Parameter Screening Tables:** S5
4. **Compound Synthesis and Characterization:** S8
   (E)-1,2-Diphenylethene (3a) S8
   (E)-1-Methyl-4-styrylbenzene (3b) S8
   (E)-2-Styrlnaphthalene (3c) S8
   (E)-1-Methoxy-4-styrylbenzene (3d) S8
   (E)-1-Methoxy-2-styrylbenzene (3e) S9
   (E)-1-Methoxy-3-styrylbenzene (3f) S9
   (E)-1-Nitro-4-styrylbenzene (3g) S9
   (E)-Methyl 4-styrylbenzoate (3h) S9
   (E)-4-Styrylbenzaldehyde (3i) S10
   (E)-1-Fluoro-4-styrylbenzene (3j) S10
   (E)-1-Bromo-4-styrylbenzene (3k) S10
   (E)-1-Cyano-4-styryl-benzene (3l) S10
   Tert-Butyl *(E)*-3-(4-bromophenyl)acrylate (3m) S11
   (E)-1-Phenoxy-4-styrylbenzene (3o) S11
   (E)-4-Phenylstilbene (3p) S11
   (E)-2-Styrylnaphthalene (3q) S11
   (E)-1-Nitro-4-styrylbenzene (3g) *from 4-nitrovinyl benzene* S12
   (E)-4-Styrylpyridine (3r) S12
   (E)-1-Styryl-4-(trifluoromethyl)benzene (3s) S12
   (E)-3,4-Dimethoxystilbene (3t) S12
   Tert-Butyl cinnamate (3u) S13
   N-Morpholinocinnamide (3v) S13
   Hexylcinnamate (3w) S13
   (E)-Stilbene (3a) *from benzaldehyde* S13
   (E)-1-Chloro-4-styrylbenzene (3x) *from 4-chlorobenzaldehyde* S13
5. **NMR Spectra** S15
6. **References:** S39

1. General Remarks:

General. All reagents were obtained from commercial suppliers at least in synthesis grade purity and were used without further purification. Organic solvents used for LAG were obtained in analysis grade. Water used for LAG was taken from a tap in deionized form. The milling was carried out exclusively in a Retsch MM-400 ball mill. The milling vessels were custom made from PFA. The heating setup was custom made for the Retsch MM-400 (right) using heating jackets from Ihne & Tesch (230-250V, 300 W, CrNi-Ni) and a control panel for temperature adjustments (left).

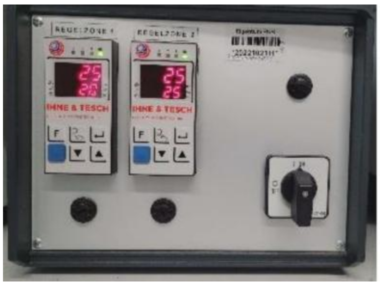

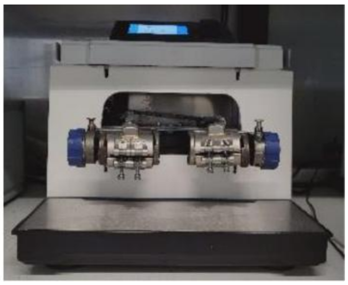

^{1}H NMR, ^{13}C NMR, and ^{19}F NMR spectra were recorded on a Bruker Avance III HD spectrometer at 400 MHz at ambient temperature. Chemical Shifts (δ) are reported in ppm. Coupling constants (J) are given in Hertz (Hz) and multiplicities are assigned as s (singlet), d (doublet), t (triplet), q (quartet), m (multiplet), and combinations thereof.

Column chromatography was performed on standard manual glass columns using Merck silica gel 60 (40 µm – 63 µm). Thin Layer Chromatography (TLC) analysis was performed on aluminum-backed unmodified Merck silica gel 60 F245 plates. Visualization was realized under UV irradiation or *via* heat staining using $KMnO_4$ solution.

Milling media. Palladium milling balls were obtained from SMT Metalle Wimmer in Weinburg, Austria. Due to the manufacturing process of these milling balls (arc sintering), they are not perfectly spherical but flattened. The diameter is measured at the broadest part of the milling ball. Polymer material for manufacturing of PFA Vessels were obtained from Kelux in Geldern, Germany.

X-ray fluorescence spectroscopy (XRF). The XRF-spectra were obtained by a Vanta™ Handheld XRF Analyzer with a 40 keV Rhodium X-ray tube.

2. General Procedures

General Procedure A – Optimization Screening for the Mizoroki-Heck coupling

A 7 mL PFA vessel was equipped with a single 4-5 g milling ball of pure palladium metal (10 mm diameter, not perfectly spherical). 2 g K_2CO_3 were loaded on top of the milling ball. Then the aryl iodide (1 mmol, 1 equiv.), the aryl alkene (1.1 mmol, 1.1 equiv.) and 100 µL of the LAG agent were added. The vessel was closed and mounted into the heating jackets (Ihne & Tesch, 300 W) and the holding station of the MM-400 Retsch mixer mill. The temperature was adjusted to the desired reaction temperature. As soon as the temperature was reached (approx. 2-3 minutes), the milling process was started, and milling was continued for the given time at the respective frequency. After the reaction, the vessel was cooled to room temperature and 1.5 mL CDCl3 were added. 70 µL (1 mmol) dibromomethane were added *via* Hamilton syringe and all components were mixed thoroughly. Approx. 1 mL of the mixture was filtered over a short plug of celite directly into an NMR tube. NMR was recorded on a Bruker 400 MHz machine with a prolonged relaxation delay (D1) of 5 seconds. Quantification analysis was conducted *via* peak integral calculations using MestReNova Software (Version 14.2.0).

D1 relaxation times were determined via reference measurements to ensure reproducible measurements. An exemplary NMR spectra can be found on page S6 (Figure S1).

General Procedure B – Mizoroki-Heck coupling *via* direct mechanocatalysis

A 7 mL PFA vessel was equipped with a single 4-5 g milling ball of pure palladium metal (10 mm diameter, not perfectly spherical). 2 g K_2CO_3 were loaded on top of the milling ball. Then the aryl iodide (1 mmol, 1 equiv.), the aryl alkene (1.1 mmol, 1.1 equiv.) and 100 µL DMF were added. The vessel was closed and mounted into the heating jackets (Ihne & Tesch, 300 W) and the holding station of the MM-400 Retsch mixer mill. The temperature was adjusted to 120 °C. As soon as the desired temperature was reached (approx. 2-3 minutes), the milling process was started, and milling was continued for 1 hour at 30 Hz. After the reaction, the vessel was cooled to room temperature and water and DCM were added. The mixture was transferred to a separation funnel and the product was extracted 3 times with DCM (approx. 15 mL each). The combined organic phases were washed once with brine, dried over $MgSO_4$, filtered and concentrated to obtain the crude cross-coupled product.
For all reactions the abrasion of the milling ball was below 1 mg.

General Procedure C – One-pot Wittig olefination/Heck cross-coupling

A 7 mL PFA vessel was equipped with a single 4-5 g milling ball of pure palladium metal (10 mm diameter, not perfectly spherical). 1.6 equiv. of KO^{t}Bu (1.6 mmol) and 2 g K_2CO_3 were loaded on top of the milling ball. Then the aryl iodide (1 mmol, 1 equiv.), the aldehyde (1.2 mmol, 1.2 equiv.), methyltriphenylphosphonium bromide (1.4 mmol, 1.4 equiv.) and 100 µL DMF were added.

The vessel was closed and mounted into the heating jackets (Ihne & Tesch, 300 W) and the holding station of the MM-400 Retsch mixer mill. The milling process was started and the temperature was adjusted to 120 °C. Milling was continued for 1 hour at 30 Hz. After the reaction, the vessel was cooled to room temperature and water and a 1:1 mixture of Et_2O:*n*-pentane were added. The mixture was transferred to a separation funnel and the product was extracted 3 times with the Et_2O:*n*-pentane mixture (approx. 15 mL water and organic solvents each). The combined organic phases were washed once with water and brine, dried over $MgSO_4$, filtered and concentrated to obtain the crude cross-coupled product.

For all reactions the abrasion of the milling ball was below 1 mg.

Alternatively, EtOAc can be used during workup procedures for product extraction.

3. Complete Parameter Screening Tables:

Table S1. Full table for reaction parameter screening. Experiment for optimization screening were performed following the general procedure A

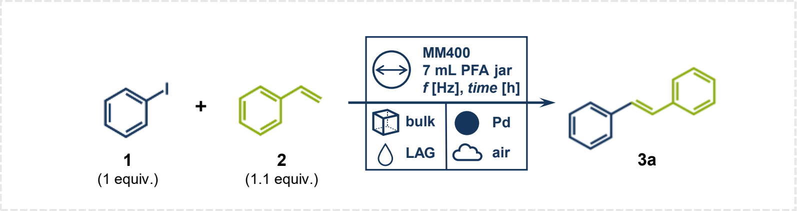

entry	LAG (η = 0.05)	bulk [2 g]	time [h]	frequency [Hz]	temperature [°C]	yield of 3a [%]	recovery of 1 [%]
1	-	K_2CO_3	**2**	30	**80**	4	83
2	**TEA**	K_2CO_3	**2**	30	**80**	44	41
3	**TEA**	K_2CO_3	**2**	30	**100**	15	70
4	**TEA**	K_2CO_3	**2**	30	120	34	25
**5**	DMF	K_2CO_3	1	30	120	82	9
6	**DMSO**	K_2CO_3	1	30	120	79	10
7	DMF	K_2CO_3	**0.5**	30	120	57	26
8	DMF	K_2CO_3	1	30	**100**	14	86
9	DMF	K_2CO_3	1	30	**80**	4	96
10	DMF	K_2CO_3	1	**20**	120	79	0
11	DMF	K_2CO_3	1	**10**	120	2	55
12	DMF	**K_2SO_4**	1	30	120	9	86
13	DMF	**NH_4Cl**	1	30	120	1	84
14	DMF	**KCl**	1	30	120	8	83
15	DMF	**KOAc**	**2**	30	**100**	2	88
17	DMF	K_2CO_3	1	**25**	120	77	13
18	DMF	K_2CO_3	1	**0**	120	0	77
19	**TEA (1 equiv.)**	K_2CO_3 (2g)	**2**	**35**	**r.t.**	10	83
20	**H_2O (2 equiv.)**	K_2CO_3 (2g)	**2**	**35**	**r.t.**	31	56
21	**$H_2O/CDCl_3$ (1 equiv.)**	K_2CO_3 (2g)	**2**	**35**	**r.t.**	22	65
22	DMF (1.3 equiv.)	K_2CO_3 (2g)	1	30	**r.t.**	0	99
23	DMF (1.3 equiv.)	K_2CO_3 (2g)	**2**	30	**100**	28	66
24[a]	DMF (1.3 equiv.)	K_2CO_3 (2g)	1	30	120	0	98
25	DMF/H_2O(1.3 equiv.)	K_2CO_3 (2g)	1	30	120	77	0
26	DMF (1.3 equiv.)	zeolite (2g)	1	30	120	19	48
27[b]	TEA (1 equiv.)	K_2CO_3 (2g)	2	30	80	49	16
28[b]	DMF (1.3 equiv.)	K_2CO_3 (2g)	1	30	100	9	46
29[b]	DMF (1.3 equiv.)	K_2CO_3 (2g)	1	30	80	2	74

entry	LAG (η = 0.05)	bulk [2 g]	time [h]	frequency [Hz]	temperature [°C]	yield of 3a [%]	recovery of 1 [%]
**30**	**diethyl carbonate (0.8 equiv.)**	K_2CO_3 (2g)	1	30	120	22	66
**31**	**γ-valerolactone (1.1 equiv.)**	K_2CO_3 (2g)	1	30	120	28	66
**32**	**water (5.5 equiv.)**	K_2CO_3 (2g)	1	30	**100**	8	92
**33**	**tributyl amine (0.4 equiv.)**	K_2CO_3 (2g)	1	30	120	16	36
**34**	DMF (1.3 equiv.)	**Li_2CO_3 (2g)**	1	30	120	19	44
**35**	DMF (1.3 equiv.)	**Na_2CO_3 (2g)**	1	30	120	6	91
**36**	DMF (1.3 equiv.)	K_2CO_3 (2g)	1	**35**	120	83	0
**37**	DMF (1.3 equiv.)	K_2CO_3 (2g)	1	30	**150**	80	0
**38**[c]	DMF (1.3 equiv.)	K_2CO_3 (2g)	1	30	120	12	88

Yields were determined by quant. ^{1}H-NMR using 1 mmol dibromomethane as internal standard. [a] A single 10 mm ZrO_2 ball was used instead of a palladium milling ball. [b] A workup was conducted before quant. ^{1}H-NMR measurements (see remark below). [c] 0.5 equiv of 4-hydroxy biphenyl [CAS: 92-69-3] were added.

<u>Remark</u>: To verify that a workup via liquid/liquid extraction is not influencing the yield of the reaction, for some experiments (entries 27-29) a workup procedure in analogy to the workup described in the general procedure B was followed. After workup and solvent evaporation, the internal NMR standard (dibromomethane, 1 mmol) and 1.5 mL $CDCl_3$ were added to the crude product. After mixing thoroughly, approx. 1 mL of the solution was transferred to an NMR tube and an NMR spectrum was recorded with a prolonged relaxation time (D1) of 5 seconds.

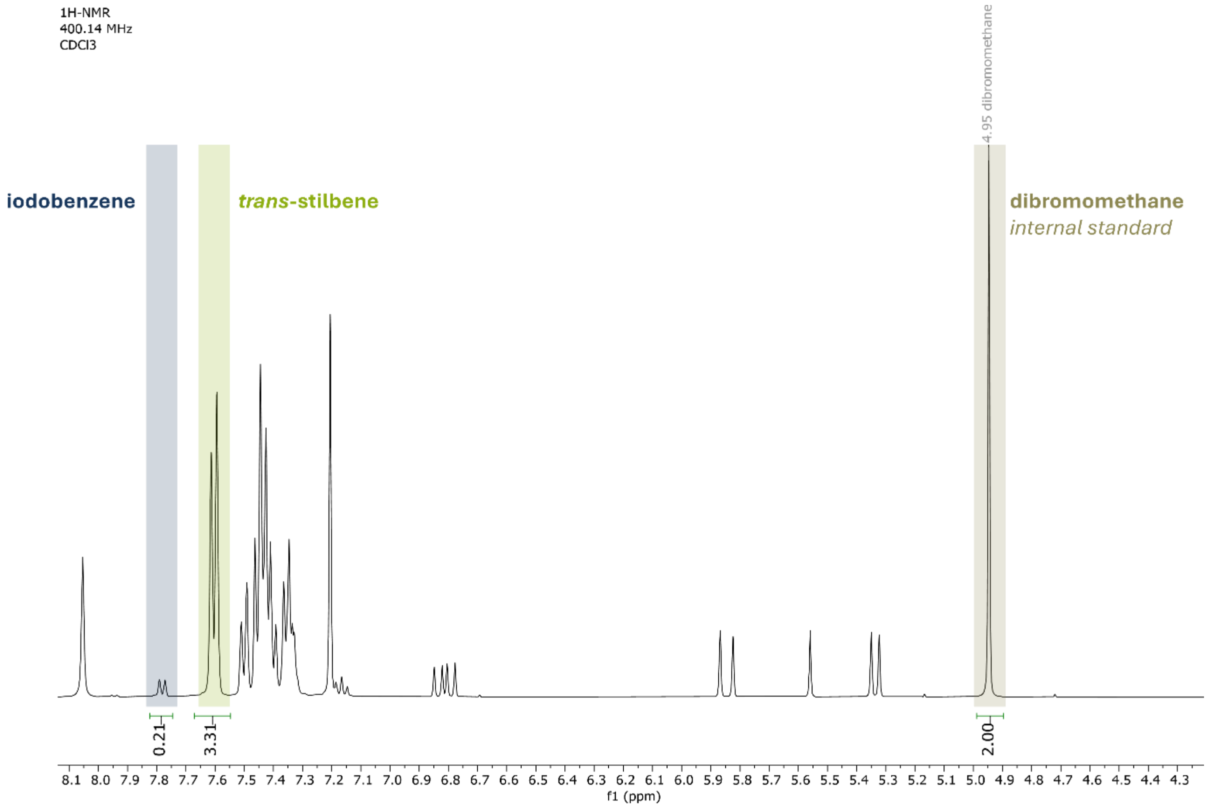

Figure S1. Example for quant. ^{1}H-NMR spectrum using dibromomethane as the internal standard.

Catalyst Leaching Experiment:

To determine whether the catalytic turnover primarily occurs on the surface of the metallic milling media or is facilitated by abraded palladium particles, a catalyst leaching experiment was conducted. A 7 mL PFA vessel was charged with a palladium milling ball (10 mm) and 100 µL DMF (*note: A control experiment in which DMF was omitted in this initial step but added later yielded comparable results*). The vessel was then mounted in the heating jackets and holding station of the MM400 Retsch ball mill, and milling was performed for 30 minutes at 120 °C and 30 Hz. After this initial milling period, the reaction mixture was cooled, and the Pd milling ball was replaced with an inert ZrO_2 ball (10 mm). The substrates (1 mmol iodobenzene, 1.1 mmol styrene) were then added, and milling was resumed under identical conditions (120 °C, 30 Hz) for an additional hour. Quantitative ^{1}H-NMR analysis, using 1 mmol of dibromomethane as an internal standard, revealed that only 10% of the *trans*-stilbene product (**3a**) was formed, with most of the starting material recovered. These findings suggest that while the presence of abraded palladium particles cannot be entirely ruled out as a contributing factor, the primary catalytic turnover likely occurs directly on the surface of the metallic milling media. Notably, palladium abrasion after the initial 30 minutes of milling was minimal, measuring less than 0.5 mg.

XRF measurements of the inner walls of the PFA milling vessels confirmed that no palladium abrasion or incorporation into the soft polymeric vessel material had occurred.

Recycling of the Palladium Milling Balls

For all experiments, we used only three different palladium milling balls, which were recycled throughout the entire study. After each reaction, the milling balls were removed from the reaction mixture, rinsed with water and acetone to eliminate residual reactants, and dried using a paper towel. They were then immediately reused in subsequent reactions. No visible changes to the surface of the milling balls were observed after use (see Figure S2), and the rinsing procedure served solely to remove any remaining reaction materials.

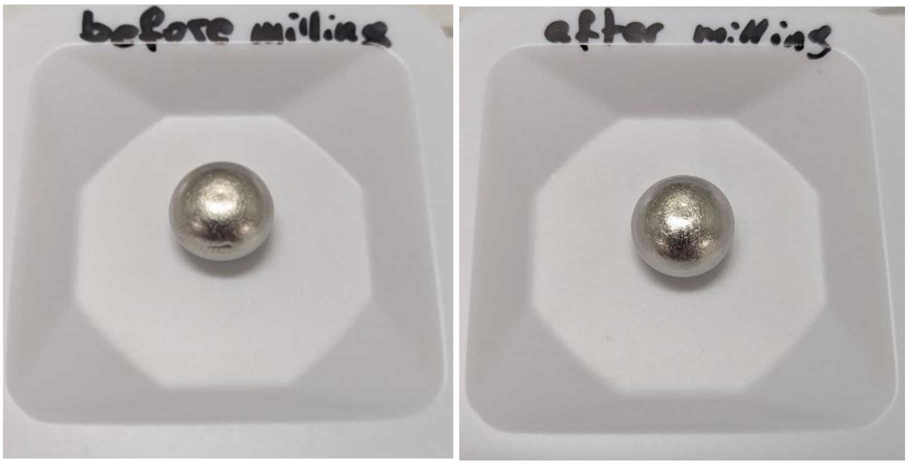

Figure S2. Palladium milling ball before (left) and after (right) milling. No alteration of the milling ball surface detectable.

4. Compound Synthesis and Characterization:

***(E)*-1,2-Diphenylethene (3a)**

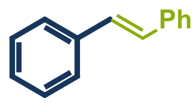

Prepared following the general procedure B from commercially available starting material. The crude product was purified *via* column chromatography (dry load, 12 g silica, hex:EtOAc 50:1) to yield 136 mg (75%) of the title compound as a white solid.

Analytical data is in accordance with literature.[1]

^{1}H NMR (300 MHz, $CDCl_3$) δ 7.63 – 7.54 (m, 4H), 7.49 – 7.39 (m, 4H), 7.39 – 7.28 (m, 2H), 7.19 (s, 2H).

^{13}C NMR (75 MHz, $CDCl_3$) δ 137.4, 128.8, 128.8, 127.7, 126.6.

***(E)*-1-Methyl-4-styrylbenzene (3b)**

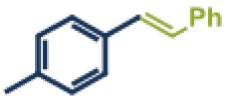

Prepared following the general procedure B from commercially available starting material. The crude product was purified *via* column chromatography (dry load, 12 g silica, hex:EtOAc 50:1) to yield 135 mg (70%) of the title compound as a white solid.

Analytical data is in accordance with literature.[1]

^{1}H NMR (400 MHz, $CDCl_3$) δ 7.59 – 7.52 (m, 2H), 7.47 (d, J = 7.9 Hz, 2H), 7.40 (t, J = 7.6 Hz, 2H), 7.34 – 7.26 (m, 1H), 7.22 (d, J = 7.9 Hz, 2H), 7.13 (d, J = 2.6 Hz, 2H), 2.41 (s, 3H).

^{13}C NMR (101 MHz, $CDCl_3$) δ 137.6, 137.6, 134.7, 129.5, 128.8, 128.7, 127.8, 127.5, 126.6, 126.5, 21.4.

***(E)*-2-Styrlnaphthalene (3c)**

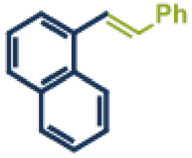

Prepared following the general procedure B from commercially available starting material. The crude product was purified *via* column chromatography (dry load, 12 g silica, hex:EtOAc 100:1) to yield 183 mg (80%) of the title compound as a white solid.

Analytical data is in accordance with literature.[2]

^{1}H NMR (400 MHz, $CDCl_3$) δ 8.20 (d, J = 8.3 Hz, 1H), 7.91 – 7.81 (m, 2H), 7.78 (d, J = 8.3 Hz, 1H), 7.72 (d, J = 7.3 Hz, 1H), 7.58 (dd, J = 7.7, 1.7 Hz, 2H), 7.50 (ddt, J = 13.8, 12.4, 4.5 Hz, 3H), 7.38 (t, J = 7.6 Hz, 2H), 7.33 – 7.24 (m, 1H), 7.13 (d, J = 16.0 Hz, 1H).

^{13}C NMR (101 MHz, $CDCl_3$) δ 137.8, 135.2, 133.9, 131.9, 131.6, 128.9, 128.8, 128.2, 127.9, 126.8, 126.2, 126.0, 125.8, 123.9, 123.8.

***(E)*-1-Methoxy-4-styrylbenzene (3d)**

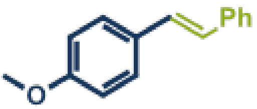

Prepared following the general procedure B from commercially available starting material. The crude product was purified *via* column chromatography (dry load, 12 g silica, hex:EtOAc 50:1) to yield 166 mg (79%) of the title compound as a white solid.

Analytical data is in accordance with literature.[1]

^{1}H NMR (400 MHz, $CDCl_3$) δ 7.44 – 7.33 (m, 4H), 7.26 (t, J = 7.7 Hz, 2H), 7.18 – 7.11 (m, 1H), 6.98 (d, J = 16.2 Hz, 1H), 6.89 (d, J = 16.4 Hz, 1H), 6.85 – 6.78 (m, 2H), 3.74 (s, 3H).

^{13}C NMR (101 MHz, $CDCl_3$) δ 159.4, 137.8, 130.3, 128.8, 128.3, 127.9, 127.3, 126.8, 126.4, 114.2, 55.4.

***(E)*-1-Methoxy-2-styrylbenzene (3e)**

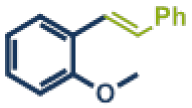

Prepared following the general procedure B from commercially available starting material. The crude product was purified *via* column chromatography (dry load, 12 g silica, hex:EtOAc 50:1, 20:1) to yield 153 mg (73%) of the title compound as a white solid.

Analytical data is in accordance with literature.[3]

^{1}H NMR (300 MHz, $CDCl_3$) δ 7.62 (dd, *J* = 7.7, 1.8 Hz, 1H), 7.60 – 7.44 (m, 3H), 7.42 – 7.33 (m, 2H), 7.27 (tdd, *J* = 7.4, 4.8, 2.3 Hz, 2H), 7.14 (d, *J* = 16.5 Hz, 1H), 6.99 (td, *J* = 7.5, 1.1 Hz, 1H), 6.92 (dt, *J* = 8.3, 2.1 Hz, 1H), 3.89 (s, 3H).

^{13}C NMR (75 MHz, $CDCl_3$) δ 157.0, 138.1, 129.2, 128.8, 128.70, 127.5, 126.7, 126.55, 126.52, 123.6, 120.9, 111.0, 55.6.

***(E)*-1-Methoxy-3-styrylbenzene (3f)**

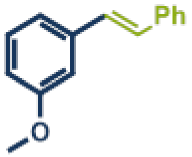

Prepared following the general procedure B from commercially available starting material. The crude product was purified *via* column chromatography (dry load, 12 g silica, hex:EtOAc 50:1, 20:1) to yield, 147 mg (70%) of the title compound as a white solid.

Analytical data is in accordance with literature.[3]

^{1}H NMR (400 MHz, $CDCl_3$) δ 7.56 – 7.49 (m, 2H), 7.41 – 7.34 (m, 2H), 7.34 – 7.26 (m, 2H), 7.16 – 7.08 (m, 3H), 7.06 (dd, *J* = 2.5, 1.6 Hz, 1H), 6.83 (ddd, *J* = 8.2, 2.6, 1.0 Hz, 1H), 3.86 (s, 3H).

^{13}C NMR (101 MHz, $CDCl_3$) δ 160.1, 138.9, 137.4, 129.8, 129.2, 128.8, 128.7, 127.8, 126.7, 119.4, 113.5, 111.9, 55.4.

***(E)*-1-Nitro-4-styrylbenzene (3g)** *from 4-nitro-1-iodobenzene*

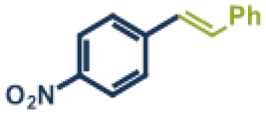

Prepared following the general procedure B from commercially available starting material. The crude product was purified *via* column chromatography (dry load, 12 g silica, hex:EtOAc 50:1, 20:1) to yield 137 mg (61%) of the title compound as a yellow solid.

Analytical data is in accordance with literature.[3]

^{1}H NMR (300 MHz, $CDCl_3$) δ 8.21 – 8.00 (m, 2H), 7.61 – 7.50 (m, 2H), 7.50 – 7.41 (m, 2H), 7.37 – 7.23 (m, 3H), 7.23 – 7.12 (m, 1H), 7.05 (d, *J* = 16.3 Hz, 1H).

^{13}C NMR (75 MHz, $CDCl_3$) δ 146.9, 144.0, 136.3, 133.4, 129.02, 128.97, 127.2, 127.0, 126.4, 124.3.

***(E)*-Methyl 4-styrylbenzoate (3h)**

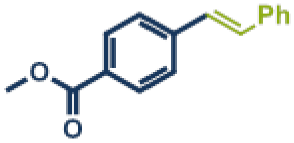

Prepared following the general procedure B from commercially available starting material. The crude product was purified *via* column chromatography (dry load, 12 g silica, hex:EtOAc 20:1) to yield 137 mg (58%) of the title compound as a waxy solid.

Analytical data is in accordance with literature.[4]

^{1}H NMR (400 MHz, $CDCl_3$) δ 8.07 – 8.00 (m, 2H), 7.60 – 7.50 (m, 4H), 7.38 (dd, *J* = 8.5, 6.8 Hz, 2H), 7.35 – 7.18 (m, 2H), 7.13 (d, *J* = 16.3 Hz, 1H), 3.93 (s, 3H).

^{13}C NMR (101 MHz, $CDCl_3$) δ 167.0, 141.9, 136.9, 131.3, 130.1, 129.0, 128.9, 128.4, 127.7, 126.9, 126.4, 52.2.

***(E)*-4-Styrylbenzaldehyde (3i)**

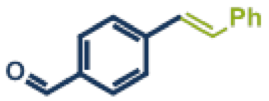

Prepared following the general procedure B from commercially available starting material. The crude product was purified *via* column chromatography (dry load, 12 g silica, hex:EtOAc 50:1) to yield 100 mg (48%) of the title compound as a yellow solid.

Analytical data is in accordance with literature.[5]

^{1}H NMR (300 MHz, $CDCl_3$) δ 9.92 (s, 1H), 7.80 (d, *J* = 8.3 Hz, 2H), 7.58 (d, *J* = 8.3 Hz, 2H), 7.53 – 7.41 (m, 2H), 7.38 – 7.14 (m, 5H), 7.07 (d, *J* = 16.3 Hz, 1H).

^{13}C NMR (75 MHz, $CDCl_3$) δ 191.8, 143.6, 136.7, 135.5, 132.3, 130.4, 128.9, 128.7, 127.5, 127.0.

***(E)*-1-Fluoro-4-styrylbenzene (3j)**

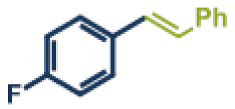

Prepared following the general procedure B from commercially available starting material. The crude product was purified *via* column chromatography (dry load, 12 g silica, hex:EtOAc 50:1) to yield 132 mg (67%) of the title compound as slightly yellow oil.

Analytical data is in accordance with literature.[1, 6]

^{1}H NMR (300 MHz, $CDCl_3$) δ 7.59 – 7.41 (m, 4H), 7.41 – 7.31 (m, 2H), 7.30 – 7.22 (m, 1H), 7.13 – 6.96 (m, 4H).

^{13}C NMR (75 MHz, $CDCl_3$) δ 162.5 (d, *J* = 247.1 Hz), 137.3, 133.7 (d, *J* = 3.3 Hz), 128.8, 128.6 (d, *J* = 2.5 Hz), 128.1 (d, *J* = 8.0 Hz), 127.8, 127.6, 126.6, 115.8 (d, *J* = 21.7 Hz).

^{19}F NMR (376 MHz, $CDCl_3$) δ -114.20.

***(E)*-1-Bromo-4-styrylbenzene (3k)**

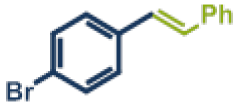

Prepared following the general procedure B from commercially available starting material. The crude product was purified *via* column chromatography (dry load, 12 g silica, *n*-hexane) to yield 181 mg (70%) of the title compound as off-white solid.

Analytical data is in accordance with literature.[6]

^{1}H NMR (400 MHz, $CDCl_3$) δ 7.51 (dd, *J* = 11.0, 8.0 Hz, 4H), 7.39 (dt, *J* = 7.9, 3.3 Hz, 4H), 7.34 – 7.27 (m, 1H), 7.11 (d, *J* = 16.4 Hz, 1H), 7.04 (d, *J* = 16.4 Hz, 1H).

^{13}C NMR (101 MHz, $CDCl_3$) δ 137.1, 136.4, 131.9, 129.5, 128.9, 128.1, 128.0, 127.5, 126.7, 121.4.

***(E)*-1-Cyano-4-styryl-benzene (3l)**

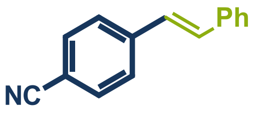

Prepared following the general procedure B from commercially available starting material. The crude product was purified *via* column chromatography (dry load, 12 g silica, hex:EtOAc 20:1) to yield 139 mg (68%) of the title compound as a white solid.

Analytical data is in accordance with literature.[4]

^{1}H NMR (400 MHz, $CDCl_3$) δ 7.63 (d, *J* = 8.4 Hz, 2H), 7.60 – 7.51 (m, 4H), 7.40 (td, *J* = 7.2, 1.5 Hz, 2H), 7.36 – 7.28 (m, 1H), 7.21 (d, *J* = 16.3 Hz, 1H), 7.09 (d, *J* = 16.4 Hz, 1H).

^{13}C NMR (101 MHz, $CDCl_3$) δ 141.9, 136.4, 132.6, 132.5, 129.0, 128.7, 127.0, 126.9, 126.8, 119.1, 110.6.

Tert-Butyl _(E)_-3-(4-bromophenyl)acrylate (3m)

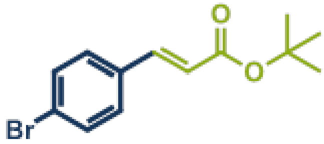

Prepared following the general procedure B from commercially available starting material. The crude product was purified *via* column chromatography (dry load, 12 g silica, hex:EtOAc 50:1) to yield 183 mg (65%) of the title compound as a white solid.

Analytical data is in accordance with literature.[7]

^{1}H NMR (400 MHz, $CDCl_3$) δ 7.46 – 7.36 (m, 3H), 7.30 – 7.23 (m, 2H), 6.26 (d, *J* = 16.0 Hz, 1H), 1.44 (s, 9H).

^{13}C NMR (101 MHz, $CDCl_3$) δ 166.0, 142.2, 133.7, 132.1, 129.4, 124.2, 121.0, 80.7, 28.3.

_(E)_-1-Phenoxy-4-styrylbenzene (3o)

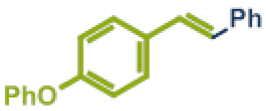

Prepared following the general procedure B from commercially available starting material. The crude product was purified *via* column chromatography (dry load, 12 g silica, hex:EtOAc 50:1) to yield 143 mg (53%) of the title compound as a white solid.

Analytical data is in accordance with literature.[8]

^{1}H NMR (400 MHz, $CDCl_3$) δ 7.54 (tt, *J* = 9.5, 2.2 Hz, 4H), 7.45 – 7.35 (m, 4H), 7.35 – 7.27 (m, 1H), 7.20 – 7.13 (m, 1H), 7.13 – 7.02 (m, 6H).

^{13}C NMR (101 MHz, $CDCl_3$) δ 157.2, 157.0, 137.5, 132.7, 129.9, 128.8, 128.00, 127.98, 127.9, 127.6, 126.5, 123.5, 119.11, 119.10.

_(E)_-4-Phenylstilbene (3p)

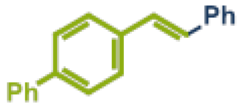

Prepared following the general procedure B from commercially available starting material. The crude product was purified *via* column chromatography (dry load, 12 g silica, *n*-hexanes) to yield 180 mg (70%) of the title compound as white solid.

Analytical data is in accordance with literature.[6]

^{1}H NMR (400 MHz, $CDCl_3$) δ 7.65 – 7.55 (m, 6H), 7.55 – 7.50 (m, 2H), 7.44 (dd, *J* = 8.4, 6.9 Hz, 2H), 7.40 – 7.35 (m, 2H), 7.35 – 7.22 (m, 3H), 7.15 (s, 2H).

^{13}C NMR (101 MHz, $CDCl_3$) δ 140.8, 140.5, 137.5, 136.5, 129.0, 128.9, 128.8, 128.3, 127.8, 127.50, 127.47, 127.08, 127.07, 126.7.

_(E)_-2-Styrylnaphthalene (3q)

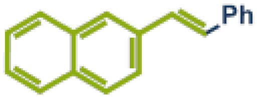

Prepared following the general procedure B from commercially available starting material. The crude product was purified *via* column chromatography (dry load, 12 g silica, hex:EtOAc 20:1) to yield 114 mg (50%) of the title compound as white crystals.

Analytical data is in accordance with literature.[6]

^1H NMR (400 MHz, $CDCl_3$) δ 7.91 – 7.80 (m, 4H), 7.77 (dd, *J* = 8.6, 1.8 Hz, 1H), 7.63 – 7.56 (m, 2H), 7.54 – 7.45 (m, 2H), 7.41 (dd, *J* = 8.4, 6.9 Hz, 2H), 7.37 – 7.22 (m, 3H).

^{13}C NMR (101 MHz, CDCl3) δ 137.5, 135.0, 133.8, 133.2, 129.2, 128.9, 128.9, 128.4, 128.1, 127.8, 127.8, 126.8, 126.7, 126.5, 126.0, 123.7.

***(E)*-1-Nitro-4-styrylbenzene (3g)** *from 4-nitrovinyl benzene*

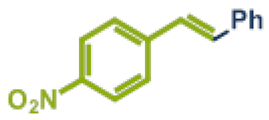

Prepared following the general procedure B from commercially available starting material. The crude product was purified *via* column chromatography (dry load, 12 g silica, hex:EtOAc 50:1) to yield 149 mg (66%) of the title compound as a yellow solid.

Analytical data is in accordance with previous reaction products and with literature.[3]

***(E)*-4-Styrylpyridine (3r)**

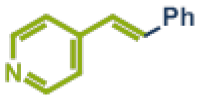

Prepared following the general procedure B from commercially available starting material. The crude product was purified *via* column chromatography (dry load, 12 g silica, hex:EtOAc 20:1, 10:1) to yield 127 mg (70%) of the title compound as white solid.

Analytical data is in accordance with literature.[9]

^1H NMR (400 MHz, $CDCl_3$) δ 8.57 (d, *J* = 6.3 Hz, 2H), 7.53 (d, *J* = 7.2 Hz, 2H), 7.38 (t, *J* = 7.4 Hz, 2H), 7.35 – 7.24 (m, 4H), 6.99 (d, *J* = 16.3 Hz, 1H).

^{13}C NMR (101 MHz, $CDCl_3$) δ 150.2, 144.6, 136.2, 133.2, 128.9, 128.8, 127.1, 126.0, 120.9.

***(E)*-1-Styryl-4-(trifluoromethyl)benzene (3s)**

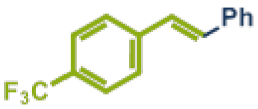

Prepared following the general procedure B from commercially available starting material. The crude product was purified *via* column chromatography (dry load, 12 g silica, *n*-hexanes) to yield 193 mg (78%) of the title compound as colorless oil.

Analytical data is in accordance with literature.[4]

^1H NMR (400 MHz, $CDCl_3$) δ 7.61 (s, 4H), 7.58 – 7.51 (m, 2H), 7.39 (td, *J* = 7.1, 1.3 Hz, 2H), 7.35 – 7.27 (m, 1H), 7.16 (q, *J* = 16.3 Hz, 2H).

^{13}C NMR (101 MHz, $CDCl_3$) δ 141.0, 136.8, 131.4, 129.4 (d, *J* = 32.1 Hz), 129.0, 128.4, 127.3, 126.9, 126.7, 125.8 (q, *J* = 3.6 Hz), 121.7 (q, *J* = 272 Hz).

***(E)*-3,4-Dimethoxystilbene (3t)**

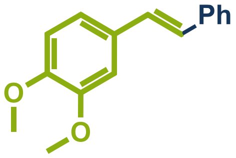

Prepared following the general procedure B from commercially available starting material. The crude product was purified *via* column chromatography (dry load, 12 g silica, hex:EtOAc 20:1) to yield 44 mg (18%) of the title compound as white crystals.

Analytical data is in accordance with literature.[6]

^1H NMR (400 MHz, $CDCl_3$) δ 7.54 – 7.47 (m, 2H), 7.40 – 7.31 (m, 2H), 7.28 – 7.22 (m, 1H), 7.11 – 7.01 (m, 3H), 6.98 (d, *J* = 16.3 Hz, 1H), 6.87 (d, *J* = 8.2 Hz, 1H), 3.96 (s, 3H), 3.91 (s, 3H).

^{13}C NMR (101 MHz, $CDCl_3$) δ 149.3, 149.1, 137.7, 130.6, 128.8, 128.6, 127.4, 126.9, 126.4, 120.0, 111.4, 108.9, 56.1, 56.0.

Tert-Butyl cinnamate (3u)

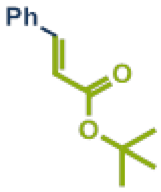

Prepared following the general procedure B from commercially available starting material to yield 168 mg (82%) of the title compound as slightly yellow oil.

Analytical data is in accordance with literature.[7]

^{1}H NMR (400 MHz, $CDCl_3$) δ 7.59 (d, J = 16.0 Hz, 1H), 7.54 – 7.46 (m, 2H), 7.42 – 7.32 (m, 3H), 6.37 (d, J = 16.0 Hz, 1H), 1.54 (s, 9H).

^{13}C NMR (101 MHz, $CDCl_3$) δ 166.5, 143.7, 134.8, 130.1, 128.9, 128.1, 120.3, 80.6, 28.4.

_N_-Morpholinocinnamide (3v)

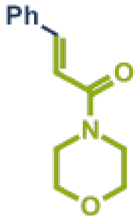

Prepared following the general procedure B from commercially available starting material to yield 243 mg (99%) of the title compound as slightly yellow oil.

Analytical data is in accordance with literature.[10]

^{1}H NMR (400 MHz, $CDCl_3$) δ 7.68 (d, J = 15.4 Hz, 1H), 7.50 (dd, J = 7.2, 2.3 Hz, 2H), 7.40 – 7.28 (m, 3H), 6.83 (d, J = 15.4 Hz, 1H), 3.77 – 3.59 (m, 8H).

^{13}C NMR (101 MHz, $CDCl_3$) δ 165.6, 143.2, 135.2, 129.8, 128.9, 127.8, 116.6, 66.9, 46.3, 42.5.

Hexylcinnamate (3w)

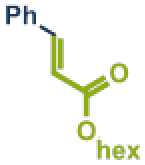

Prepared following the general procedure B from commercially available starting material to yield 195 mg (84%) of the title compound as slightly yellow oil.

Analytical data is in accordance with literature.[11]

^{1}H NMR (400 MHz, $CDCl_3$) δ 7.69 (d, J = 16.0 Hz, 1H), 7.56 – 7.48 (m, 2H), 7.38 (p, J = 3.4 Hz, 3H), 6.45 (d, J = 16.0 Hz, 1H), 4.20 (t, J = 6.8 Hz, 2H), 1.70 (p, J = 6.9 Hz, 2H), 1.44 – 1.30 (m, 6H), 0.95 – 0.86 (m, 3H).

^{13}C NMR (101 MHz, $CDCl_3$) δ 167.2, 144.7, 134.6, 130.3, 129.0, 128.2, 118.4, 64.8, 31.6, 28.8, 25.8, 22.7, 14.1.

_(E)_-Stilbene (3a) _from benzaldehyde_

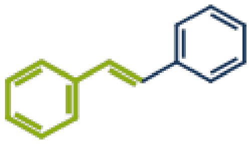

Prepared following the general procedure C from commercially available starting material. The crude product was purified _via_ column chromatography (dry load, 12 g silica, _n_-hexanes) to yield 120 mg (67%) of the title compound as white crystals.

Analytical data is in accordance with previous experiments.

_(E)_-1-Chloro-4-styrylbenzene (3x) _from 4-chlorobenzaldehyde_

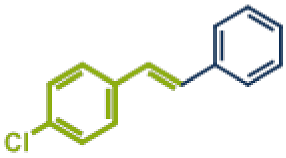

Prepared following the general procedure C from commercially available starting material. The crude product was purified _via_ column chromatography (dry load, 12 g silica, _n_-hexanes) to yield 116 mg (54%) of the title compound as white crystals.

Analytical data is in accordance with literature.[6]

^{1}H NMR (400 MHz, $CDCl_3$) δ 7.57 – 7.50 (m, 2H), 7.49 – 7.43 (m, 2H), 7.43 – 7.28 (m, 5H), 7.09 (d, *J* = 2.9 Hz, 2H).

^{13}C NMR (101 MHz, $CDCl_3$) δ 137.1, 135.9, 133.3, 129.4, 129.0, 128.89, 128.0, 127.8, 127.5, 126.7.

5. NMR Spectra

(E)-1,2-Diphenylethene (**3a**)

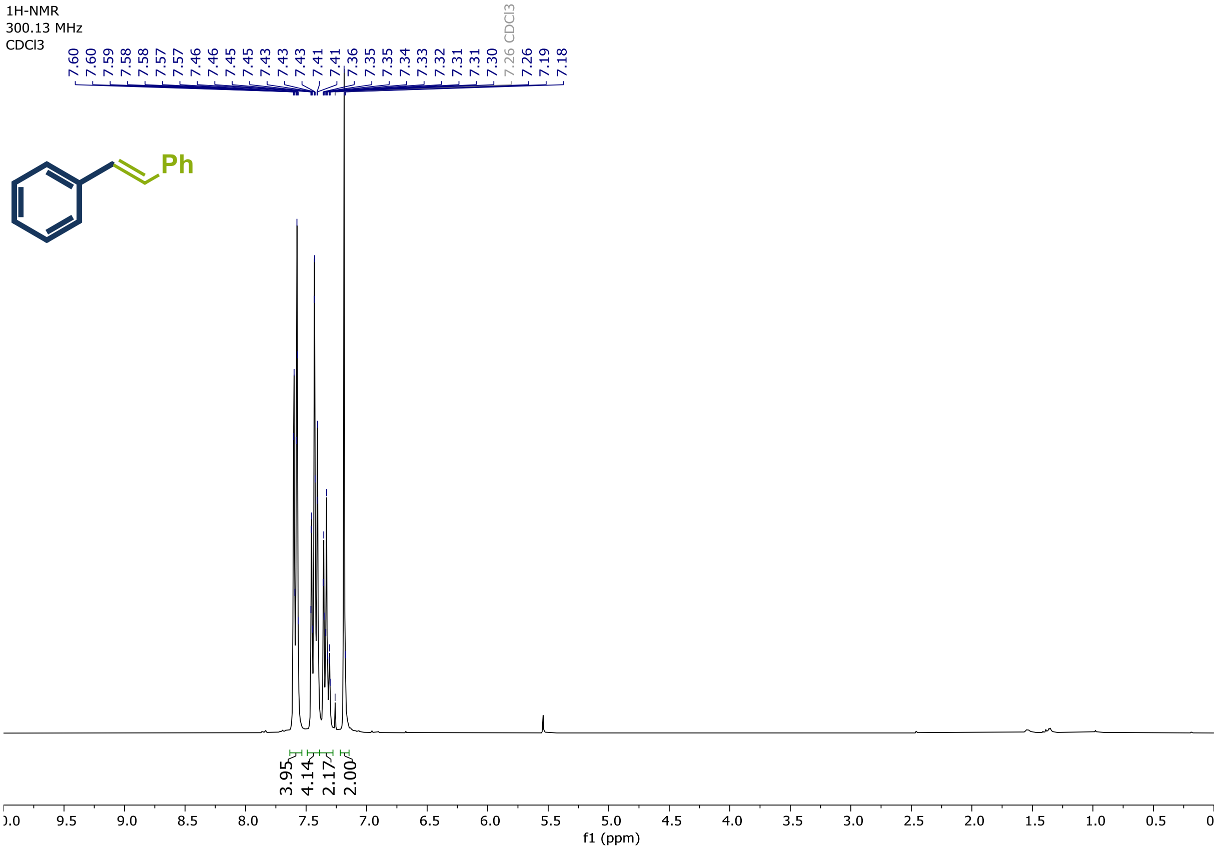

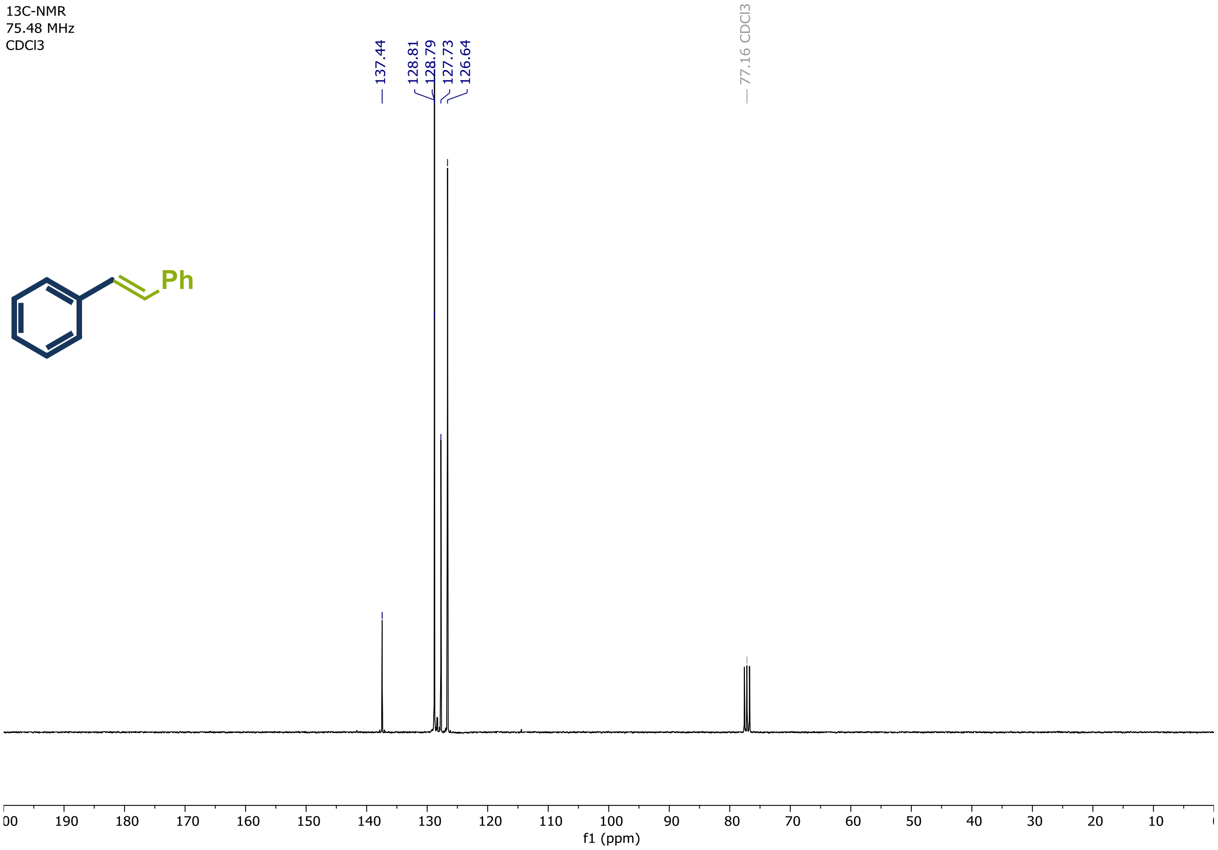

(E)-1-Methyl-4-styrylbenzene (**3b**)

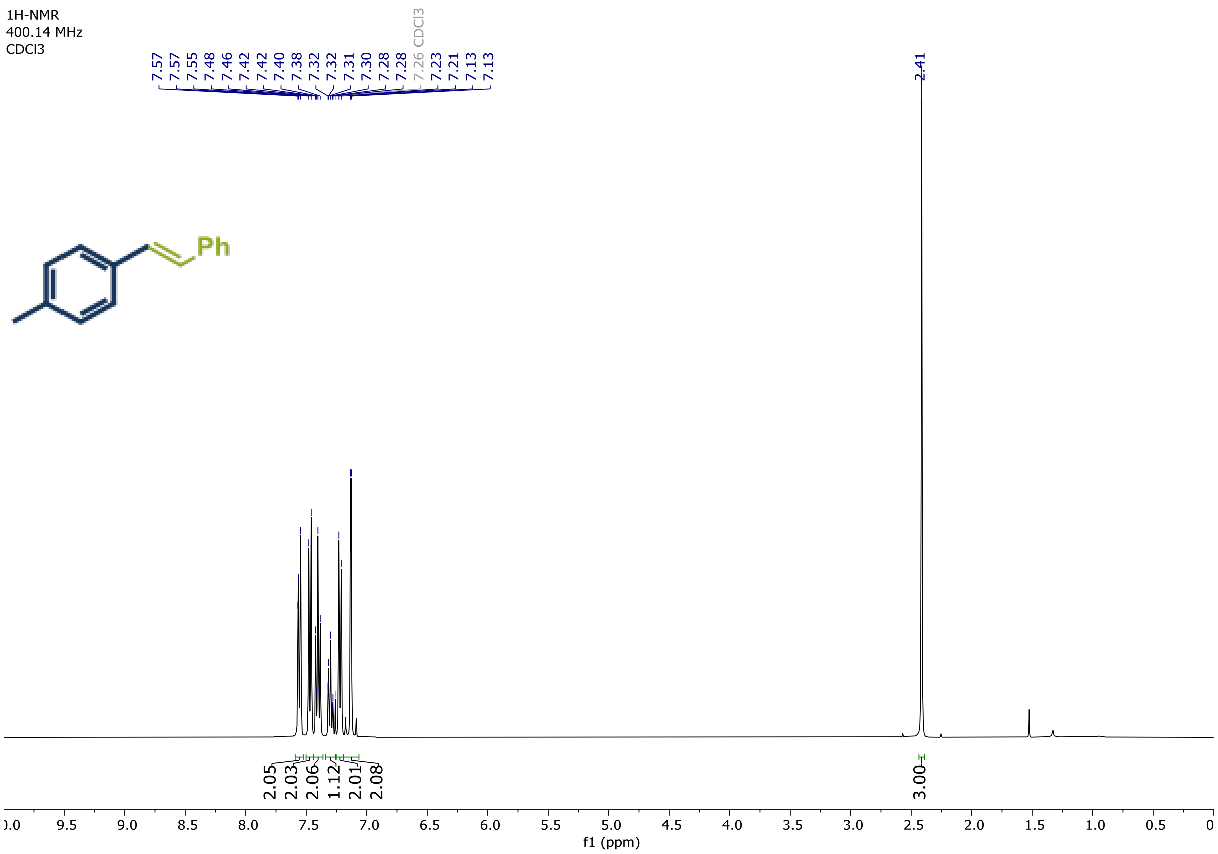

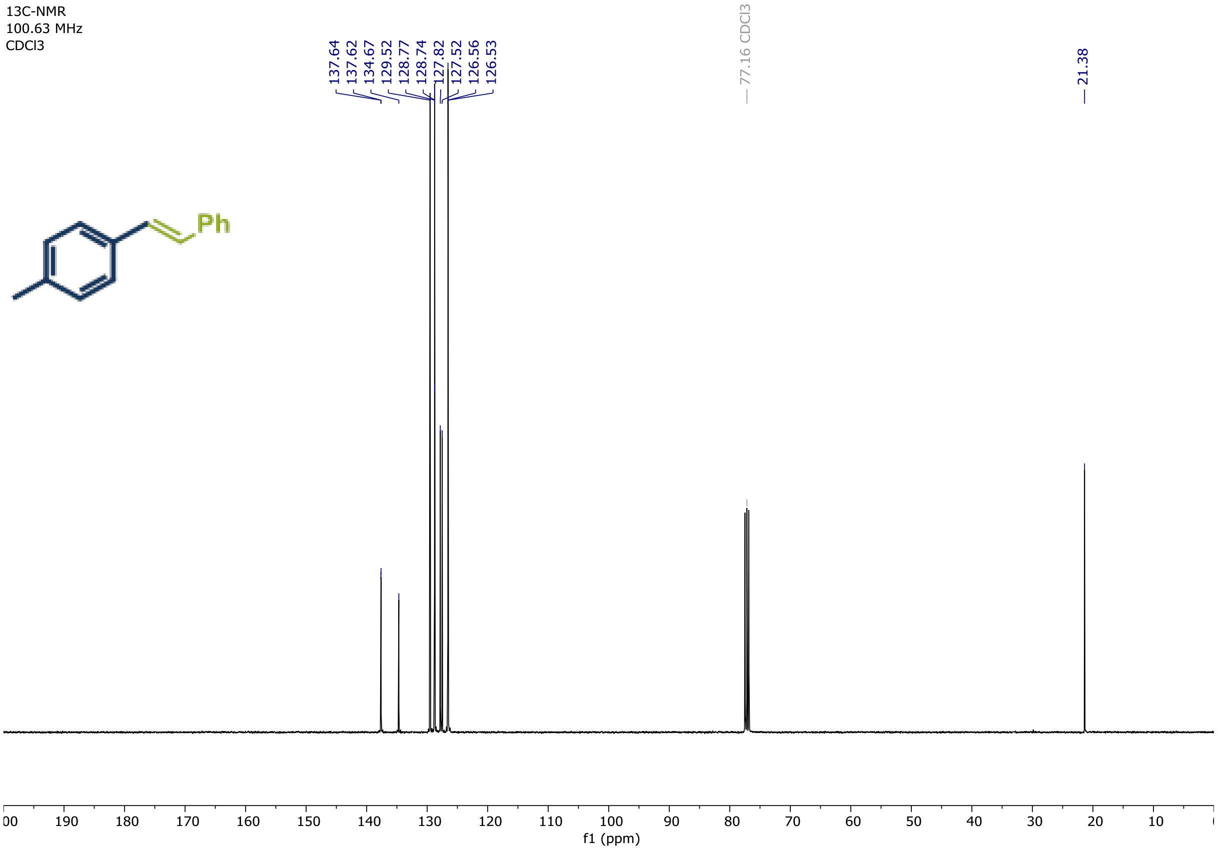

(E)-2-Styrlnaphthalene (**3c**)

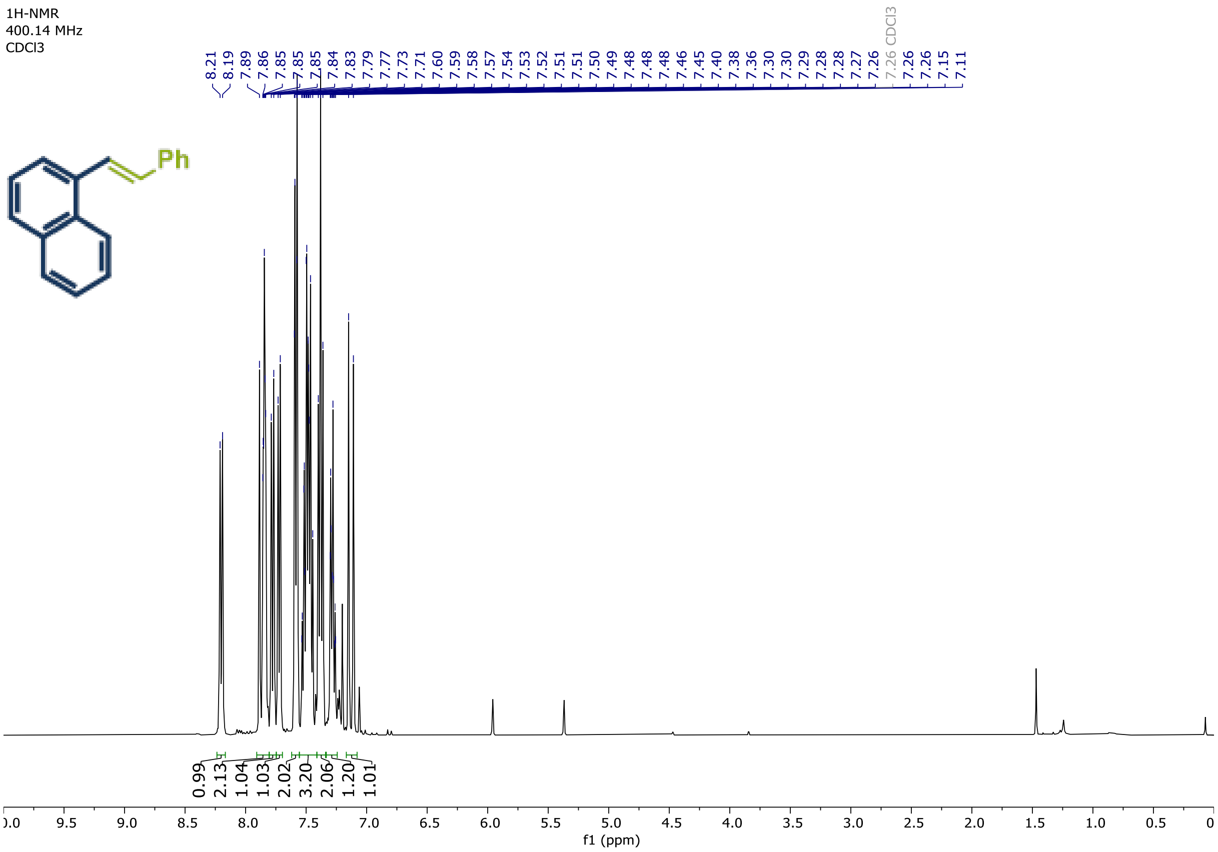

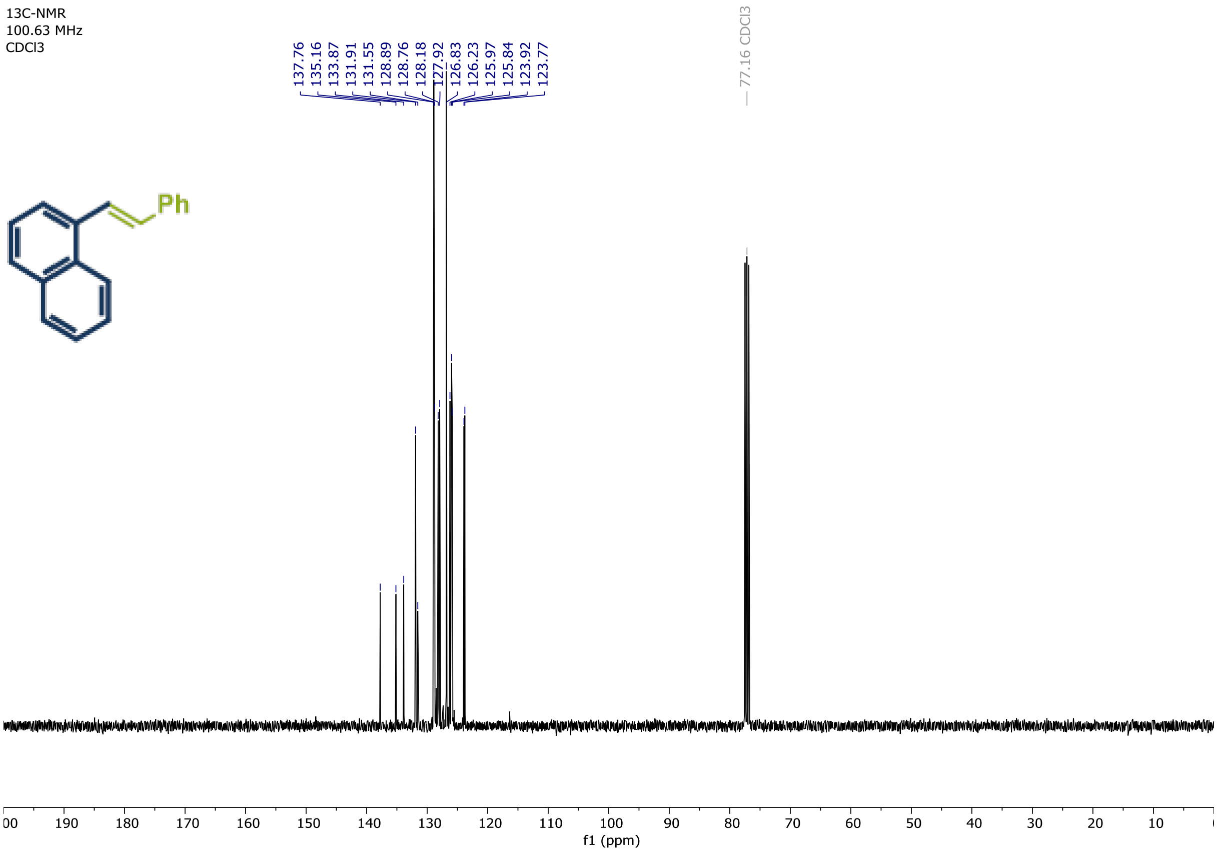

(E)-1-Methoxy-4-styrylbenzene (**3d**)

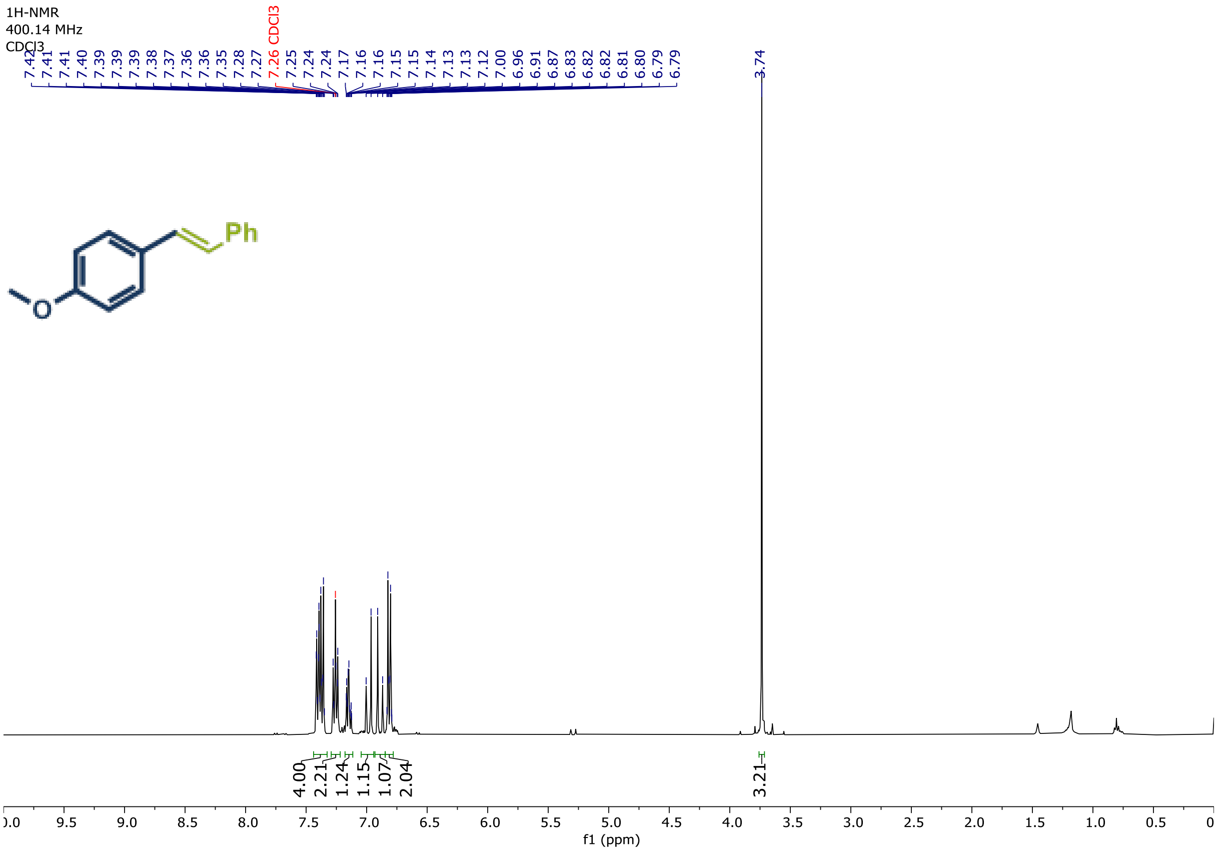

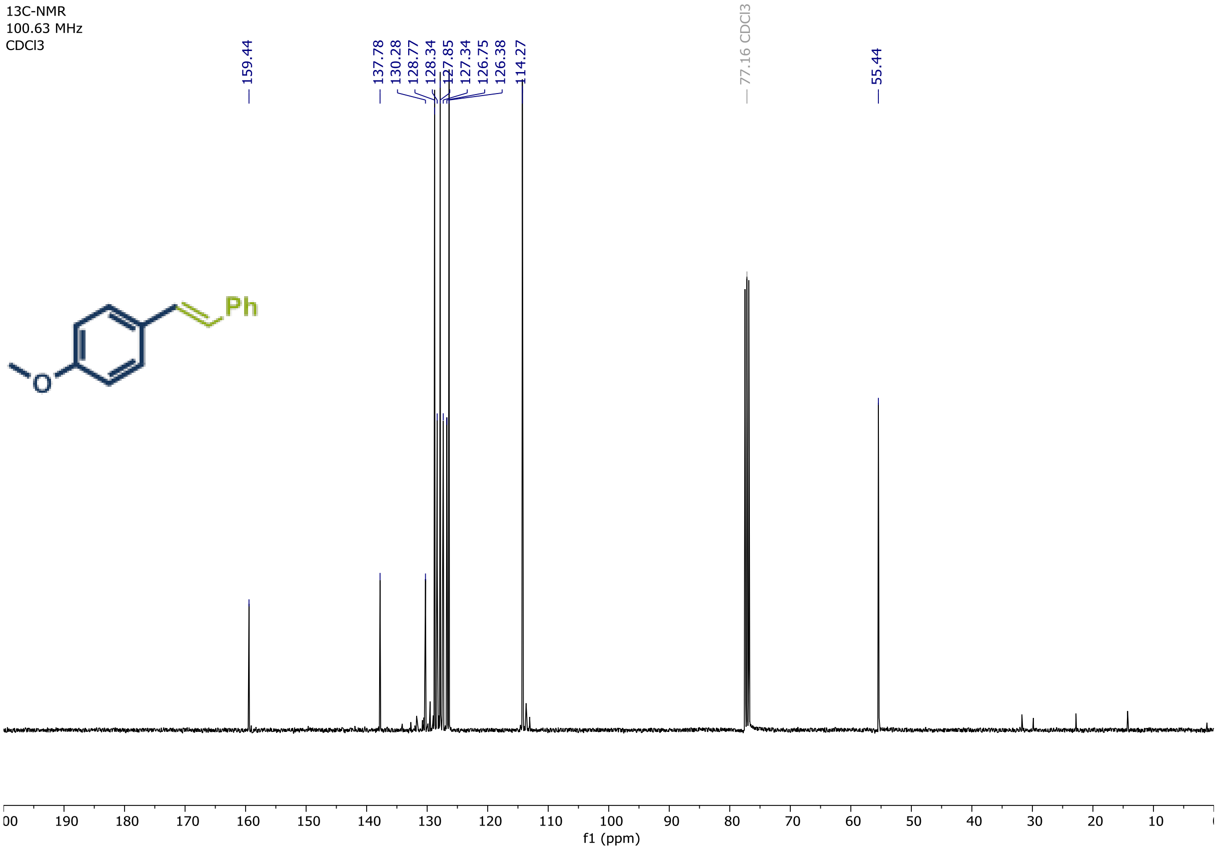

(E)-1-Methoxy-2-styrylbenzene (**3e**)

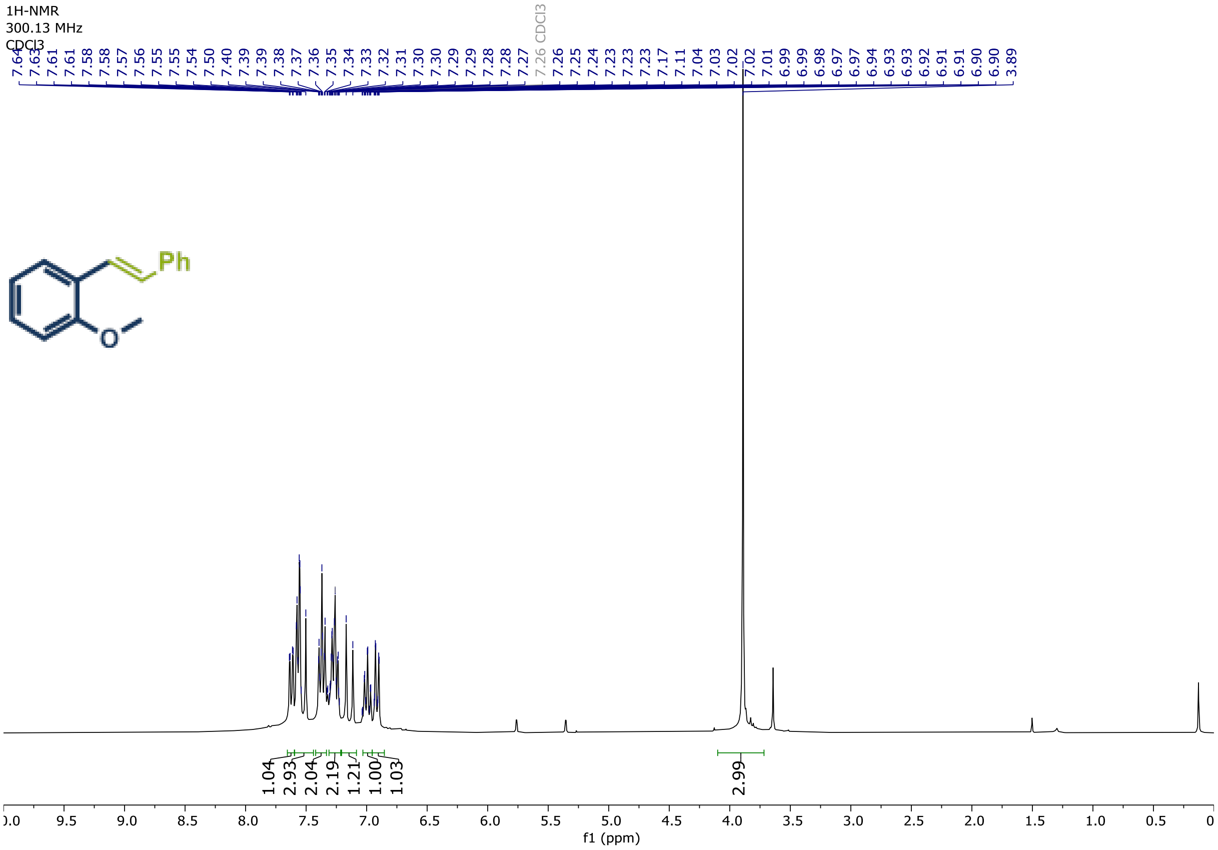

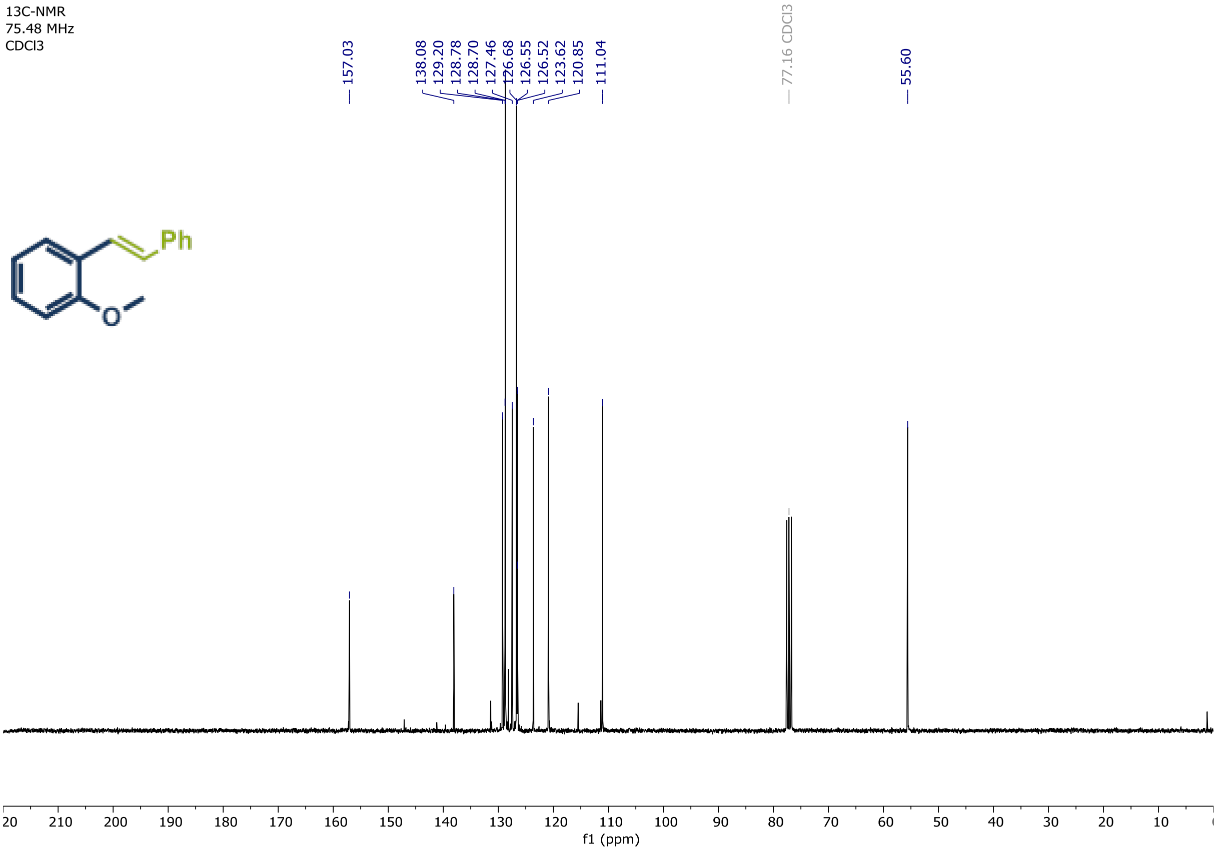

(E)-1-Methoxy-3-styrylbenzene (**3f**)

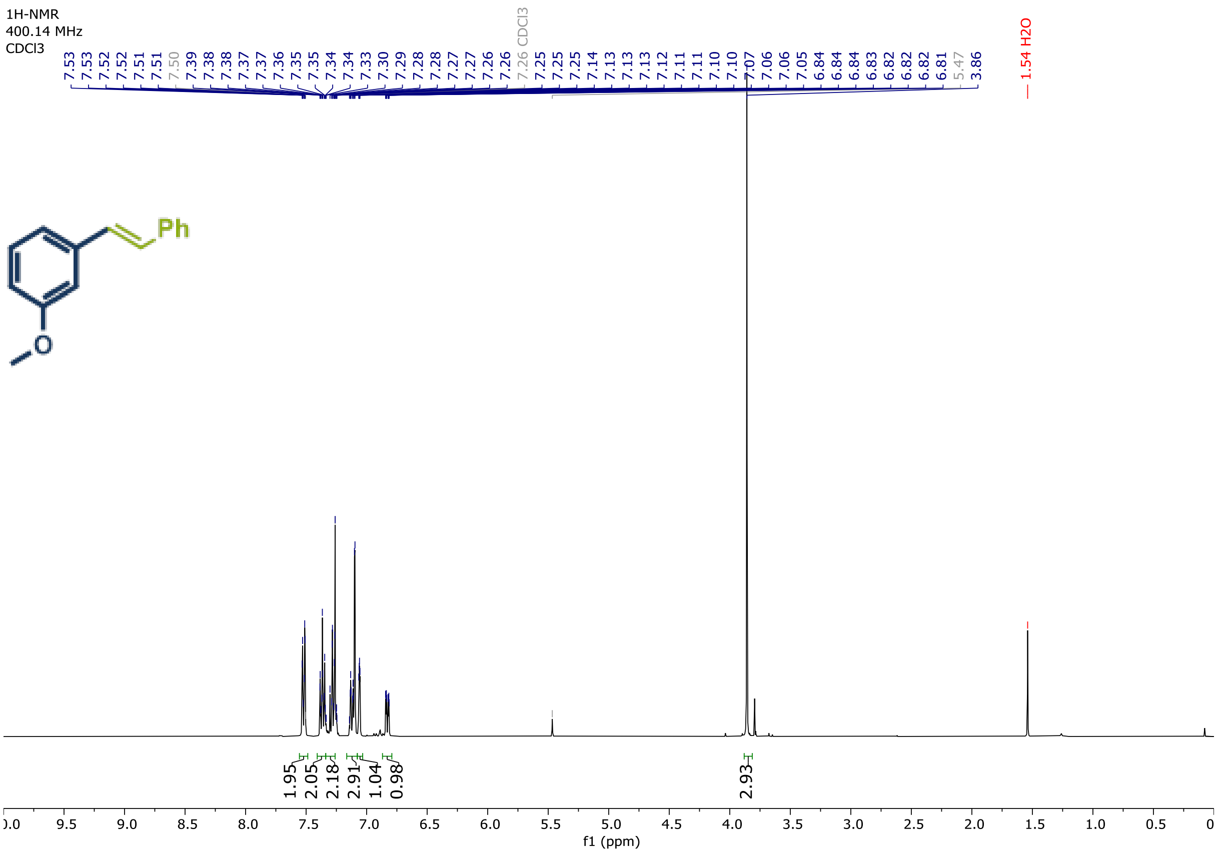

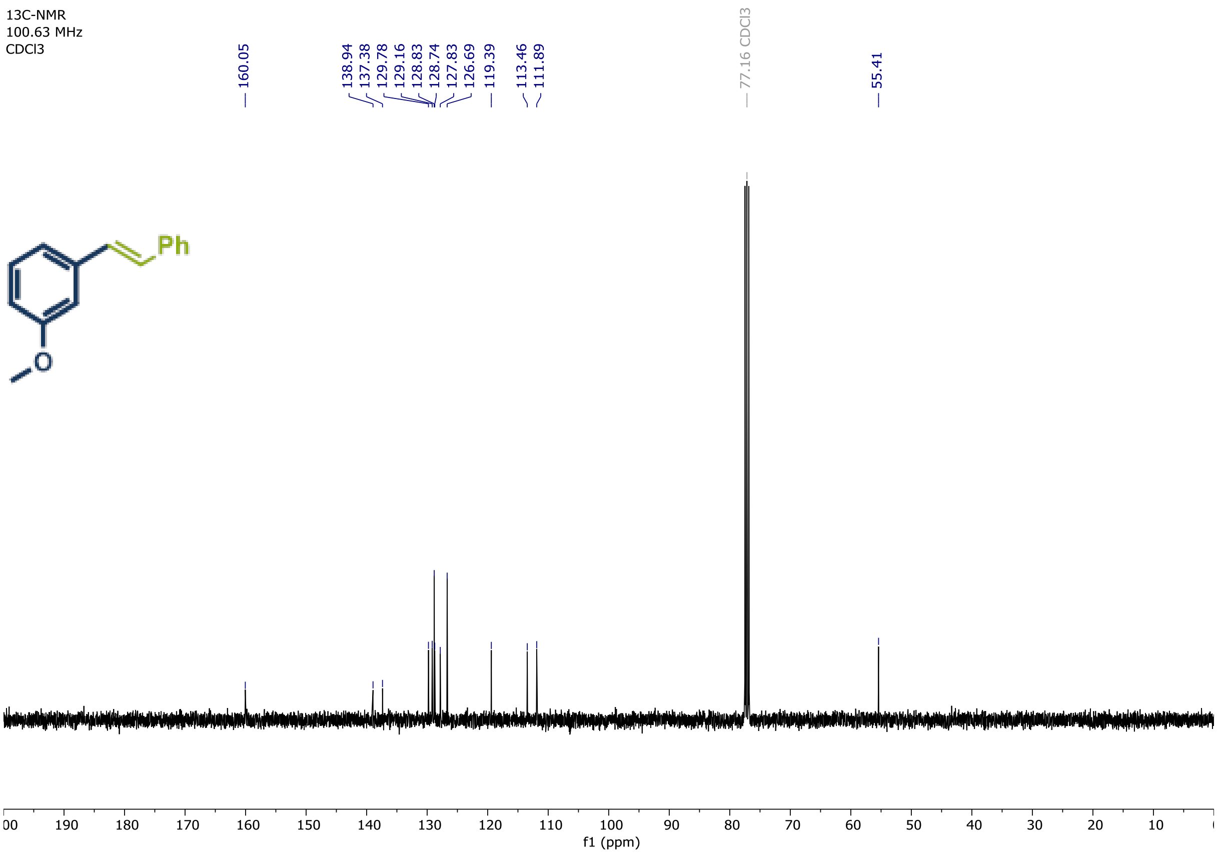

(E)-1-Nitro-4-styrylbenzene (**3g**)

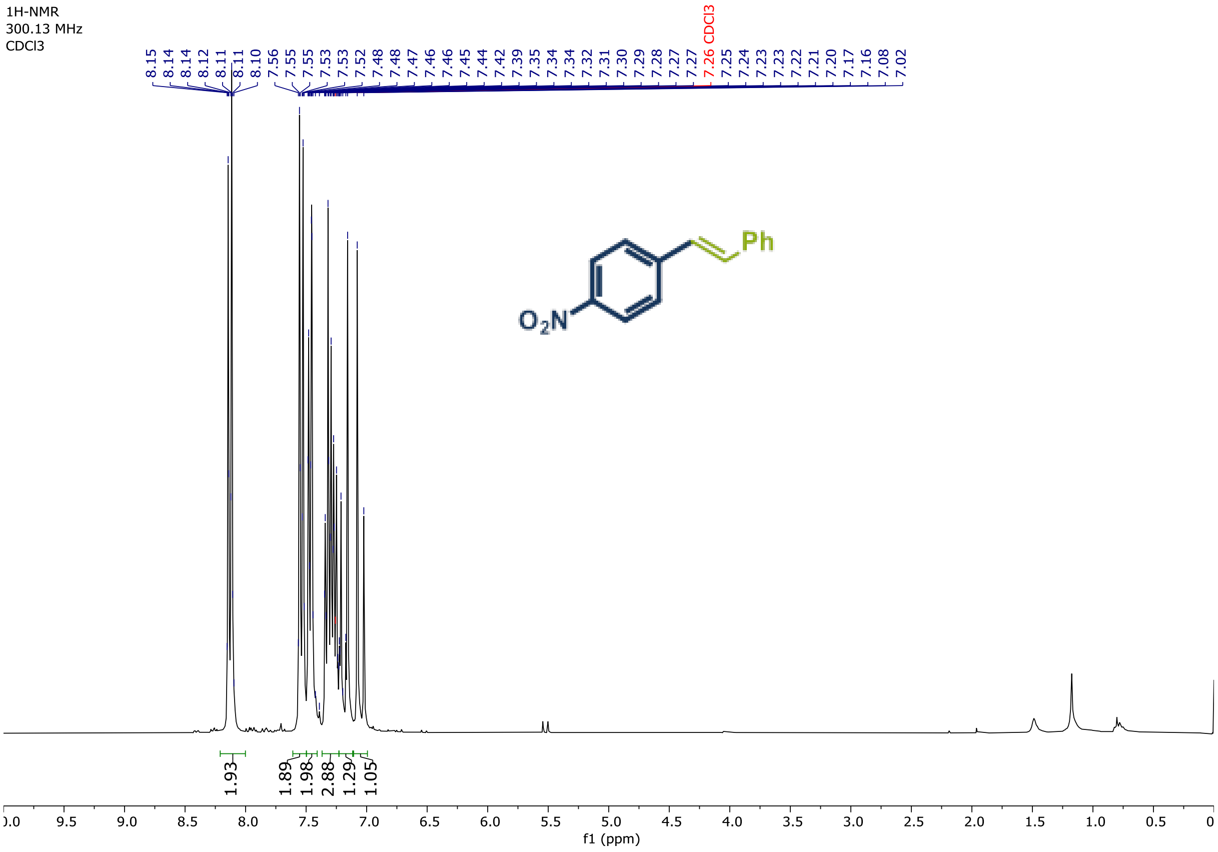

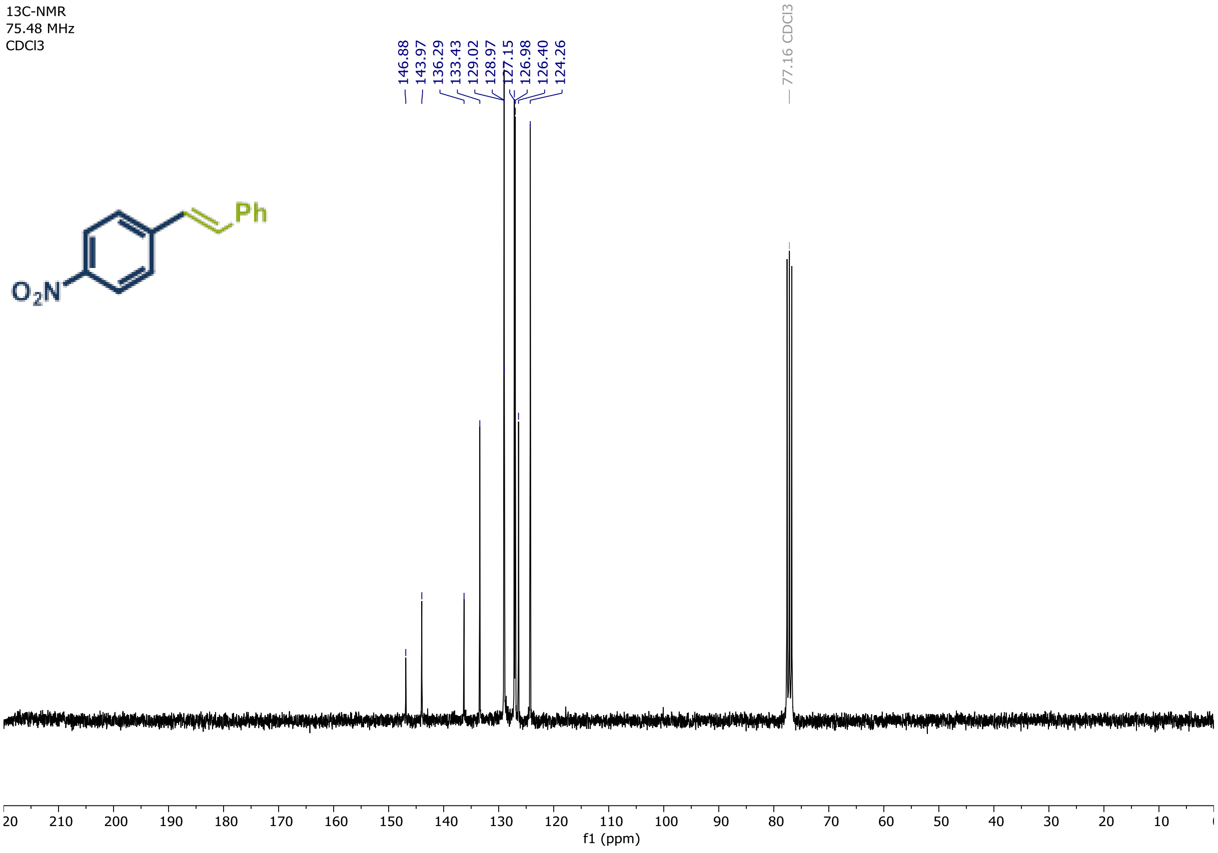

(*E*)-methyl 4-styrylbenzoate (**3h**)

1H-NMR
400.14 MHz
CDCl3

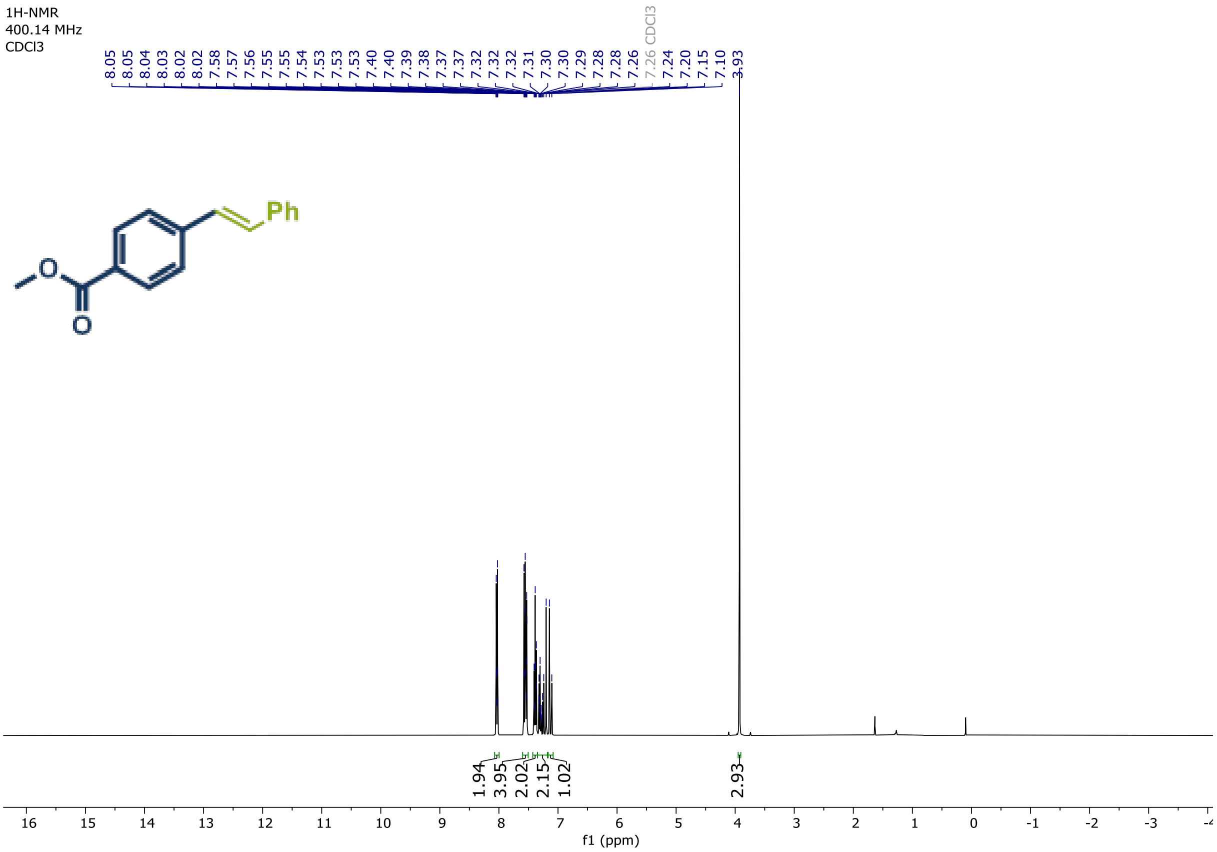

13C-NMR
100.63 MHz
CDCl3

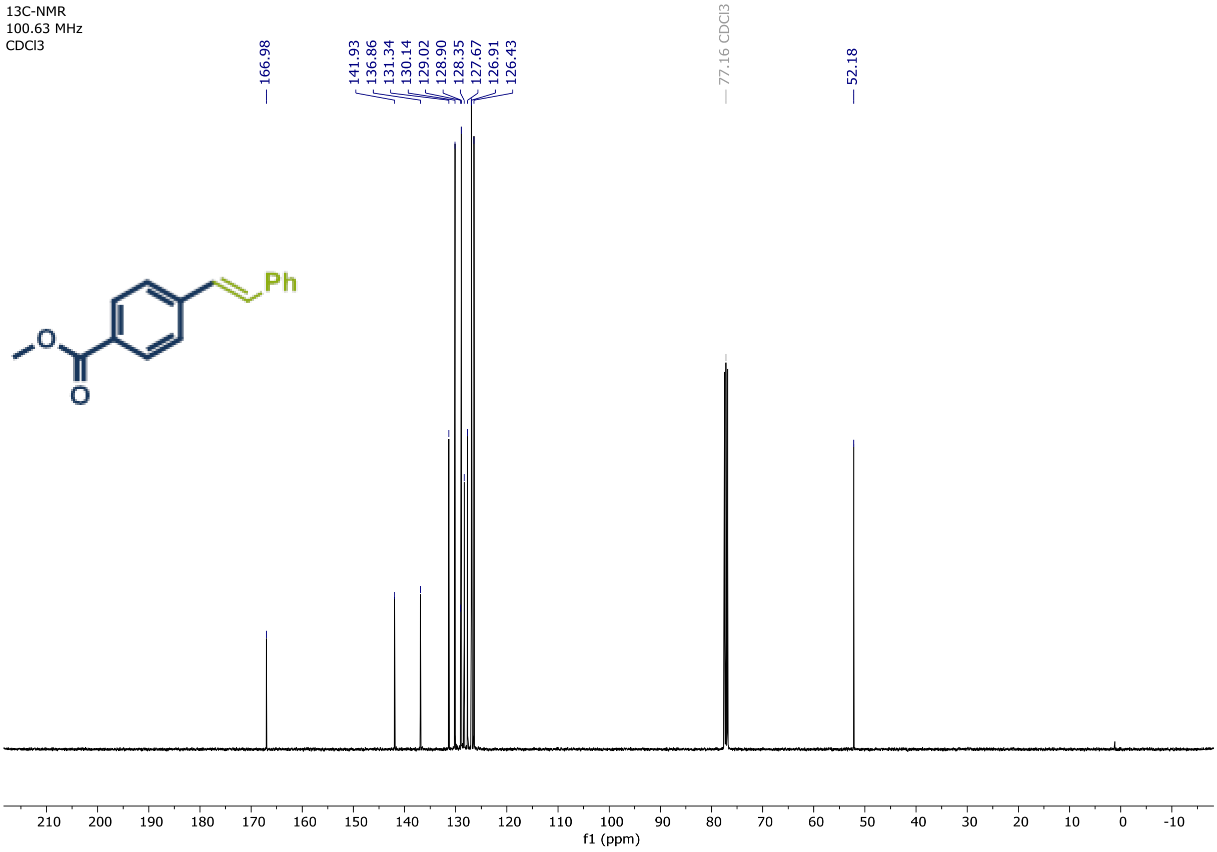

(E)-4-Styrylbenzaldehyde (**3i**)

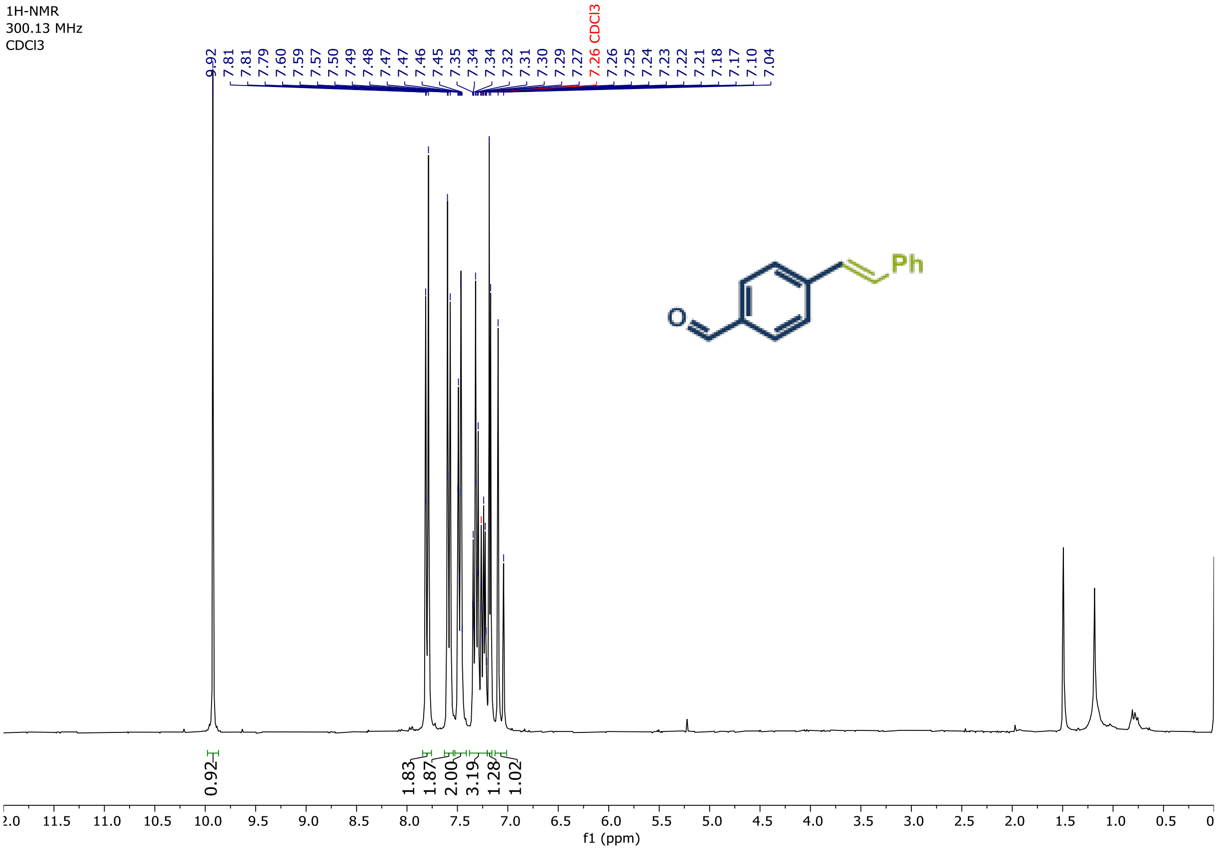

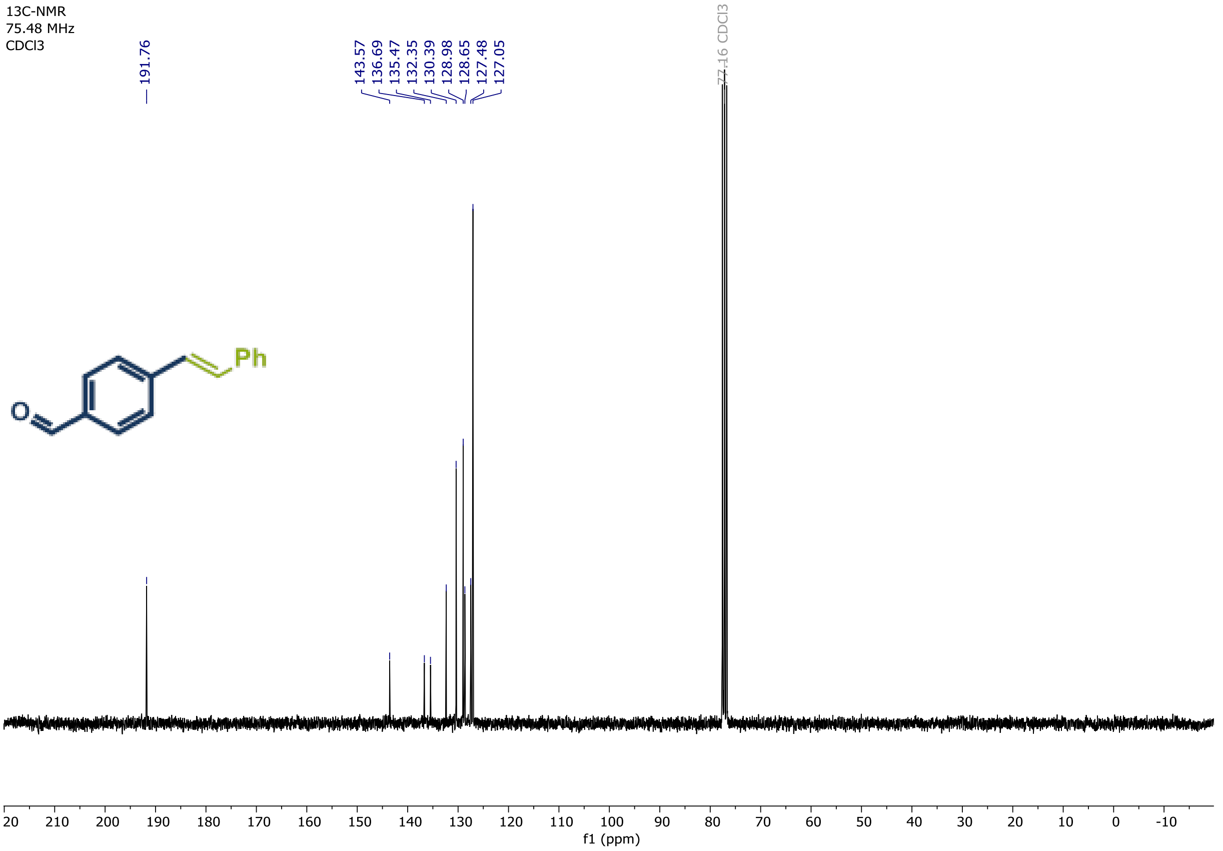

(E)-1-Fluoro-4-styrylbenzene (**3j**)

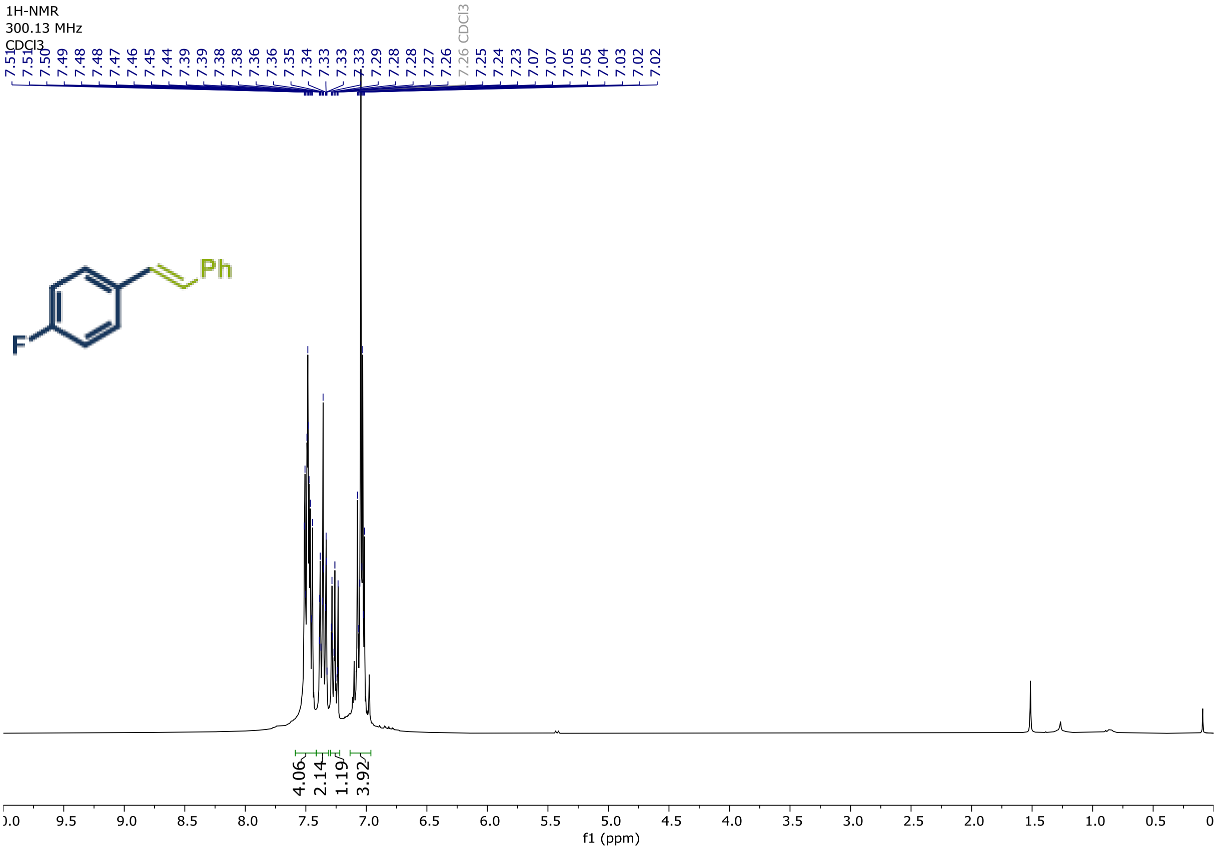

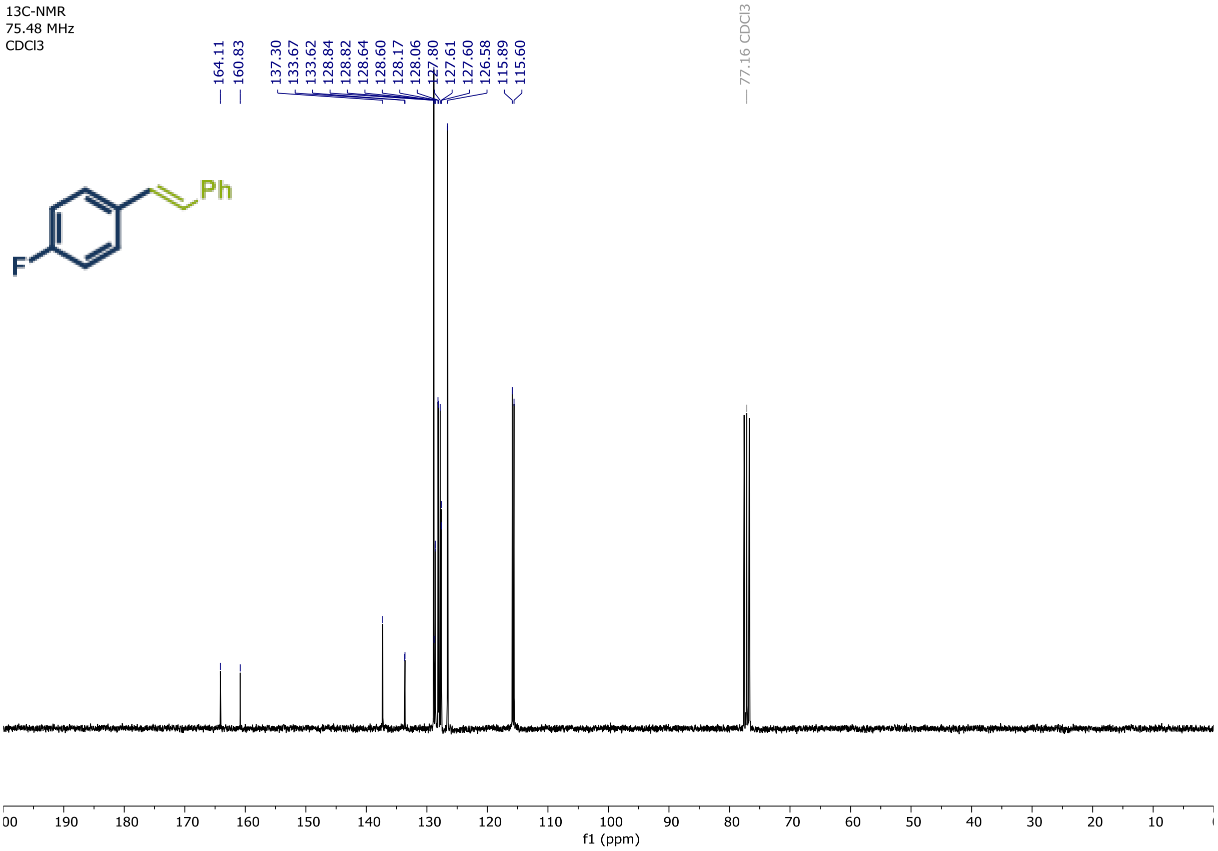

19F-NMR
376.47 MHz
CDCl3

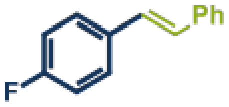

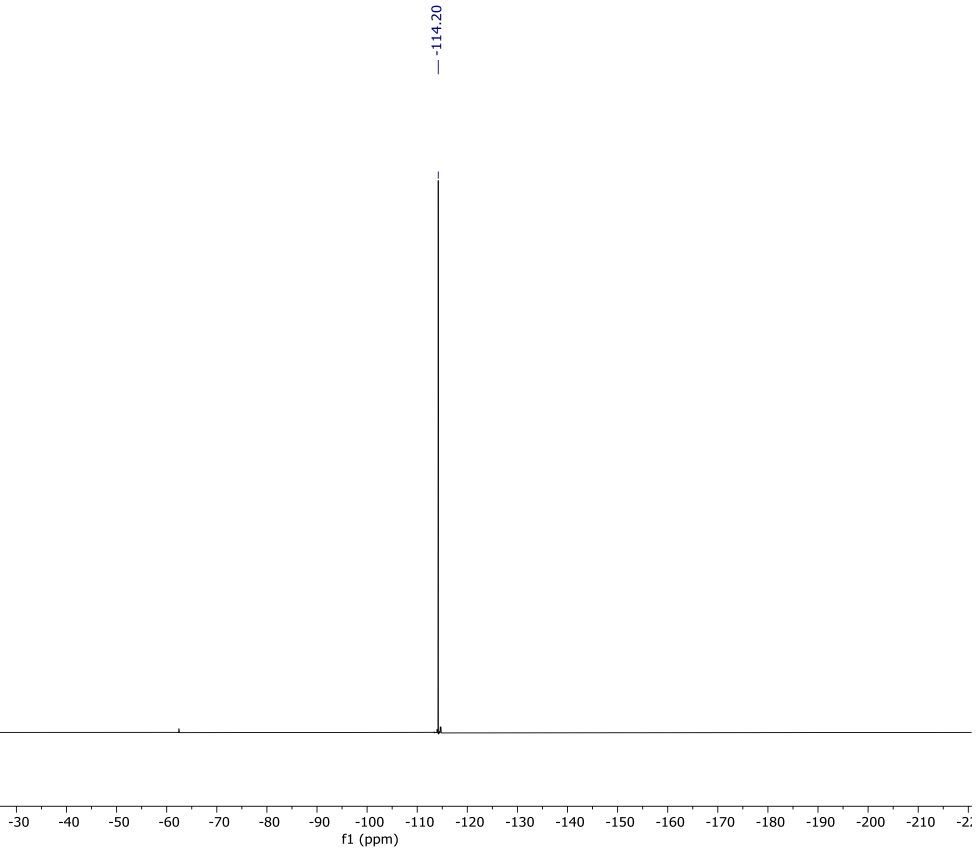

(E)-1-Bromo-4-styrylbenzene (**3k**)

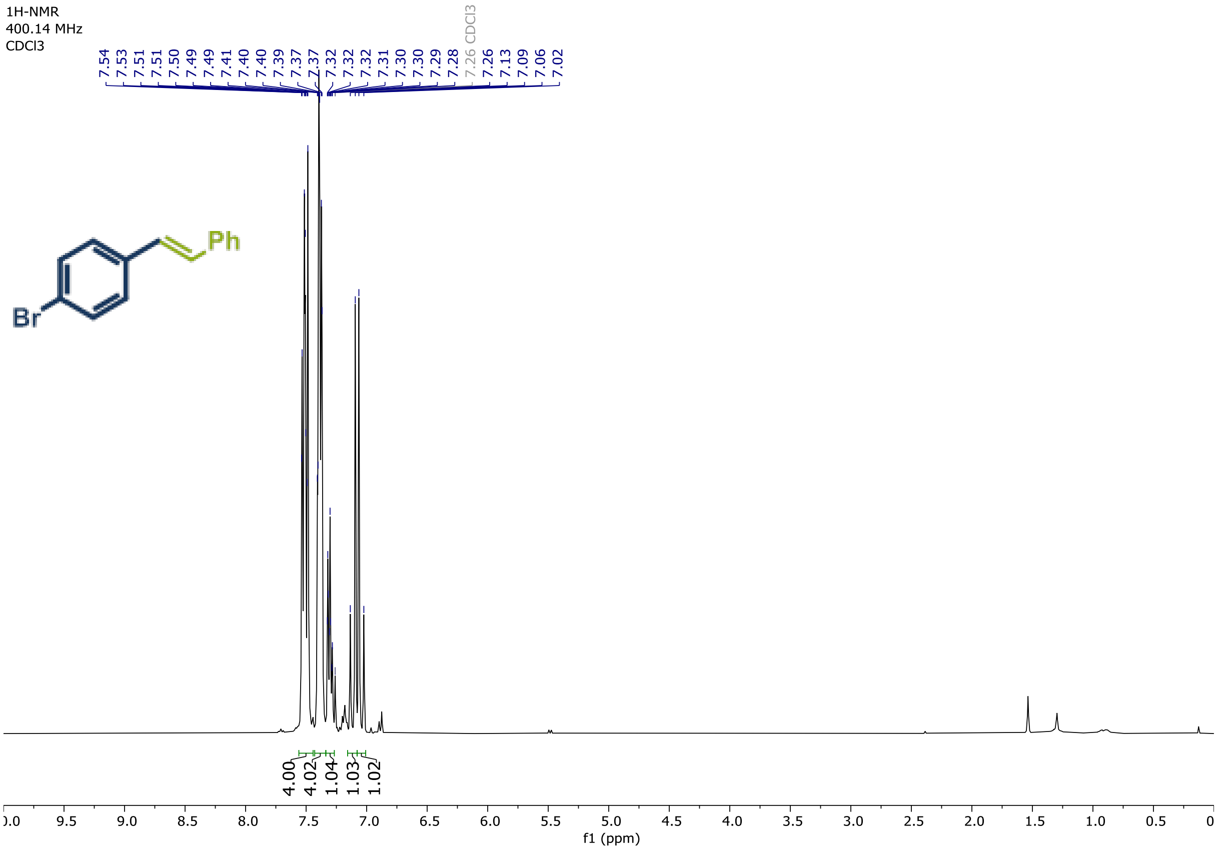

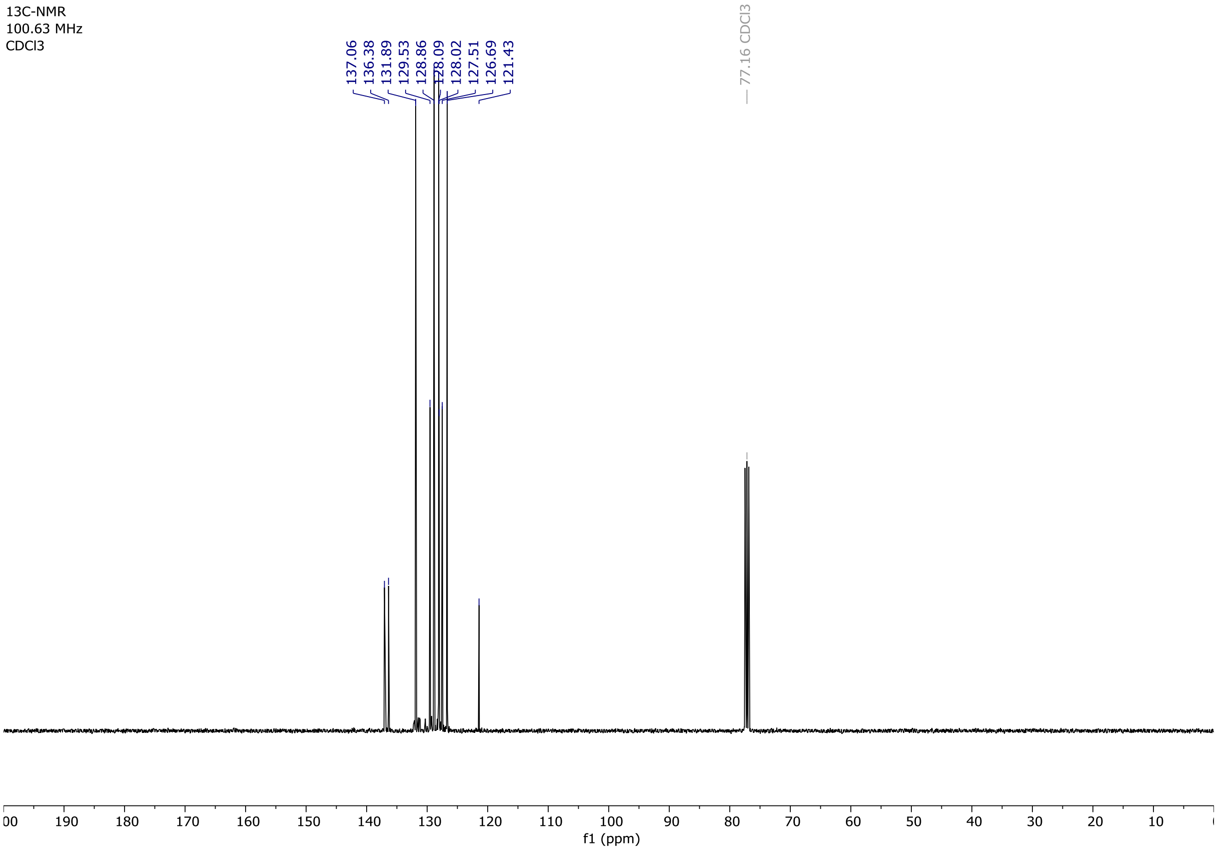

(E)-1-cyano-4-styryl-benzene (**3l**)

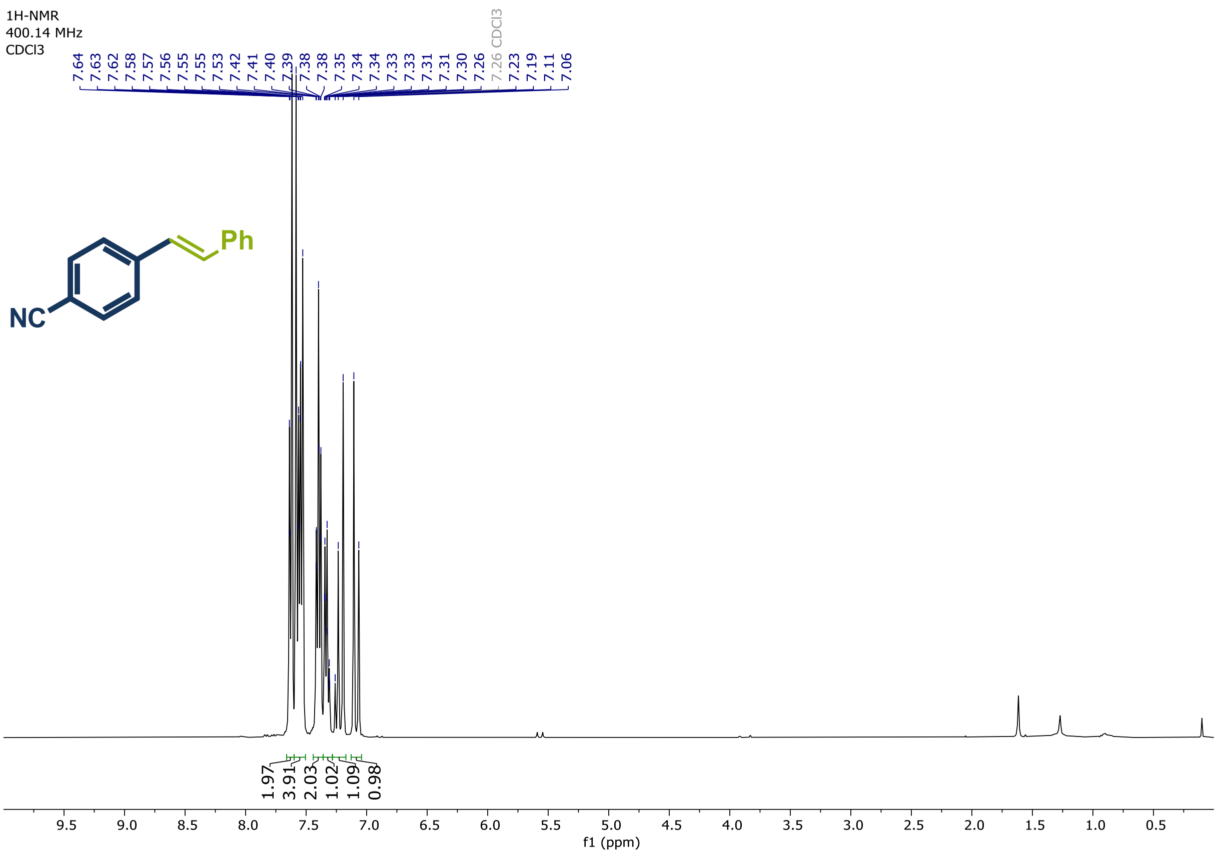

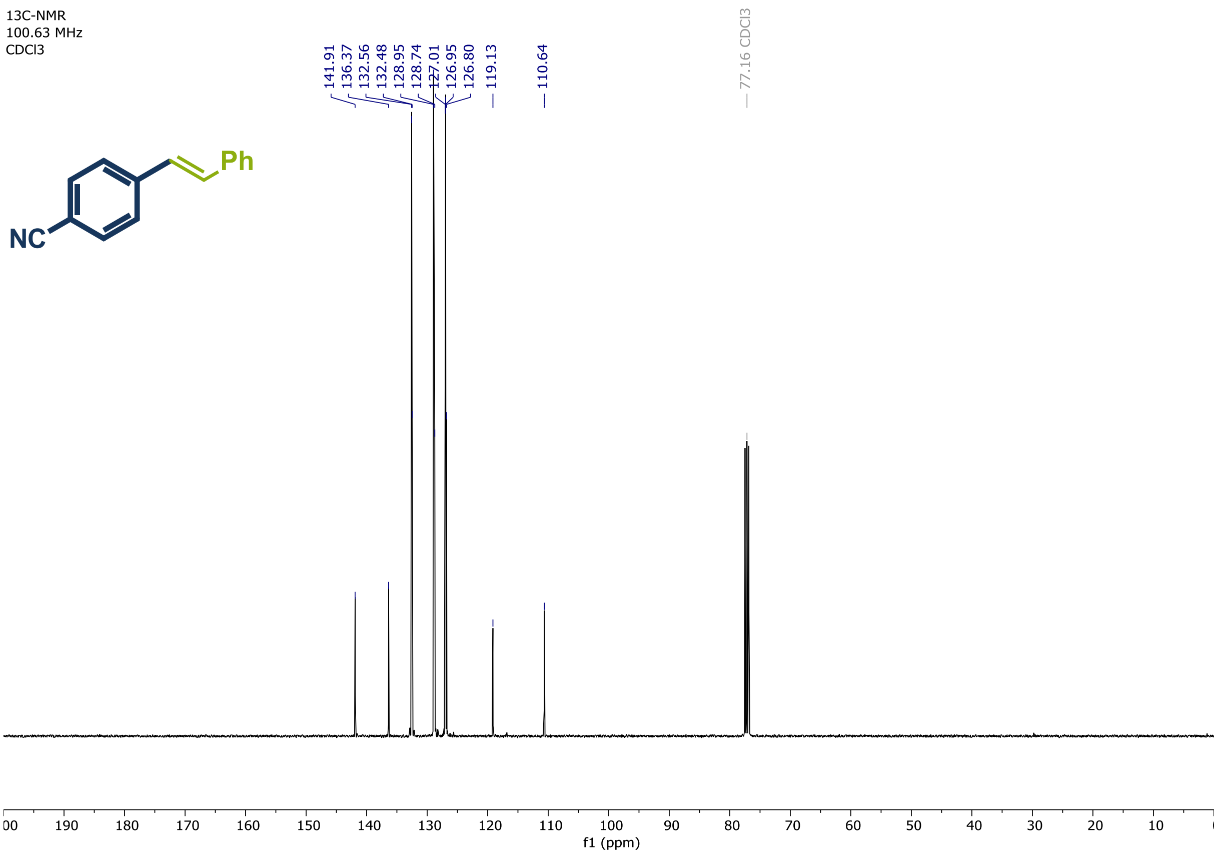

Tert-Butyl *(E)*-3-(4-bromophenyl)acrylate (**3m**)

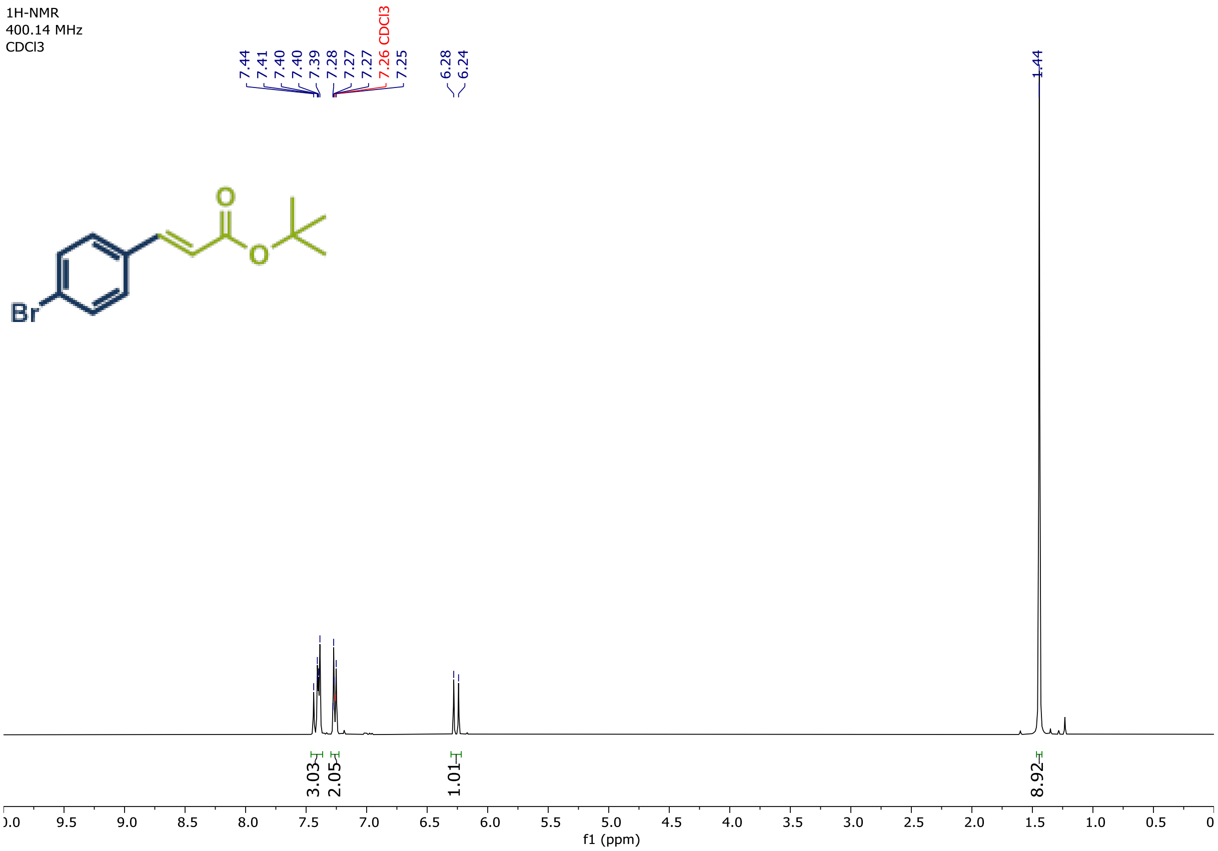

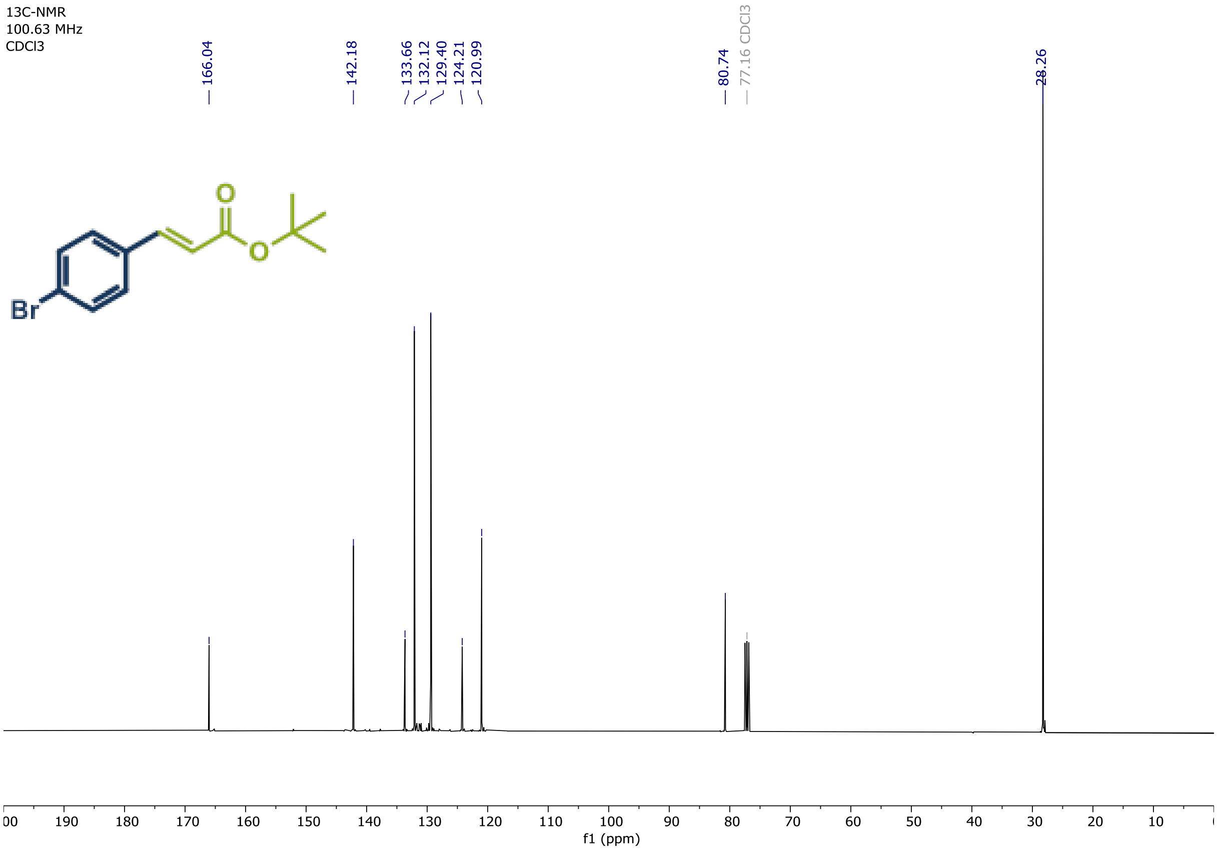

(E)-1-phenoxy-4-styrylbenzene (**3o**)

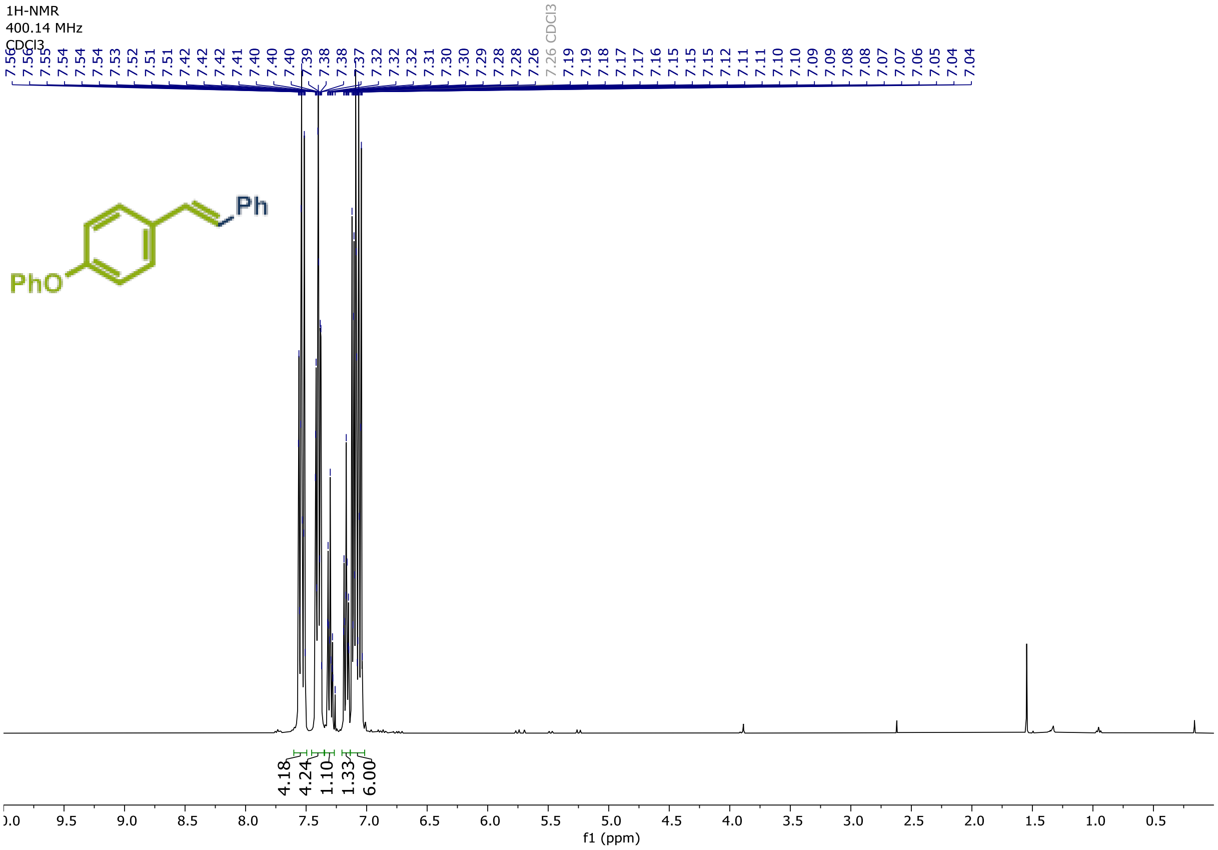

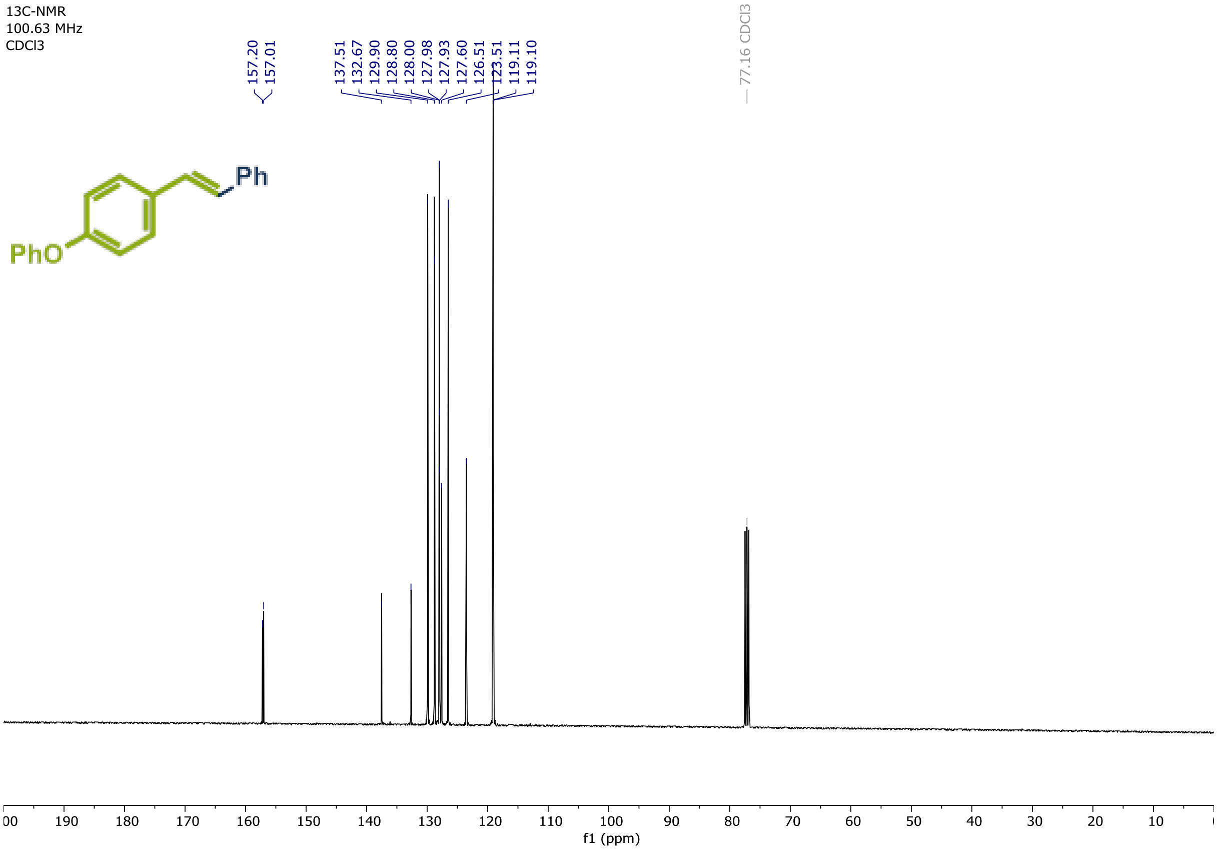

(*E*)-4-phenylstilbene (**3p**)

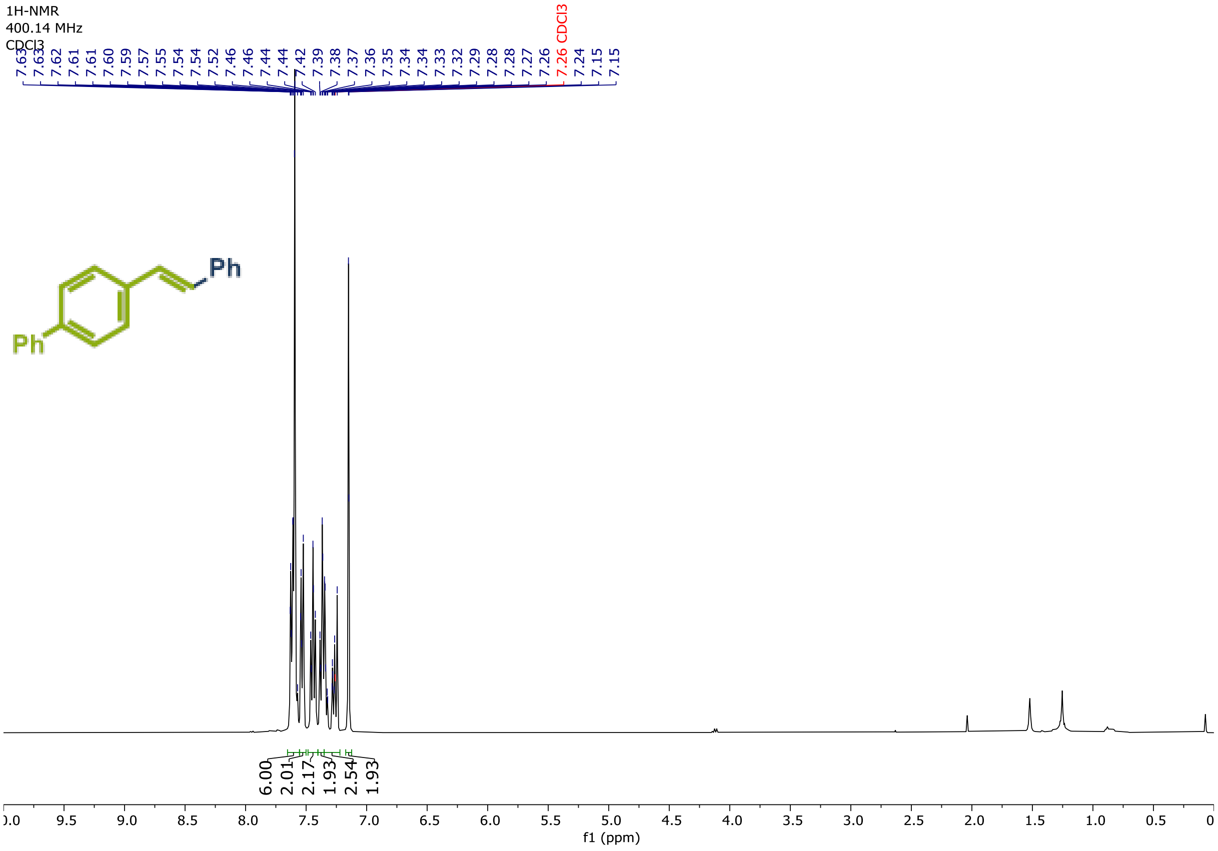

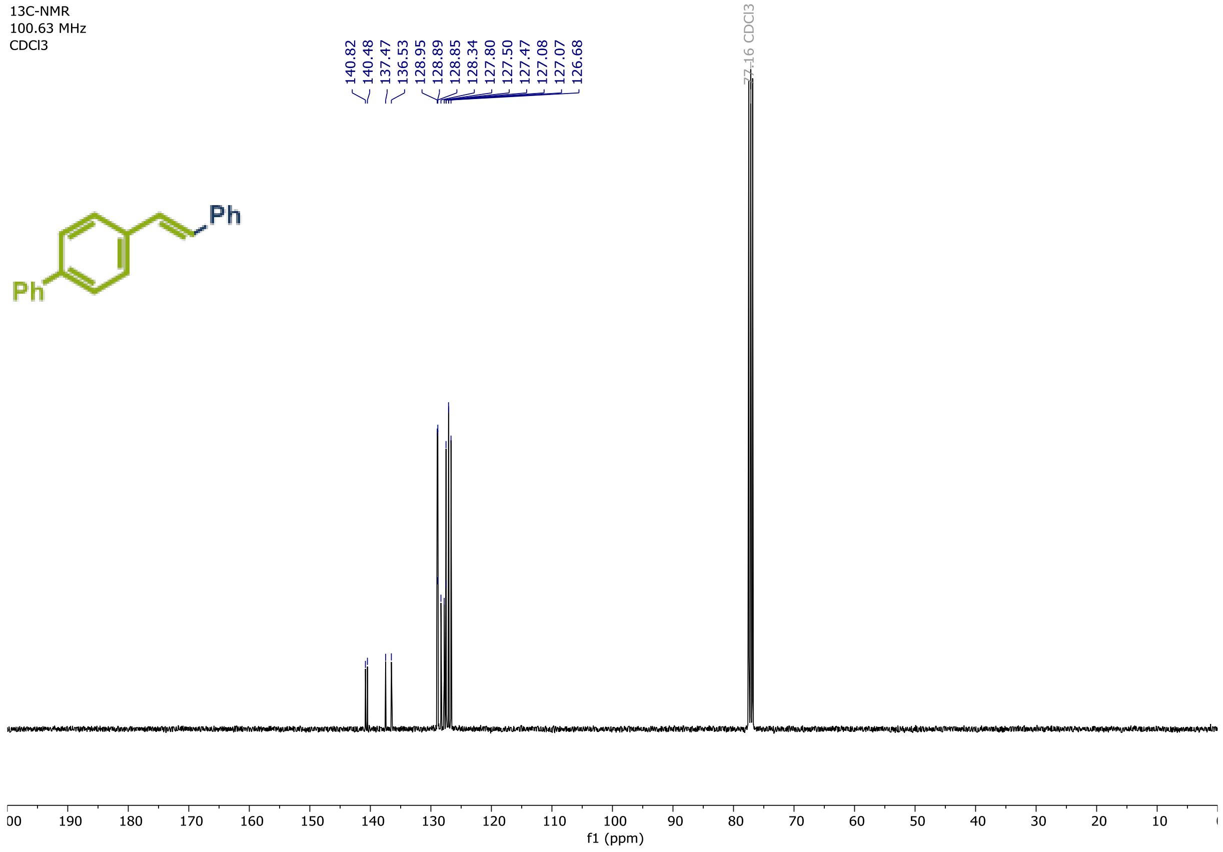

(E)-2-styrylnaphthalene (**3q**)

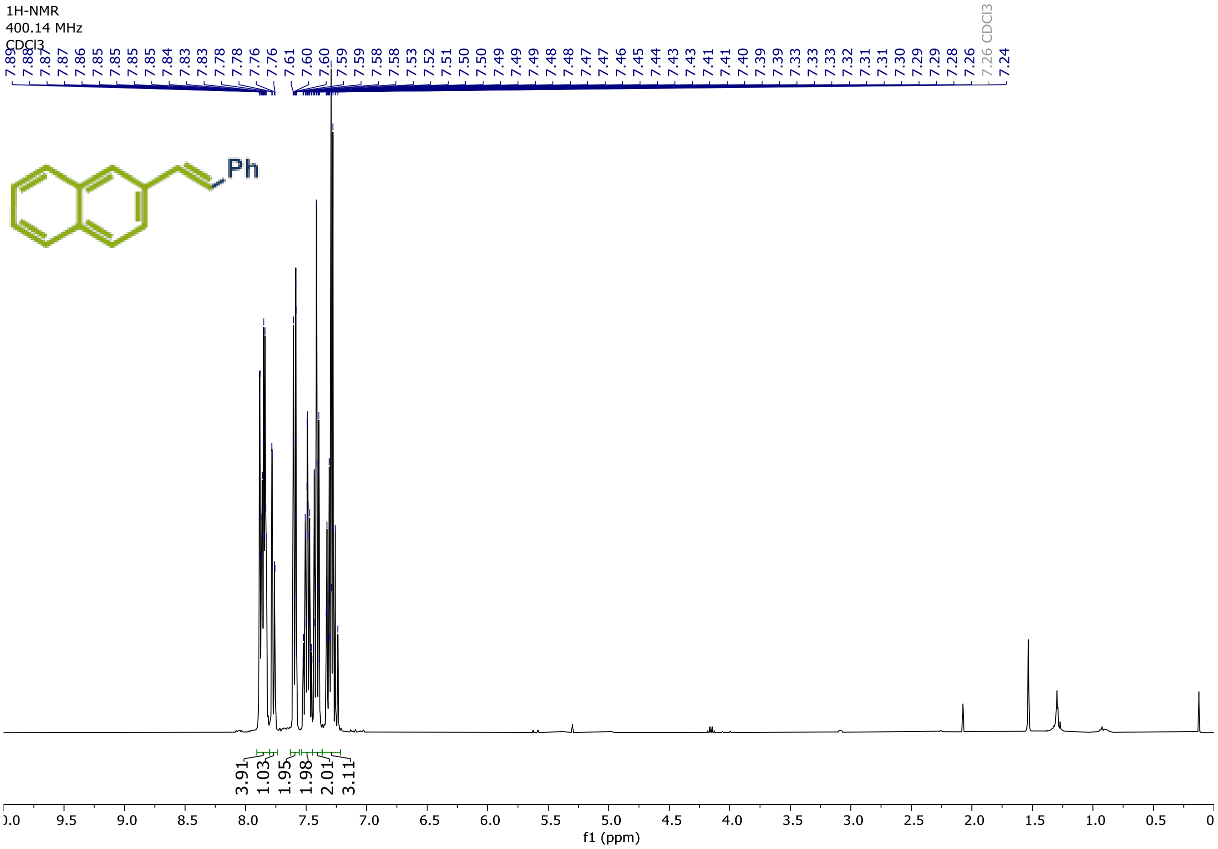

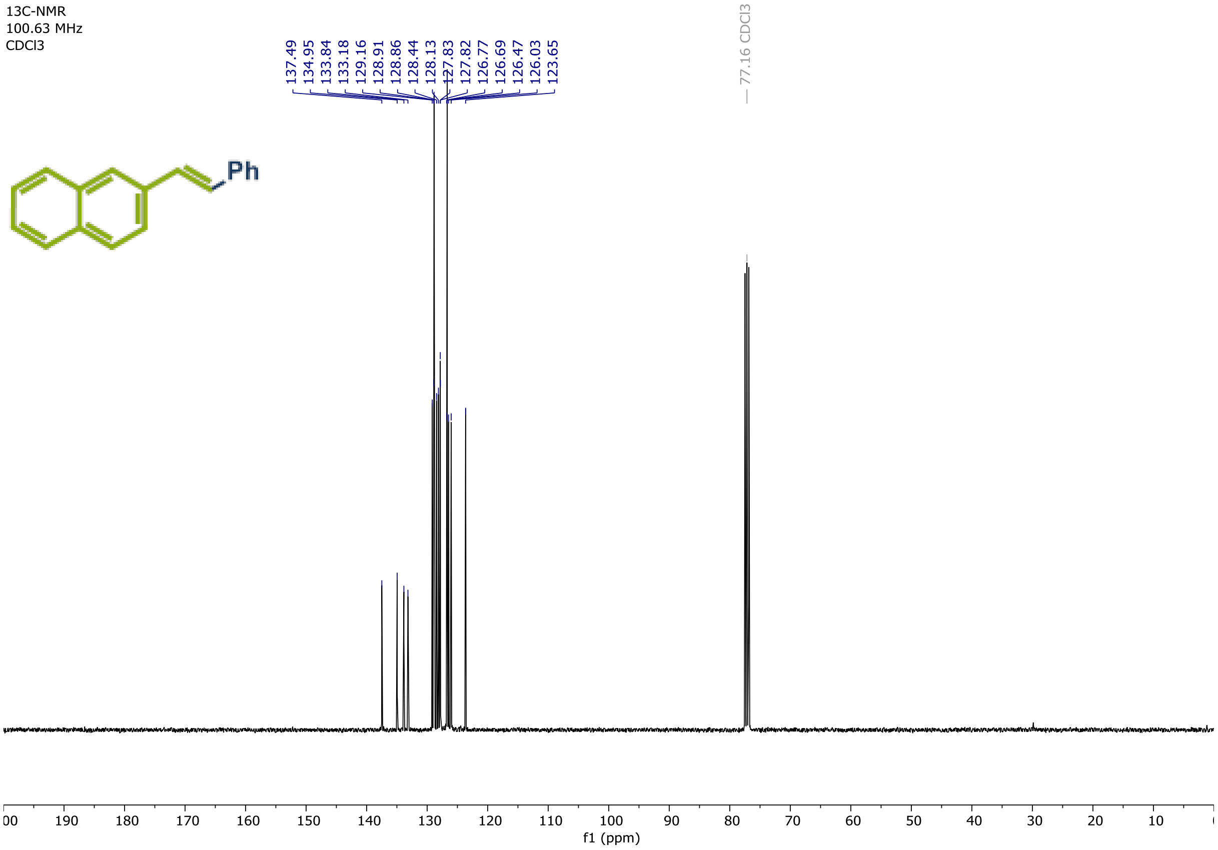

(E)-4-styrylpyridine (**3r**)

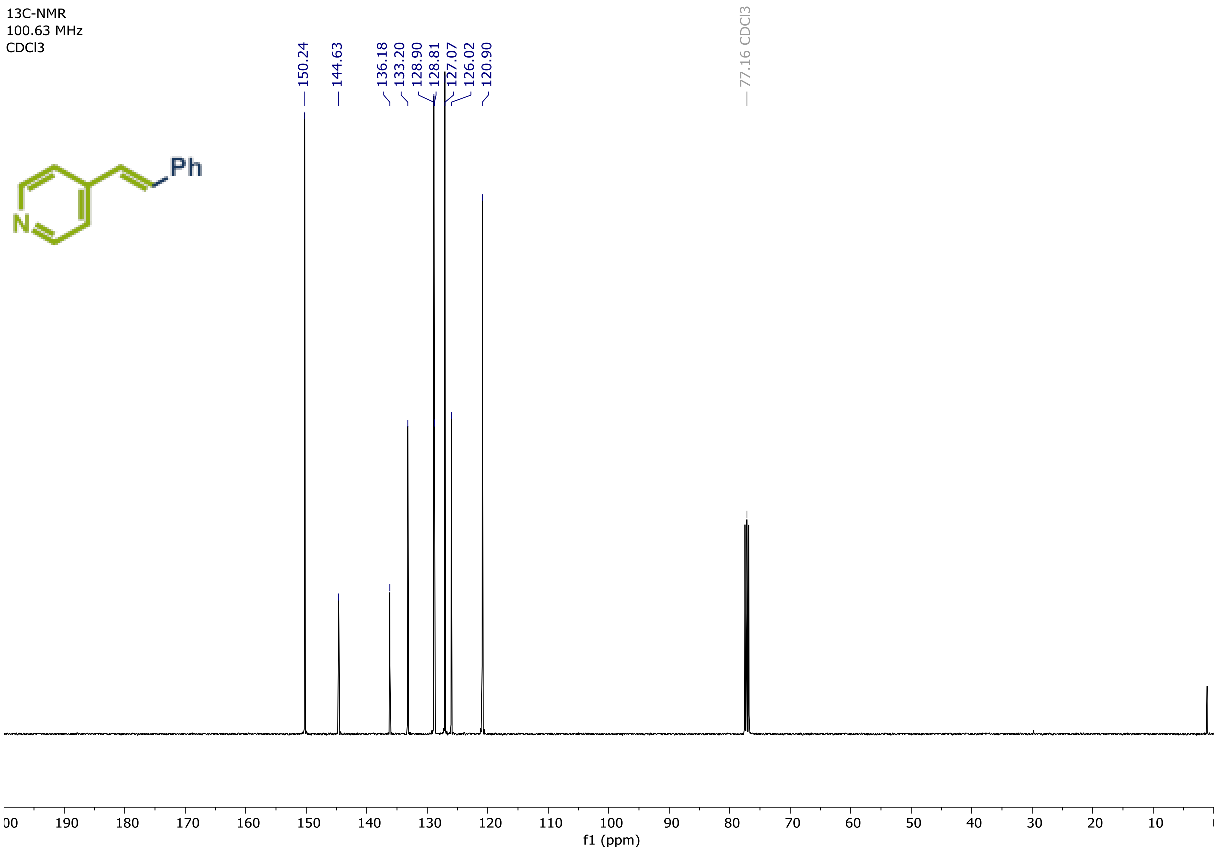

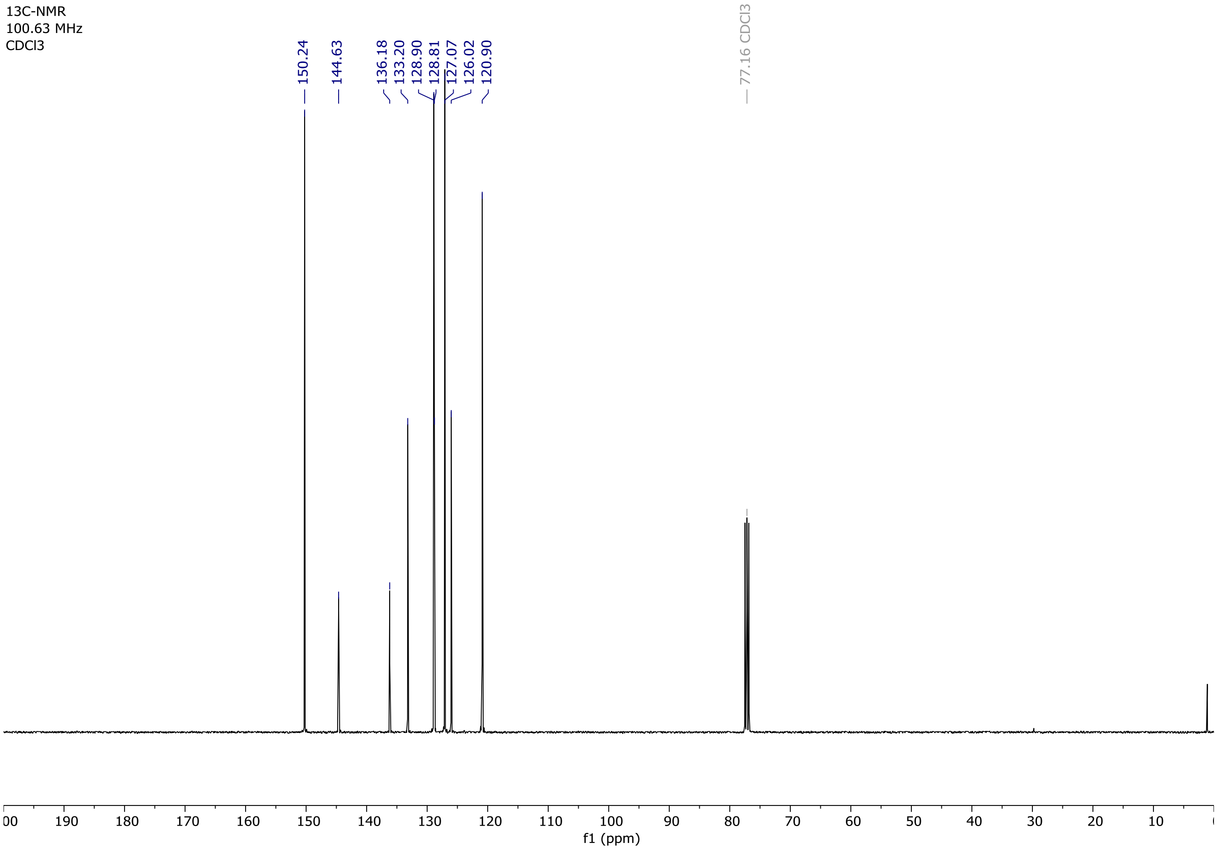

(E)-1-Styryl-4-(trifluoromethyl)benzene (**3s**)

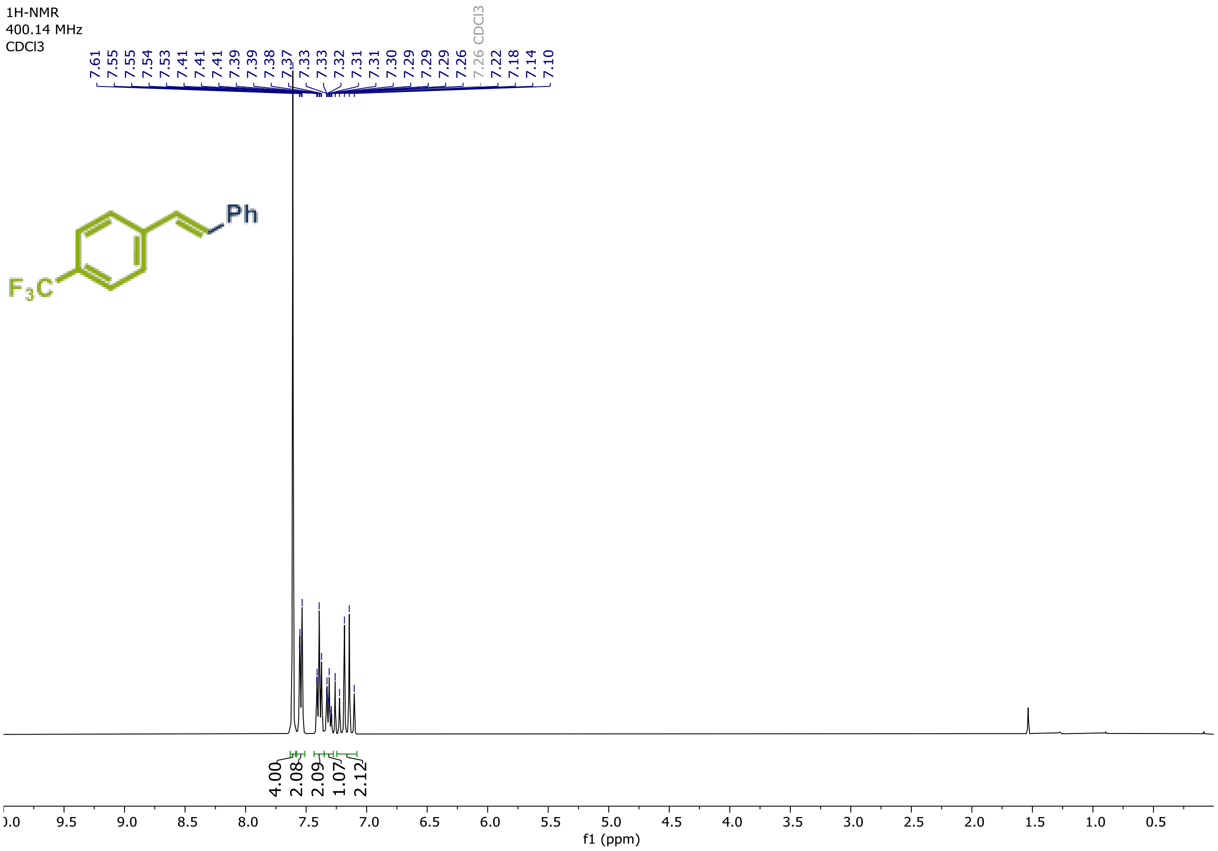

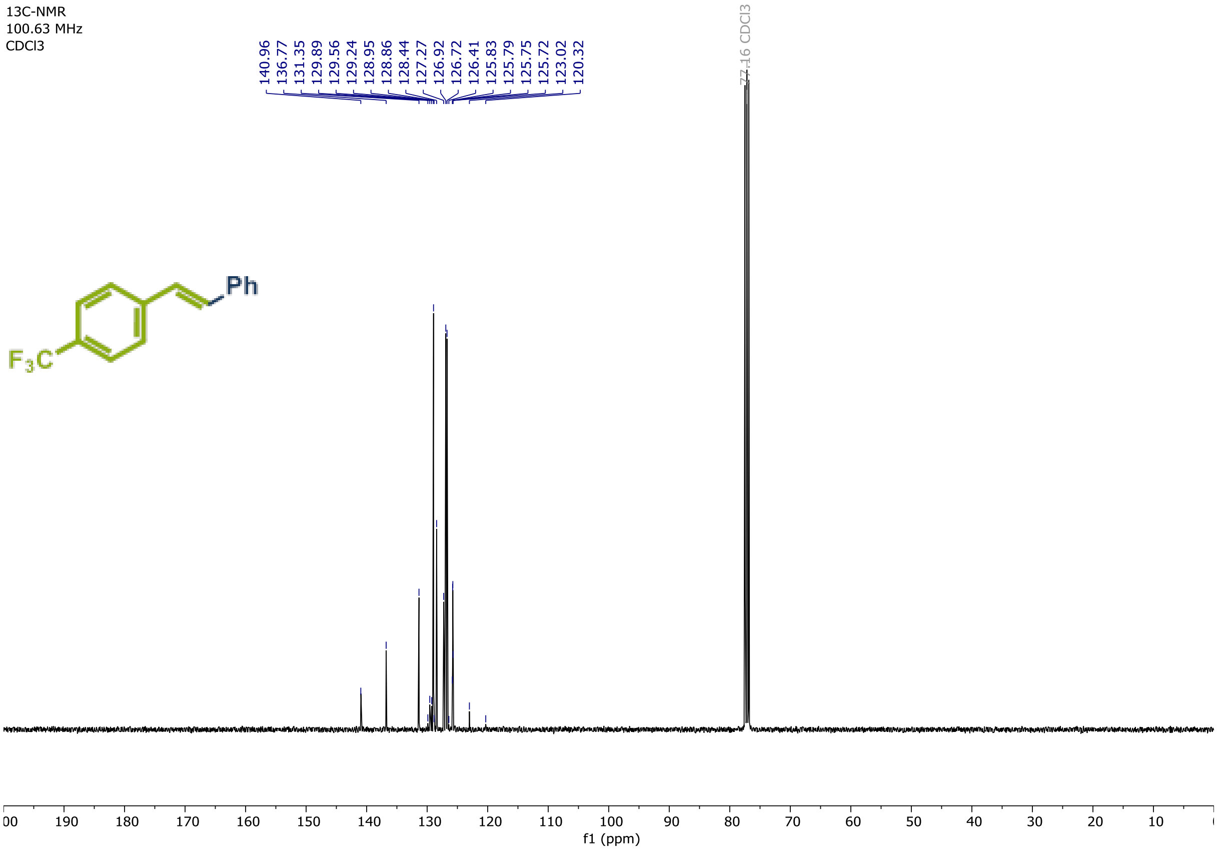

(E)-3,4-dimethoxystilbene (**3t**)

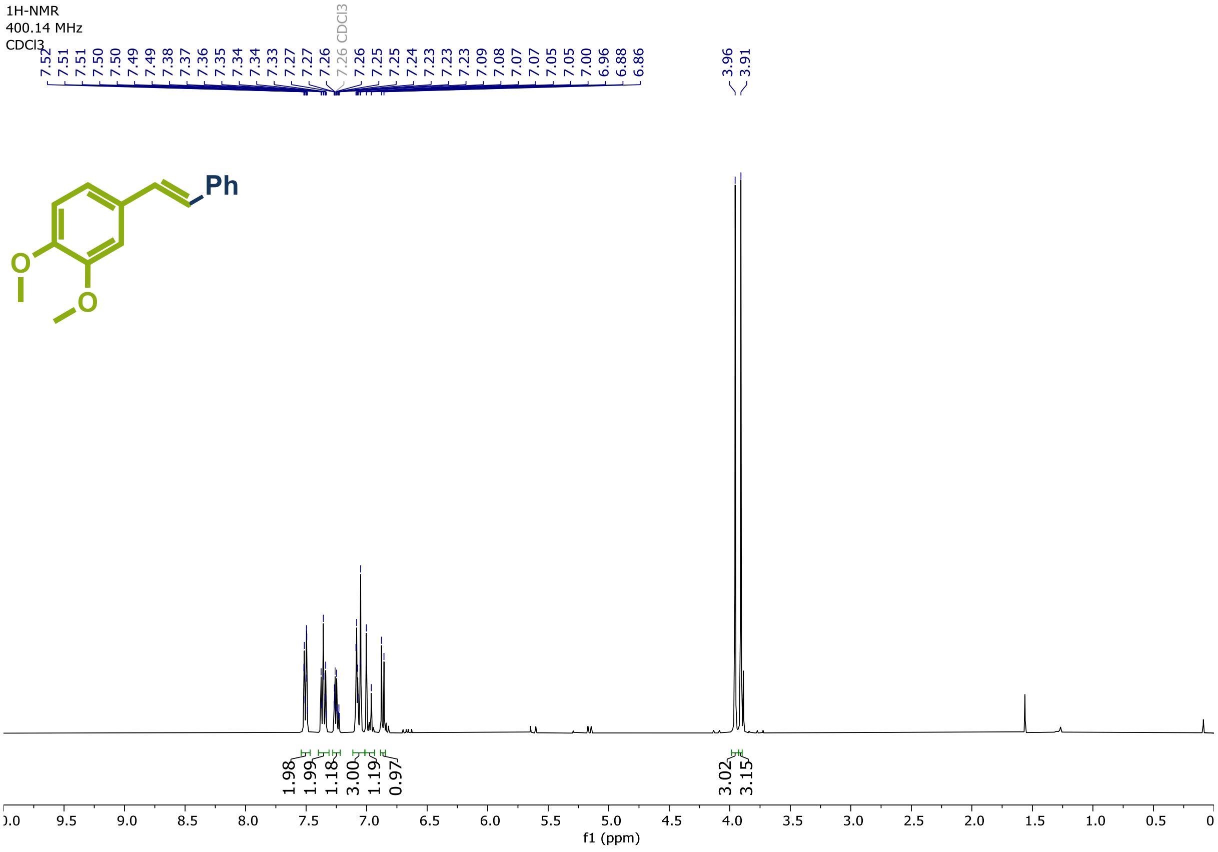

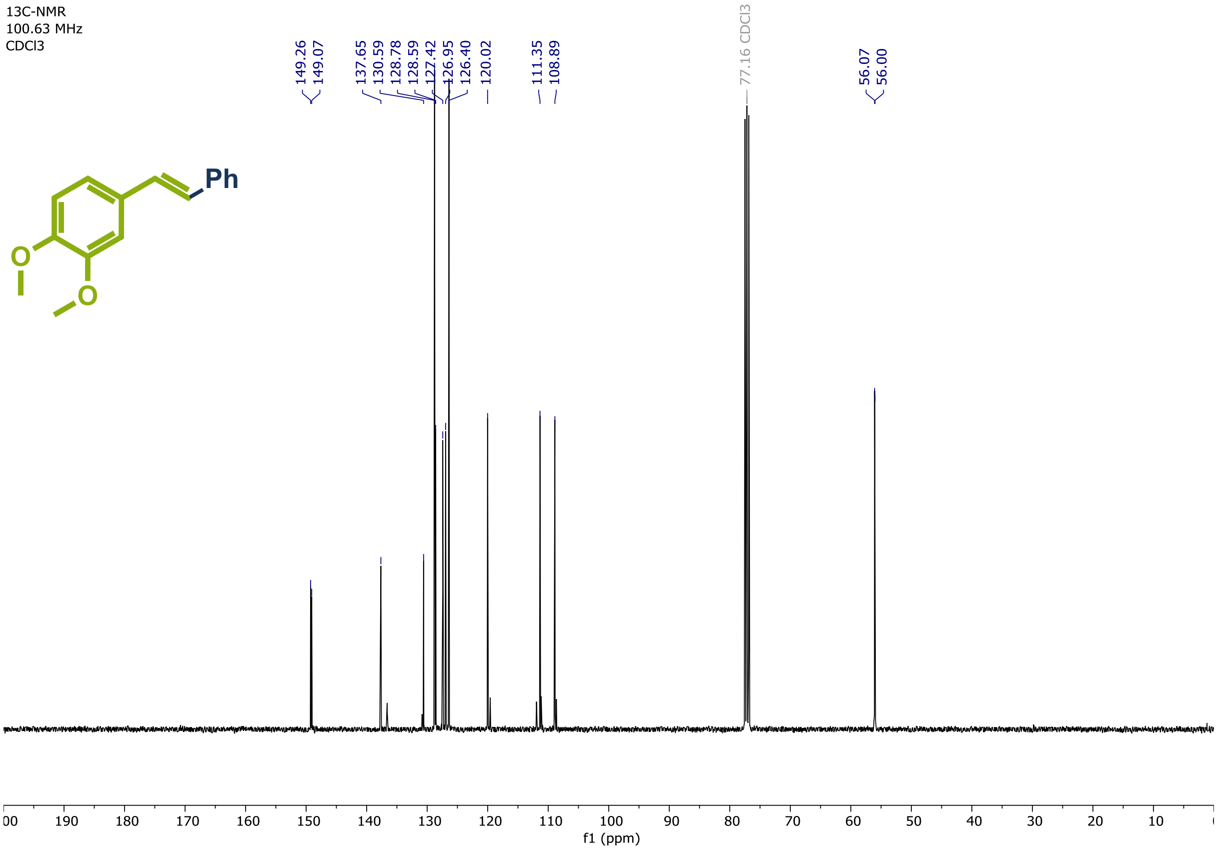

Tert-butyl cinnamate (**3u**)

1H-NMR
400.14 MHz
CDCl3

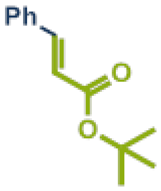

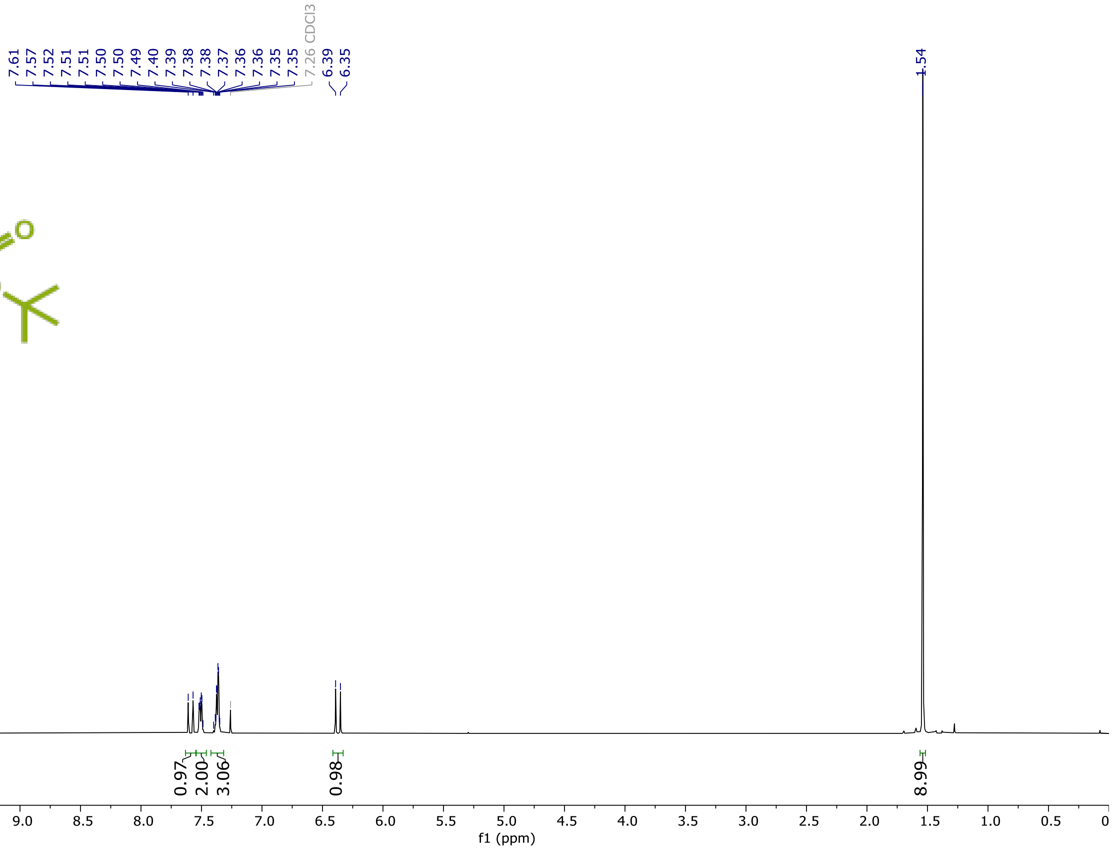

}13C-NMR
100.63 MHz
CDCl3

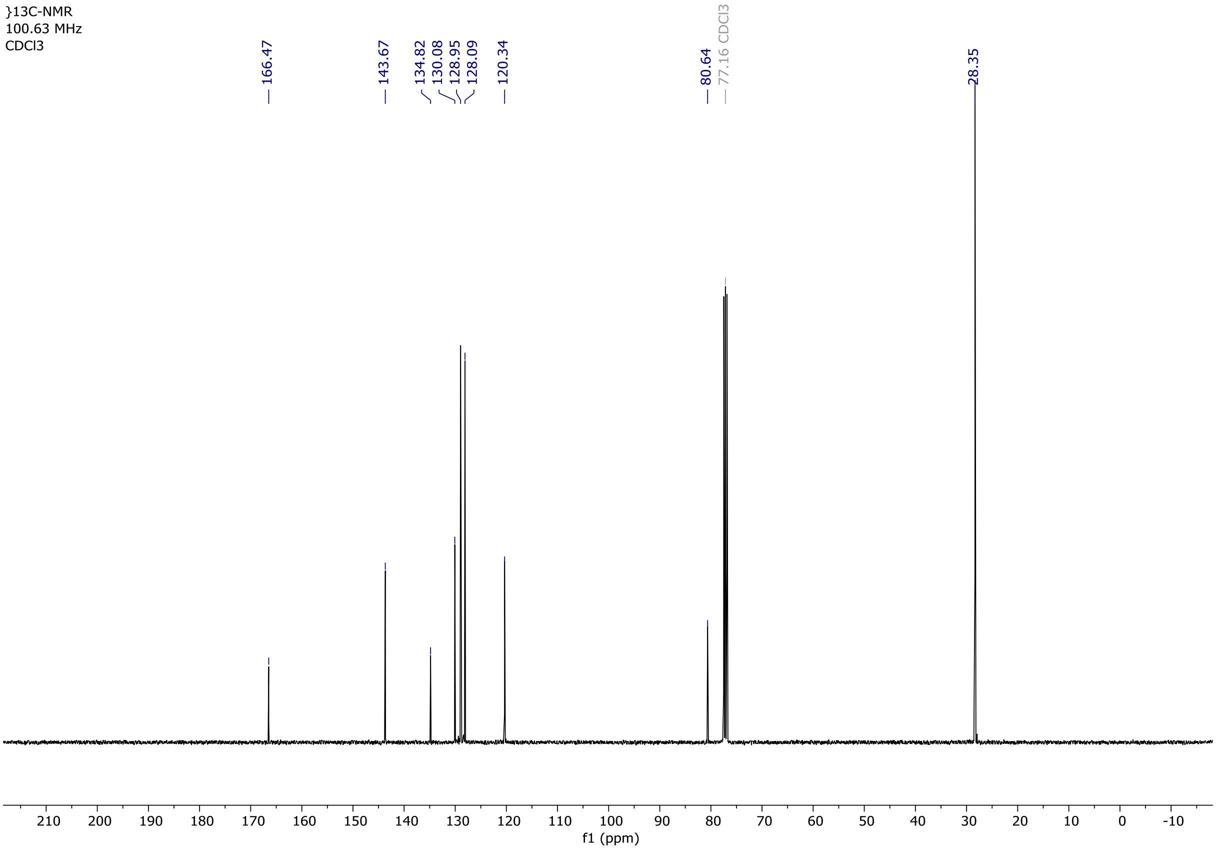

N-Morpholinocinnamide (**3v**)

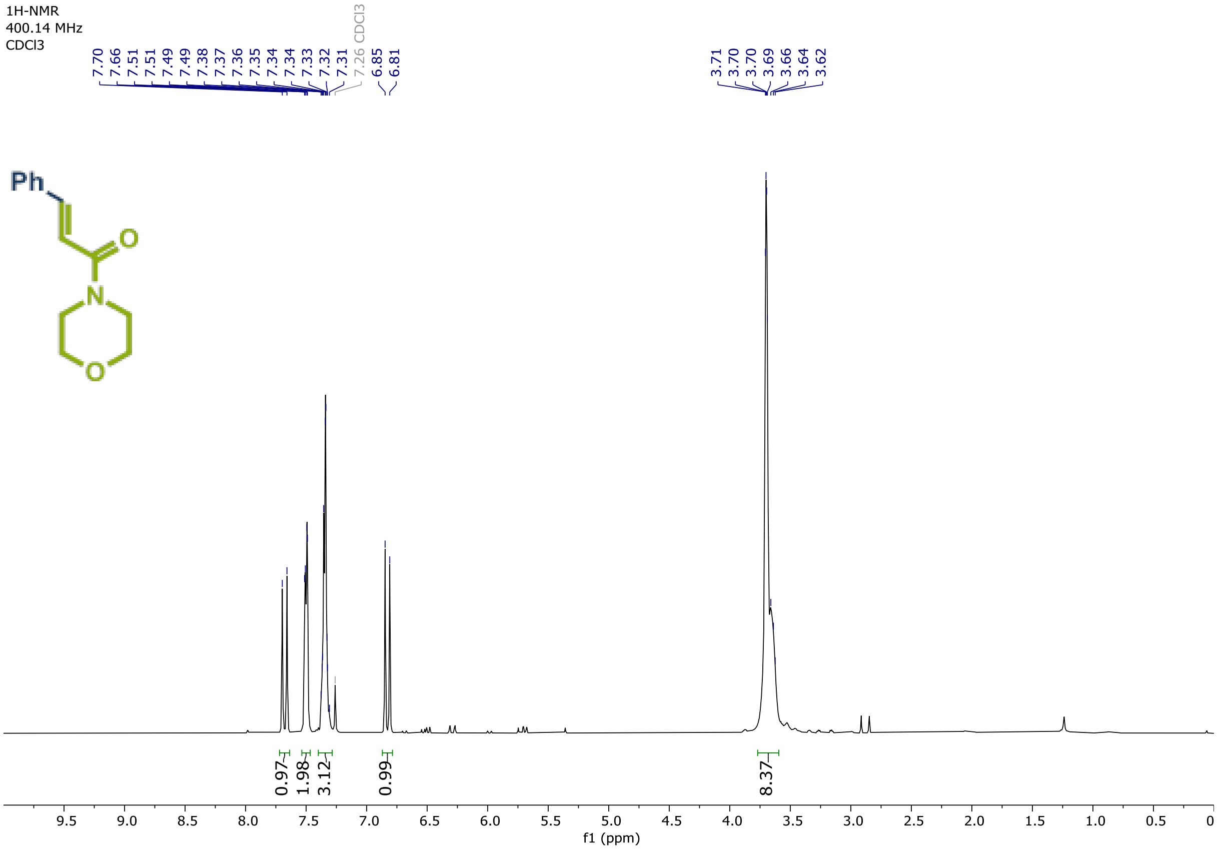

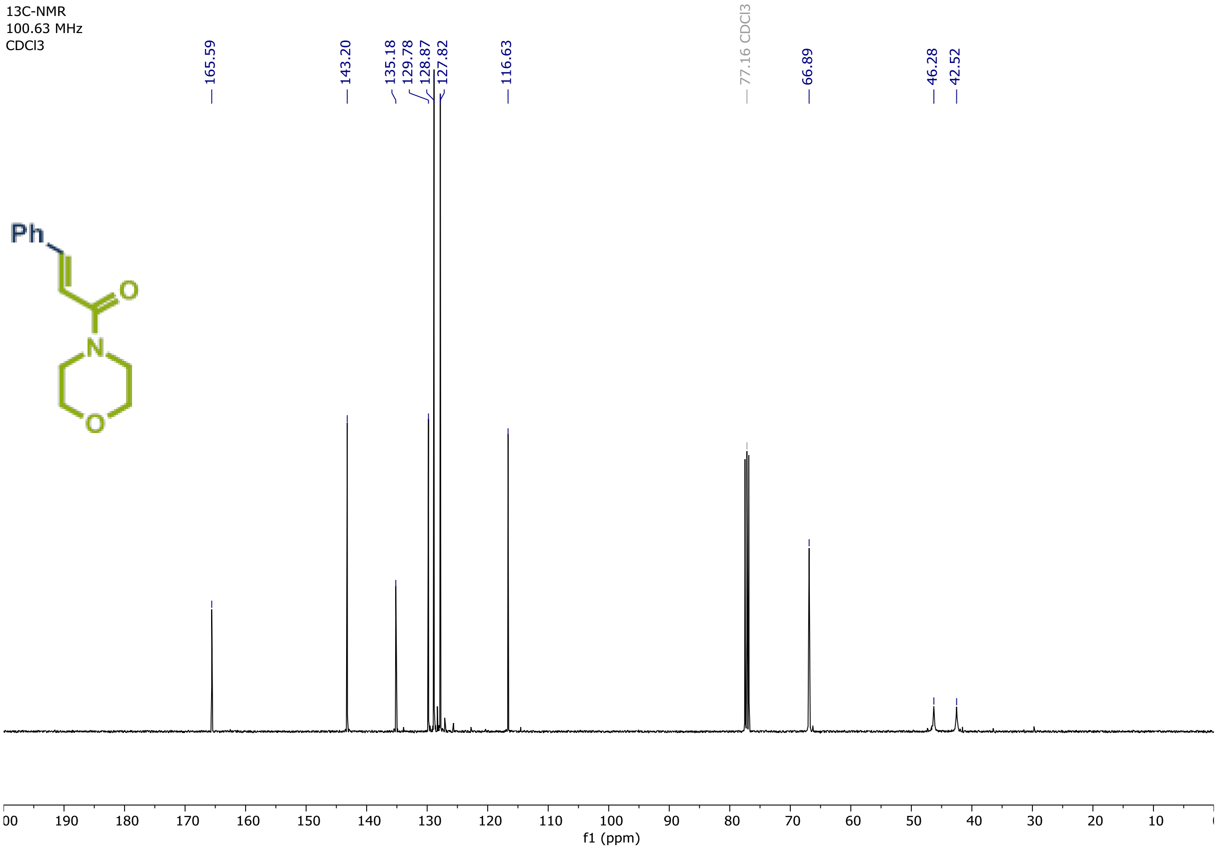

Hexylcinnamate (**3w**)

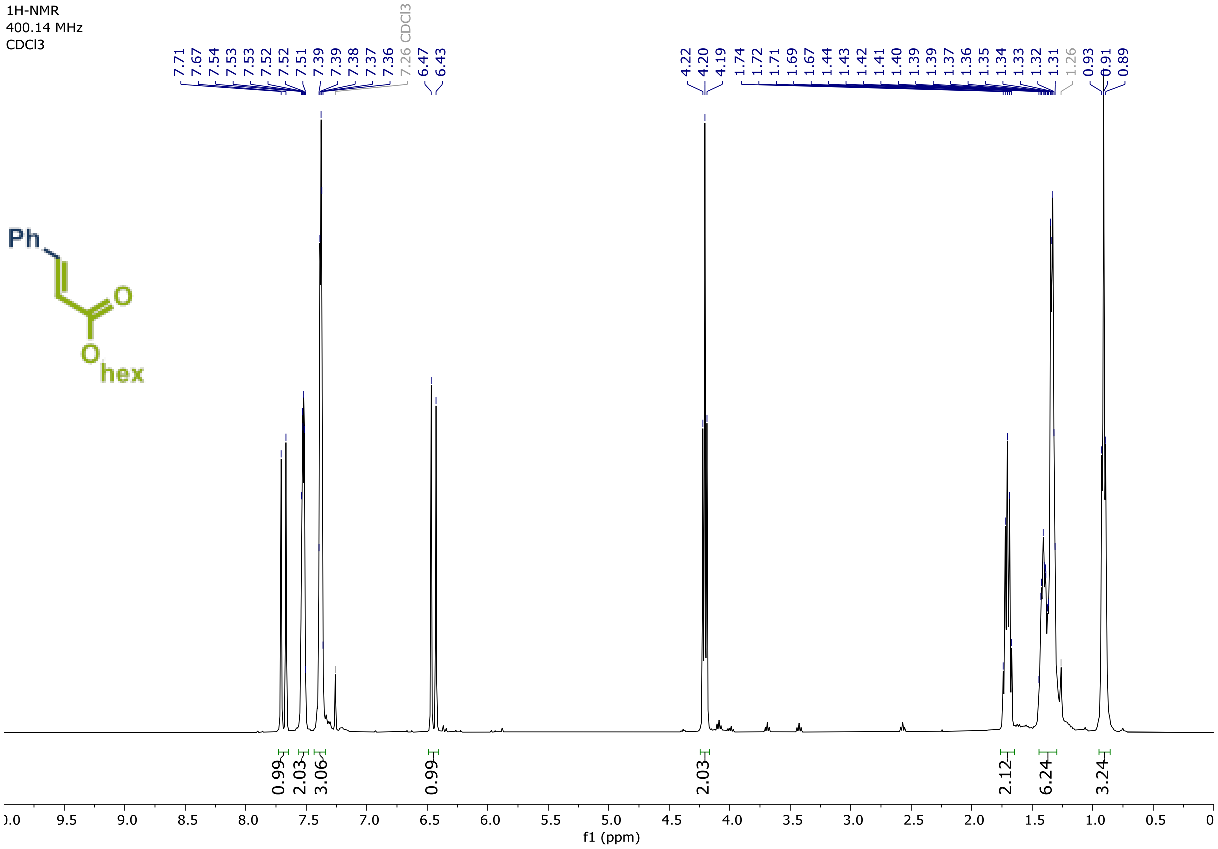

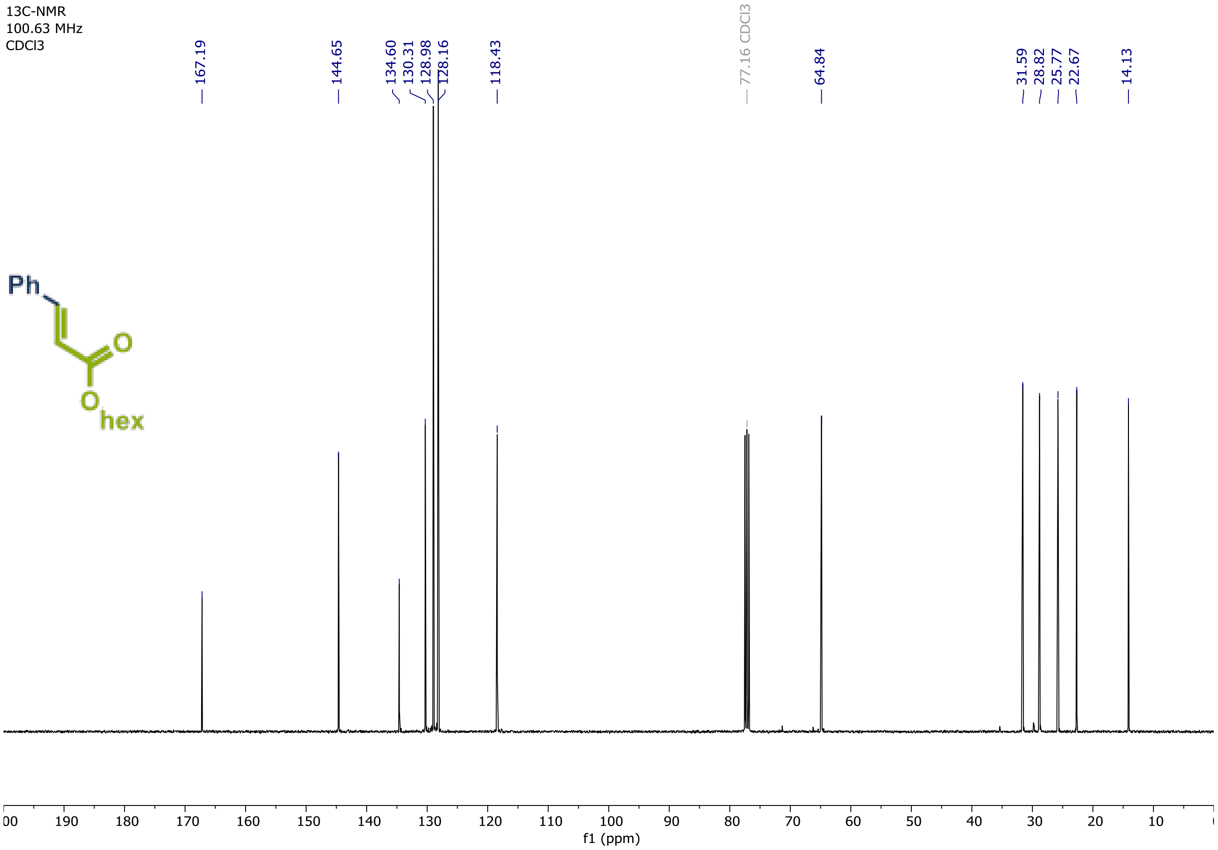

(E)-1-Chloro-4-styrylbenzene (**3x**)

1H-NMR
400.14 MHz
CDCl3

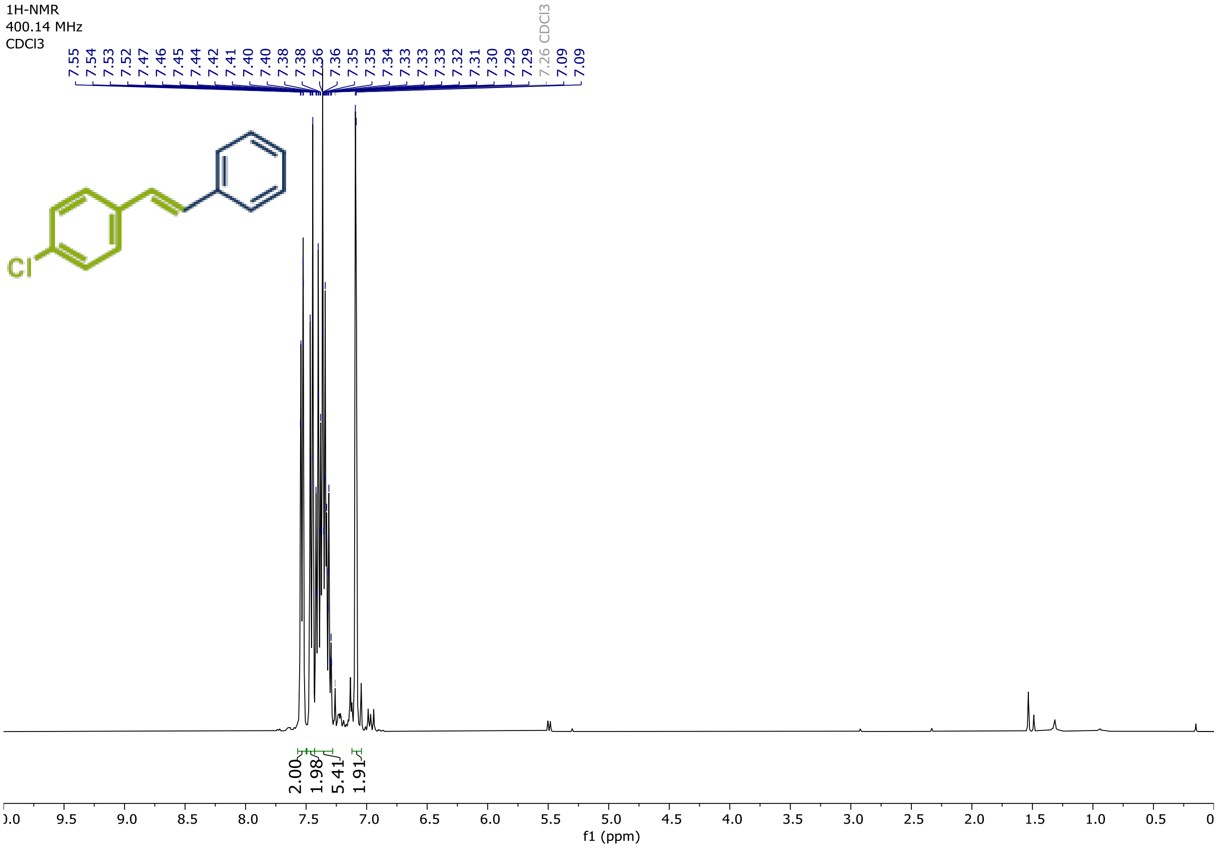

13C-NMR
100.63 MHz
CDCl3

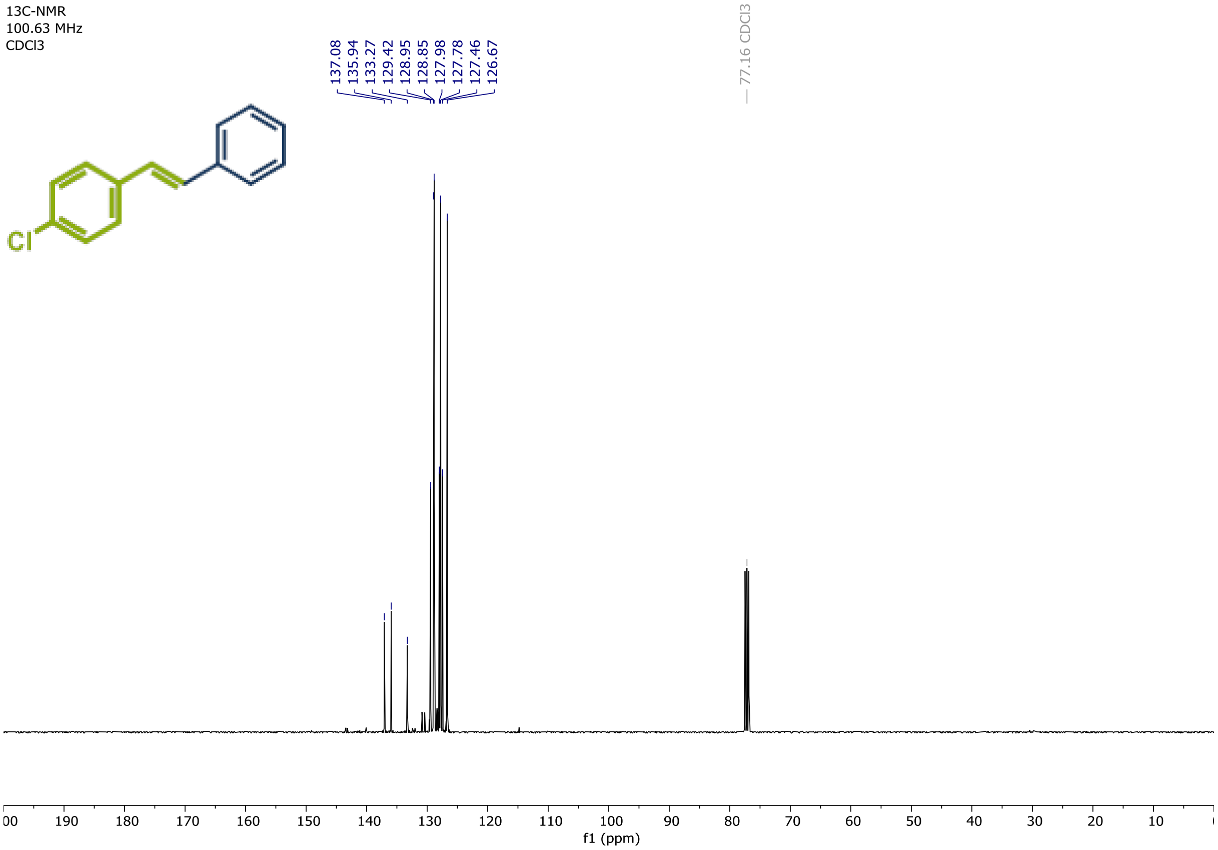

6. References:

1. A. K. Guin, S. Chakraborty, S. Khanra, S. Chakraborty and N. D. Paul, *Organic Letters*, 2024, **26**, 2540-2545.

2. D. B. Paixão, E. G. O. Soares, C. D. G. Silva, T. J. Peglow, D. S. Rampon and P. H. Schneider, *The Journal of Organic Chemistry*, 2023, **88**, 17037-17046.

3. S.-M. Wang, H.-X. Song, X.-Y. Wang, N. Liu, H.-L. Qin and C.-P. Zhang, *Chemical Communications*, 2016, **52**, 11893-11896.

4. Y. Wang, J. Chen, J. Yang, Z. Jiao and C.-Y. Su, *Angewandte Chemie International Edition*, 2023, **62**, e202303288.

5. E. S. Isbrandt, D. E. Chapple, N. T. P. Tu, V. Dimakos, A. M. M. Beardall, P. D. Boyle, C. N. Rowley, J. M. Blacquiere and S. G. Newman, *Journal of the American Chemical Society*, 2024, **146**, 5650-5660.

6. P. K. Pandey, M. Patra, P. Ranjan, N. Kumar Pal, S. Choudhary and J. K. Bera, *Chemistry – A European Journal*, 2024, **30**, e202400337.

7. X. Wang, L. Sun, M. Wang, G. Maestri, M. Malacria, X. Liu, Y. Wang and L. Wu, *European Journal of Organic Chemistry*, 2022, **2022**, e202200009.

8. M. Oberholzer and C. M. Frech, *Green Chemistry*, 2013, **15**, 1678-1686.

9. T. Hu, M. Jaber, G. Tran, D. Bouyssi, N. Monteiro and A. Amgoune, *Chemistry – A European Journal*, 2023, **29**, e202301636.

10. A. Bourboula, O. G. Mountanea, G. Krasakis, C. Mantzourani, M. G. Kokotou, C. G. Kokotos and G. Kokotos, *European Journal of Organic Chemistry*, 2023, **26**, e202300008.

11. S. Zhan, X. Tao, L. Cai, X. Liu and T. Liu, *Green Chemistry*, 2014, **16**, 4649-4653.
